# Supplementary material for: Multilevel analysis of predictors of multiple indicators of childhood vaccination in Nigeria
Source: PLoS One. 2022 May 25;17(5):e0269066. doi: 10.1371/journal.pone.0269066 (PMC9132327; doi:10.1371/journal.pone.0269066)
Supplement: S1 File — Supplementary file containing Tables A–M, Figs A-D and additional texts referenced in the manuscript. (DOCX) [file pone.0269066.s001.docx]

**Multilevel analysis of predictors of multiple indicators of childhood vaccination in Nigeria**

Justice M. K. Aheto, Oliver Pannell, Winfred Dotse-Gborgbortsi, Mary K. Trimner, Andrew J. Tatem, Dale A. Rhoda, Felicity T. Cutts, C. Edson Utazi

**Supporting Information**

This supplementary information accompanies the main manuscript and contains Tables A–M, Figs A-D and additional texts referenced in the main manuscript.

**Description and coding of outcome variables and covariate factors**

**Table A: Description and coding of outcome variables and covariate factors**

| **Variable name and labels** | **Description and coding** | **Reference category** |
| --- | --- | --- |
| **Outcome variables (binomial)** | | |
| Received PENTA1 | No/don’t know – 0, reported by mother/ vaccination date on card/ vaccination marked on card - 1 | No/don’t know – 0 |
| Received PENTA3/1 | Received PENTA1 but not PENTA3 – 0, Received both - 1 | Received PENTA1 but not PENTA3 – 0 |
| Received MV | No/don’t know – 0, reported by mother/ vaccination date on card/ vaccination marked on card - 1 | No/don’t know – 0 |
| **Outcome variables (multinomial)** | | |
| Received PENTA1 | No evidence of vaccination – 0, Card invalid/ history – 1, Card valid - 2 | No evidence of vaccination – 0 |
| Received MV | No evidence of vaccination – 0, Card invalid/ history – 1, Card valid - 2 | No evidence of vaccination – 0 |
| **Predictor variables** | | |
| Sex of child | Male – 0, female – 1 | Male – 0 |
| Birth order | 1-2 (1^st^ and 2^nd^ births) – 0, >2 - 1 | 1-2 – 0 |
| Birth quarter | Jan-Mar – 0, Apr-Jun – 1, Jul-Sep – 2, Oct-Dec - 3 | Oct-Dec - 3 |
| Skilled birth attendance | No skilled attendant at birth – 0, Skilled attendant at birth - 1 | No skilled attendant at birth – 0 |
| Health card/document (or home-Based record -HBR) | Does not have health card/document – 0, yes, none seen – 1, yes, seen - 2 | Does not have health card/document – 0 |
| Received vitamin A | No/don’t know – 0, Yes - 1 | No/don’t know – 0 |
| Sex of household head | Male – 0, Female – 1 | Male – 0 |
| Mother's age group | 15-19 – 0, 20-29 – 1, 30-39 – 2, 40-49 – 3 | 15-19 – 0 |
| Marital status of mother | Never in union – 0, married – 1, Divorced – 2 | married – 1 |
| Mother employed in the past 12 months | No – 0, Yes (currently/in the past 1 year) – 1 | No – 0 |
| Mother had problem seeking medical advice or treatment | Did not have problem seeking medical advice or treatment – 0, Had problem seeking medical advice or treatment – 1 | Did not have problem seeking medical advice or treatment – 0 |
| Mother’s education | No education – 0, Primary – 1, Secondary/higher – 2 | No education – 0 |
| Mother’s religion | Islam – 0, Christian – 1, Traditionalist/others – 2 | Islam – 0 |
| Mother’s media exposure | No – 0, Yes (radio/tv/newspaper at least once a week) – 1 | No – 0 |
| Mother’s access to mobile phone/internet | No – 0, Yes – 1 | No – 0 |
| Mother’s land ownership | Does not own land – 0, Owns land alone and/or jointly – 1 | Does not own land – 0 |
| Mother’s knowledge of malaria | Has no knowledge – 0, Has knowledge – 1 | Has no knowledge – 0 |
| Mother had health insurance | No – 0, Yes – 1 | No – 0 |
| Household’s bed net ownership | No – 0, Yes – 1 | No – 0 |
| Mother’s ethnicity | Hausa/Fulani – 0, Yoruba – 1, Igbo – 2, Others (ekoi, ibibio, etc.) – 3 | Hausa/Fulani – 0 |
| Household wealth | Poorer/poorest – 0, Middle – 1, Richer/richest – 2 | Poorer/poorest – 0 |
| Access to bank account | No – 0, Yes – 1 | No – 0 |
| Household size | Large (>=9) – 0, Medium (5 to 8) – 1, Small (<=4) – 2 | Medium (5 to 8) – 1 |
| Length of stay in household | <1year/visitor – 0, 1-3years – 1, 4-5years – 2, >5years/always – 3 | 4-5years – 2 |
| Rural/urban | Rural – 0, Urban – 1 | Rural – 0 |
| Livestock density index | Lower (0-21.4) – 0, Medium (21.5-73.2) – 1, Higher (73.3-5196.8) – 2 | Higher (73.3-5196.8) – 2 |
| Travel time to the nearest health facility (providing RI services) | Lower (0-3.9) – 0, Medium (4.0-12.5) – 1, Higher (12.6-532.8) – 2 | Higher (12.6-532.8) – 2 |
| Average enhanced vegetation index (2013-2018) | Lower (0.07-0.23) – 0, Medium (0.24-0.36) – 1, Higher (0.364-0.57) – 2 | Lower (0.07-0.23) |

**Geospatial covariate description and processing steps**

The geospatial covariates used in the analyses, as described in the main manuscript were travel time to the nearest health facility providing routine immunization (RI) services, livestock density and Enhanced Vegetation Index (EVI). These covariates were obtained from the sources mentioned in Table S2 and were processed as follows. Using ESRI ArcGIS v10.7, standardised gridded covariate layers were processed at 1km x 1 km for Nigeria from the raw data sets. For EVI, the data for the raster layers were taken from the 5-year period prior to the DHS survey, however, this was not possible for the other covariates. These layers were further processed to obtain the average EVI over the period. The livestock density index used in our work was obtained as the average of the individual livestock densities (i.e., cattle, chicken, goat, pig, and sheep) listed in Table B. Following these, covariate data values were extracted from each raster layer for each DHS cluster location using R. Prior to extraction, considerations for urban and rural clusters were made as in previous work [1] and buffers were created for data extraction to calculate a mean value at 2 km for urban areas and 5 km for rural areas. We present the surfaces of the resulting geospatial covariates and the extracted values at the cluster level in Fig A.

**Table B: Description and sources of geospatial covariates used in our analyses.**

| 1 | Cattle density | No. of cattle per sq km | 2010 | Continuous | Gilbert, M. *et al.*  (2018) Global Distribution Data for Cattle, Buffaloes, Horses, Sheep, Goats, Pigs, Chickens and Ducks in 2010. Nature Scientific data, 5:180227.[doi: 10.1038/sdata.2018.227](https://doi.org/10.1038/sdata.2018.227) |
| --- | --- | --- | --- | --- | --- |
| 2 | Chicken density | No. of chickens per sq km | 2010 | Continuous | Gilbert, M. *et al.*  (2018) Global Distribution Data for Cattle, Buffaloes, Horses, Sheep, Goats, Pigs, Chickens and Ducks in 2010. Nature Scientific data, 5:180227.[doi: 10.1038/sdata.2018.227](https://doi.org/10.1038/sdata.2018.227) |
| 3 | Goat density | No. of goats per sq km | 2010 | Continuous | Gilbert, M. *et al.*  (2018) Global Distribution Data for Cattle, Buffaloes, Horses, Sheep, Goats, Pigs, Chickens and Ducks in 2010. Nature Scientific data, 5:180227.[doi: 10.1038/sdata.2018.227](https://doi.org/10.1038/sdata.2018.227) |
| 4 | Pig density | No. Of pigs per sq km | 2010 | Continuous | Gilbert, M. *et al.*  (2018) Global Distribution Data for Cattle, Buffaloes, Horses, Sheep, Goats, Pigs, Chickens and Ducks in 2010. Nature Scientific data, 5:180227.[doi: 10.1038/sdata.2018.227](https://doi.org/10.1038/sdata.2018.227) |
| 5 | Sheep density | No. Of sheep per sq km | 2010 | Continuous | Gilbert, M. *et al.*  (2018) Global Distribution Data for Cattle, Buffaloes, Horses, Sheep, Goats, Pigs, Chickens and Ducks in 2010. Nature Scientific data, 5:180227.[doi: 10.1038/sdata.2018.227](https://doi.org/10.1038/sdata.2018.227) |
| 6 | Travel time to health facilities providing routine immunization (RI) | Minutes | 2018 | Continuous | [*Produced from locations of health facilities in Nigeria using the methodology in*] Weiss, D.J. *et al*. (2018). A global map of travel time to cities to access inequalities in accessibility in 2015. *Nature*. |
| 7 | Average Modis Enhanced Vegetation Index between 2013 and 2018 | EVI (0 to 1) | 2013-2018 | Continuous | Didan, K. (2015). MOD13A3 MODIS/Terra vegetation Indices Monthly L3 Global 1km SIN Grid V006. NASA EOSDIS LP DAAC. |


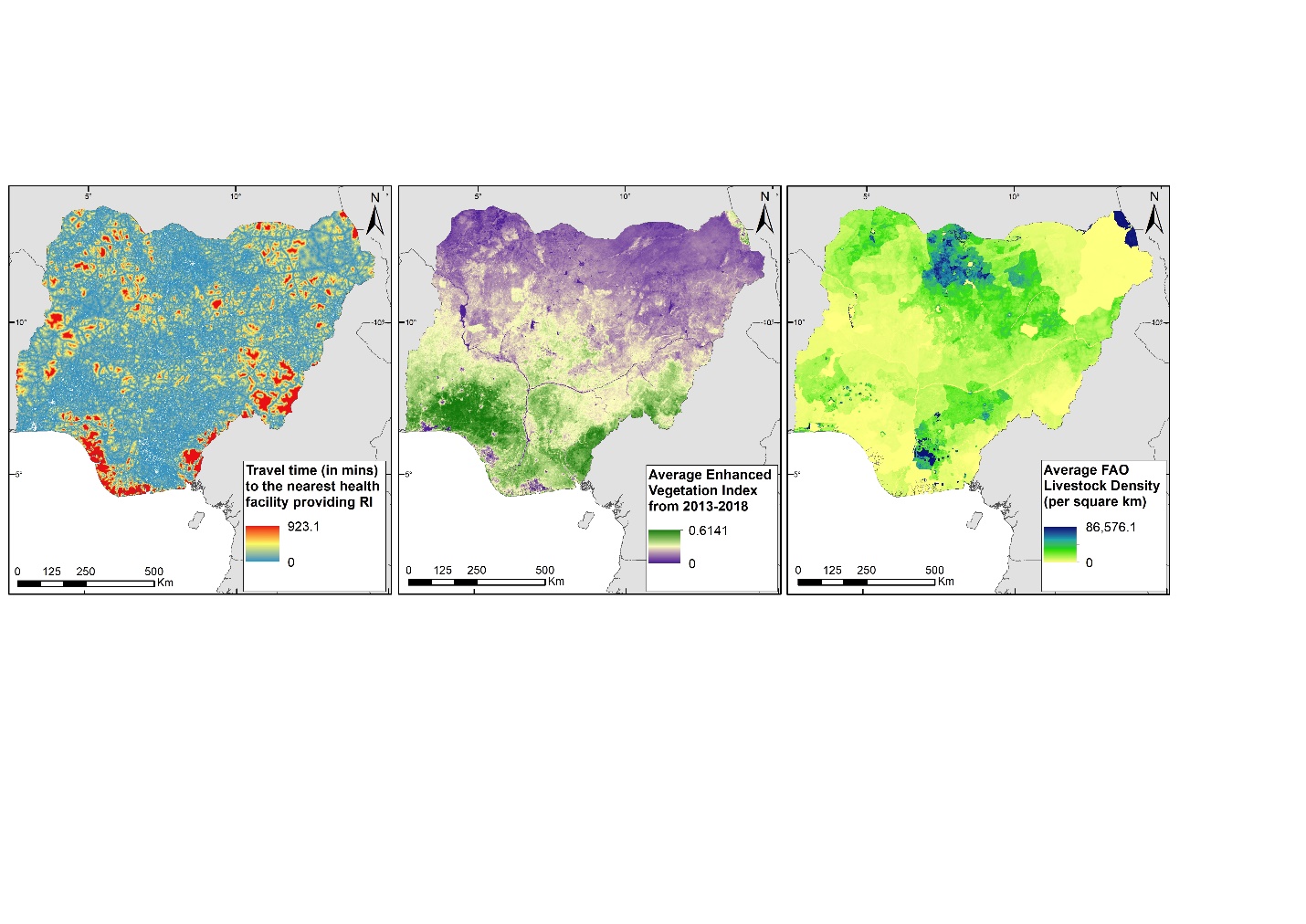


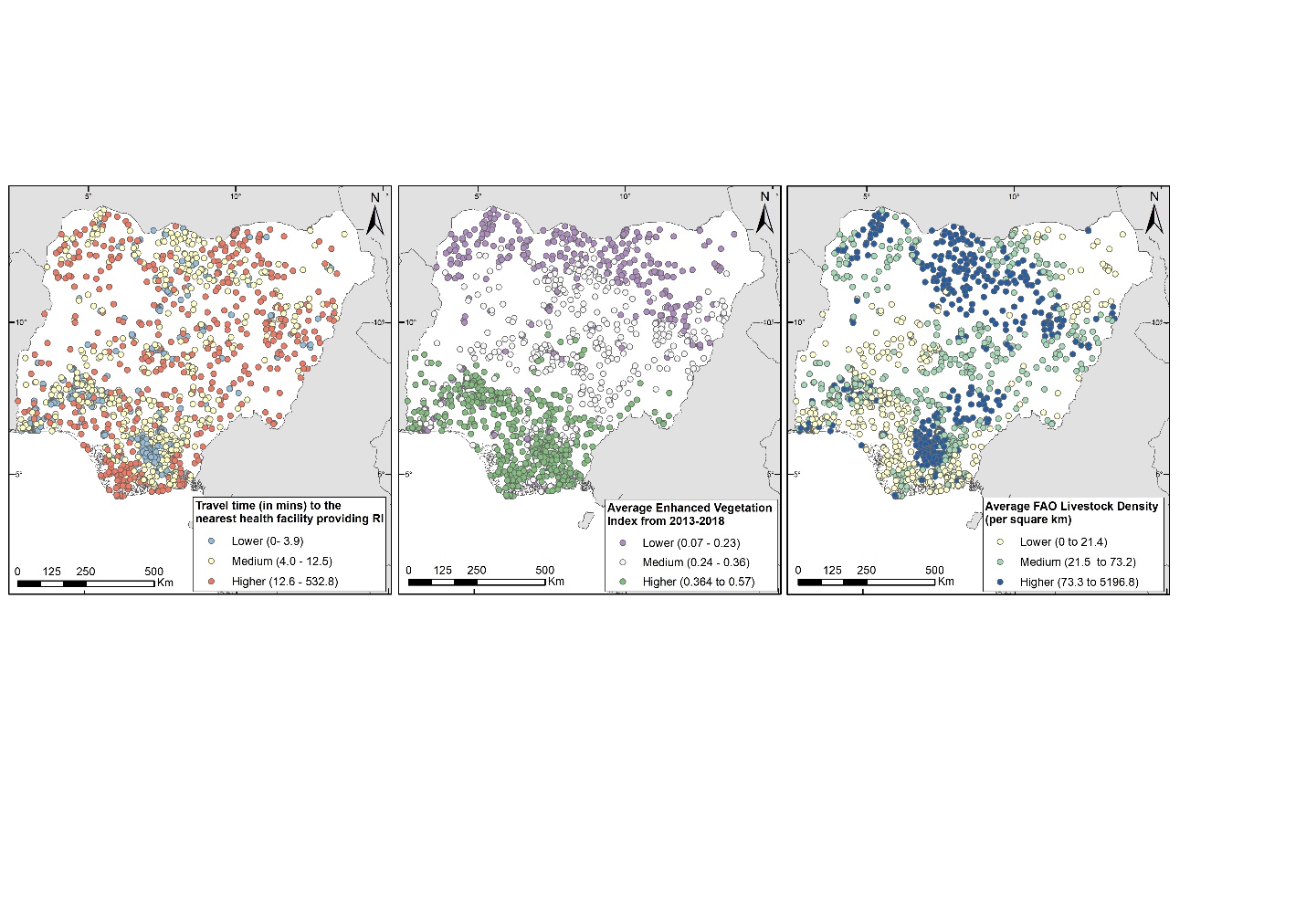


**Fig A. Geospatial covariates used in our analyses (top row) and the corresponding cluster-level values (bottom row). The three classes shown in the bottom row for each covariate were obtained using the tertiles of the distribution of the covariate.**

**Additional information on the data structure for our study**

Here, we provided the hierarchical structure of our data that necessitated the use of multilevel modelling framework in our study (Fig B).

Stratum 2

Stratum 1

Cluster 1

Cluster 2

Cluster 1

Cluster 2

Household 2

Household 1

Household 1

Household 1

Household 2

Household 1

child 1

child 2

child 1

child 2

child 1

child 2

child 1

child 1

child 2

child 1

**Fig B. The hierarchical structure of the data set used in the study.**

**Additional information on model-fitting and evaluation**

**Bayesian multiple multilevel binomial regression model**

We fitted Bayesian multilevel [2, 3] binomial regression models to examine factors that are predictive of each outcome. To account for the complex design used in the DHS (stratification and nesting within households and clusters/communities), we introduced a stratification variable and a cluster/community variable as random effects in our model. We did not include a household level random effect in our analysis due to insufficient sample sizes at this level and the resultant potential bias in variance estimates and effect sizes. To incorporate the predictors in our model, we first considered the selected DHS covariates and examined their contributions to the model fit as well as any problems due to multicollinearity. Where there was evidence of multicollinearity in all three indicators, the predictor was excluded from the analysis.

This was followed by the inclusion of the geospatial covariates. We excluded some of the pre-selected geospatial covariates from the analysis due to evidence of multicollinearity between these variables and some DHS covariates. The results of the fitted model were presented as adjusted odds ratios with their associated 95% Bayesian credible intervals. In addition, we presented the variance estimates, measures of variation attributable to the different levels in the data and some model evaluation statistics all of which are described below.

**Model building/specification**

The Bayesian multiple multilevel binomial regression is described as follows. Let $P_{ijk}$ be the probability that child *i* from community *j* in stratum $k$ received the given vaccination (coded as *yes* = 1 and *no* = 0 for any evidence of vaccination and no evidence of vaccination, respectively), and $\pi_{ijk}= \left( \frac{P_{ijk}}{1 - P_{ijk}} \right)$ the corresponding odds of vaccination. First, we considered an intercept only, three-level model (child, community/cluster and stratum levels) given by:

$\log\left( \frac{P_{ijk}}{1 - P_{ijk}} \right) = \beta_{0} + c_{0jk}+s_{0k}$ (1)

where $\beta_{0}$ is the intercept or overall probability of vaccination, $c_{0j}$ is the community-level random effect for the $j$th community, and $s_{0k}$ is the stratum-level random effect for stratum *k*. Further, $c_{0j}$ is assumed to follow a normal distribution with mean zero (0) and variance $\sigma_{c0}^{2}$, while $s_{0k}$ is also assumed to be normally distributed with zero mean and variance $\sigma_{s}^{2}$. This model was used to evaluate the contribution of covariates in the analyses.

We extended equation (1) to incorporate the set of covariates selected for the study. The full model can be expressed as:

$\log\left( \frac{P_{ijk}}{1 - P_{ijk}} \right) = \beta_{0} +\boldsymbol{d}\left( \boldsymbol{x}_{\boldsymbol{ijk}} \right)^{\boldsymbol{'}}\boldsymbol{\beta} + c_{0jk} + s_{0k}$ (2)

where $\boldsymbol{d}\left( . \right)$ is a vector of predictors that can be defined at the child, household, or community levels and $\boldsymbol{\beta}$ is the corresponding vector of regression coefficients. Other terms in the model are as defined previously. We note that in the model, the child-level (i.e., level-1) residual follows a standard logistic distribution with mean zero and variance $\frac{\pi^{2}}{3}$ which is approximately 3.29 [4, 5].

Furthermore, we fitted frequentist single level simple logistic regression models to obtain the corresponding crude odds ratios and associated 95% confidence intervals which were later compared with results from the multivariable analyses to determine changes in statistical significance and direction of effects.

The measures of variation considered in the study were: the variance partitioning coefficient (VPC), median odds ratio (MOR), and percentage change in variance (PCV) [6-10]. These are described as follows.

**Variance partitioning coefficient (VPC)**

We determined the VPC based on the estimated community and stratum variances from the fitted Bayesian multilevel models presented in equation (2). We estimated the VPC as:

VPC $=\left( \frac{\sigma_{c}^{2} + \sigma_{s}^{2}}{\sigma_{c}^{2} + \sigma_{s}^{2} + \frac{\pi^{2}}{3}} \right) \times100$ (4)

where $\sigma_{c}^{2}$ and $\sigma_{s}^{2}$ are the cluster and stratum level variances respectively, and $\frac{\pi^{2}}{3}$ is the child-level variance. The VPC measures the percentage of residual variation due to differences between communities in different strata.

**Median odds ratios (MOR) and Percentage change in variance (PCV)**

Similarly, the MOR and PCV were determined based on the estimated residual variances from the fitted model presented in equation (2). The MOR is the median odds ratio between a child in a community with a higher probability of receiving vaccination and a child in a community with a lower probability of receiving vaccination, given similar child level characteristics [11, 12]. Thus, the MOR quantifies residual community level variation (residual contextual heterogeneity) in the model on the odds ratio scale. It is easy to interpret and understand as it is expressed in terms of inter-community variance on the odds ratio scale, similar to the fixed effects [12]. We estimate the MOR based on community random effect as:

$$MOR = exp\left( \sqrt{2\times\sigma_{c}^{2}}\times\varphi^{-1}\left( 0.75 \right) \right) \cong exp\left( 0.945\times\sqrt{\sigma_{c}^{2}} \right) (5)$$

where $\sigma_{c}^{2}$ is the estimated community level variance from model (2) and $\varphi^{-1}\left( 0.75 \right)$ is the 75^th^ centile of the standard normal density which is approximately 0.6745.

The percentage change in variance (PCV) on the other hand is estimated as:

$$PCV =\left( \frac{V_{0} - V_{f}}{V_{0}} \right) \times100 (6)$$

where $V_{0}=\sigma_{c}^{2}+ \sigma_{s}^{2}$ is the variance from the empty model in equation (1) and $V_{f}$ is the corresponding variance from the full model in equation (2). The PCV measures the change in residual variance due to the inclusion of covariates in the fitted model.

**Bayesian multiple multilevel multinomial regression model**

Here, we extend equation (2) to allow for modelling of multinomial outcomes for PENTA1 and MV. For each outcome, the vaccination status is classified into three (3) categories: no evidence of vaccination = 0, card invalid/history vaccination = 1, and card valid vaccination = 2. Our interest is in estimating the likelihood of a child having either card invalid/history or card valid evidence of vaccination relative to no evidence of vaccination whilst adjusting for child, household, and community-level factors. Here again, we included stratification and cluster/community random effects as a way of adjusting for the complex survey design.

We assume that child *i* in community *j* within stratum *k* can be assigned to one of the three categories of vaccination and set the first category (i.e., no evidence of vaccination) to be the reference category. We set up a random intercept model incorporating the same predictors in the previous model in Equation (2). The model has two (2) equations contrasting the log-odds of having each of the evidence of vaccination: card invalid/history (1) and card valid (2) vaccinations against the reference category of no evidence of vaccination (0).

Let $Y_{ijk} \sim\mathrm{Multinomial}\left( \pi_{ijk}^{(1)}, \pi_{ijk}^{(2)} \right),$ where $Y_{ijk}$ is the vaccination status for child *i* in community *j* within stratum *k*, and $\pi_{ijk}^{(1)} \mathrm{and}\pi_{ijk}^{(2)}$ are the probabilities of having card invalid/history and card valid evidence of vaccination, respectively.

The multinomial multilevel model[13, 14] is given as follows:

$\log\left( \frac{\pi_{ijk}^{(1)}}{\pi_{ijk}^{(0)}} \right) = \alpha_{0}^{(1)} + \boldsymbol{d}{(\boldsymbol{x}_{ijk})}^{'}\boldsymbol{\beta}^{(1)} + c_{jk}^{(1)} + s_{k}^{(1)}$,

$\log\left( \frac{\pi_{ijk}^{(2)}}{\pi_{ijk}^{(0)}} \right) = \alpha_{0}^{(2)} + \boldsymbol{d}{(\boldsymbol{x}_{ijk})}^{'}\boldsymbol{\beta}^{(2)} + c_{jk}^{(2)} + s_{k}^{(2)}$,

$\left( \begin{matrix} c_{jk}^{(1)} \\ c_{jk}^{(2)} \end{matrix} \right) \sim MVN\left\{ \left( \begin{matrix} 0 \\ 0 \end{matrix} \right) \left( \begin{matrix} \sigma_{c(1)}^{2} & \\ \sigma_{c(1,2)}^{2} & \sigma_{c(2)}^{2} \end{matrix} \right) \right\}$,

$\left( \begin{matrix} s_{k}^{(1)} \\ s_{k}^{(2)} \end{matrix} \right) \sim MVN\left\{ \left( \begin{matrix} 0 \\ 0 \end{matrix} \right) \left( \begin{matrix} \sigma_{s(1)}^{2} & \\ \sigma_{s(1,2)}^{2} & \sigma_{s(2)}^{2} \end{matrix} \right) \right\}, (3)$

where, $\alpha_{0}^{(1)}$ and $\alpha_{0}^{(2)}$ are the intercepts for card invalid/history and card valid evidence of vaccination, respectively. $\boldsymbol{d}\left( . \right)$ is a vector of predictors, and $\boldsymbol{\beta}^{(1)}$ and $\boldsymbol{\beta}^{(2)}$ are the corresponding vectors of regression coefficients. The quantities $c_{jk}^{(1)}$ and $c_{jk}^{(2)}$, and $s_{k}^{(1)}$ and $s_{k}^{(2)}$ are assumed to follow multivariate normal distributions. $\sigma_{c(1)}^{2}$ and $\sigma_{c(2)}^{2}$ are the community-level variances for card invalid/history and card valid evidence of vaccination, respectively, and their corresponding covariance $\sigma_{c(1,2)}^{2}$, while $\sigma_{s(1)}^{2}$ and $\sigma_{s(2)}^{2}$ are the corresponding stratum-level variances, and their corresponding covariance $\sigma_{s(1,2)}^{2}$.

**Prior distributions for fixed and random effects**

We complete our Bayesian specification by placing appropriate prior distributions on the parameters of the models. We specified Gaussian priors for the fixed effect parameters, including the intercept, and Gamma priors for all the variance parameters in both the binomial and multinomial models. Further, we obtained the initial values of all the parameters, as well as the hyperparameters, from frequentist analyses for each indicator and covariate using corresponding multilevel models and the same set of covariates included in the final Bayesian analyses, except the multinomial model for PENTA1 where we set the means of the Gaussian priors of some fixed effect parameters to log(1) due to non-convergence problems. We deemed this approach reasonable due to lack of information on reliable priors for our model parameters [15]. An alternative approach is to use non-informative priors for the model parameters.

**Bayesian models**

One of our interests in this study is to employ novel Bayesian modelling approach to investigate predictors of childhood vaccination. Undoubtedly, the Bayesian modelling is one of the novel approaches in model fitting and model diagnostics. For example, in the Bayesian approach, two different sources of uncertainties (i.e., uncertainty in the parameter values and sampling uncertainty) in our estimates can be quantified and the 95% credible intervals fixed while the estimated parameters are allowed to vary unlike the frequentist (i.e., classical) approach. Also, the type of the modelling implemented in this study, especially the multiple multilevel multinomial and binomial regression models can effectively and efficiently be implemented in the Bayesian framework compared to the frequentist due to the model complexity and computational cost. These are the key reasons we opted for the Bayesian approach rather than the frequentist.

**Multicollinearity assessment**

We addressed the problem of (multi)collinearity by computing the generalized variance inflation factors (GVIFs) [16] of the covariates for each country-vaccine combination and excluded variables that had high GVIFs (> 2, on the scale that ensures comparability across the covariates as recommended by Fox and Monette, 1992) [16-18].

**Additional results of the binomial analysis for PENTA1**

**Table C: Factors associated with any evidence of PENTA1 vaccination, cross-tabulation analyses of Nigeria DHS 2018**

|  |  | **PENTA1** | |  |
| --- | --- | --- | --- | --- |
|  |  | **No** | **Yes** |  |
| **Variables** | **N** | **n (%)** | **n (%)** | **P-value** |
| **N** | 6059 | 2122 (35) | 3937 (65) |  |
| **Sex of child** |  |  |  | 0.690 |
| Male | 3148 | 1110 (35.3) | 2038 (64.7) |  |
| Female | 2911 | 1012 (34.8) | 1899 (65.2) |  |
| **Birth order** |  |  |  | <0.001*** |
| 1-2 | 2279 | 674 (29.6) | 1605 (70.4) |  |
| >2 | 3780 | 1448 (38.3) | 2332 (61.7) |  |
| **Birth quarter** |  |  |  | 0.052 |
| Jan-Mar | 1909 | 630 (33.0) | 1279 (67.0) |  |
| Apr-Jun | 1668 | 592 (35.5) | 1076 (64.5) |  |
| Jul-Sep | 1628 | 608 (37.3) | 1020 (62.7) |  |
| Oct-Dec | 854 | 292 (34.2) | 562 (65.8) |  |
| **Skilled birth attendance** |  |  |  | <0.001*** |
| No skilled attendant at birth | 3328 | 1772 (53.2) | 1556 (46.8) |  |
| Skilled attendant at birth | 2731 | 350 (12.8) | 2381 (87.2) |  |
| **Health card/document** |  |  |  | <0.001*** |
| Does not have health card/document | 3113 | 1934 (62.1) | 1179 (37.9) |  |
| Yes, none seen | 519 | 42 (8.1) | 477 (91.9) |  |
| Yes, seen | 2427 | 146 (6.0) | 2281 (94.0) |  |
| **Received vitamin A** |  |  |  | <0.001*** |
| No/don’t know | 2852 | 1569 (55.0) | 1283 (45.0) |  |
| Yes | 3207 | 553 (17.2) | 2654 (82.8) |  |
| **Sex of household head** |  |  |  | <0.001*** |
| Male | 5434 | 1996 (36.7) | 3438 (63.3) |  |
| Female | 625 | 126 (20.2) | 499 (79.8) |  |
| **Mother's age group** |  |  |  | <0.001*** |
| 15-19 | 366 | 183 (50.0) | 183 (50.0) |  |
| 20-29 | 3019 | 1054 (34.9) | 1965 (65.1) |  |
| 30-39 | 2237 | 684 (30.6) | 1553 (69.4) |  |
| 40-49 | 437 | 201 (46.0) | 236 (54.0) |  |
| **Marital status of mother** |  |  |  | 0.002 |
| Never in union | 171 | 38 (22.2) | 133 (77.8) |  |
| Married | 5730 | 2029 (35.4) | 3701 (64.6) |  |
| Divorced | 158 | 55 (34.8) | 103 (65.2) |  |
| **Mother employed in the past 12 months** |  |  |  | <0.001*** |
| No | 1777 | 854 (48.1) | 923 (51.9) |  |
| Yes (currently/in the past 1 year) | 4282 | 1268 (29.6) | 3014 (70.4) |  |
| **Mother had problem seeking medical advice or treatment** |  |  |  | <0.001*** |
| Had problem seeking medical advice or treatment | 3397 | 1387 (40.8) | 2010 (59.2) |  |
| Did not have problem seeking medical advice or treatment | 2662 | 735 (27.6) | 1927 (72.4) |  |
| **Mother’s education** |  |  |  | <0.001*** |
| No education | 2614 | 1567 (59.9) | 1047 (40.1) |  |
| Primary | 881 | 242 (27.5) | 639 (72.5) |  |
| Secondary/higher | 2564 | 313 (12.2) | 2251 (87.8) |  |
| **Mother’s religion** |  |  |  | <0.001*** |
| Islam | 3538 | 1756 (49.6) | 1782 (50.4) |  |
| Christian | 2469 | 343 (13.9) | 2126 (86.1) |  |
| Traditionalist/others | 52 | 23 (44.2) | 29 (55.8) |  |
| **Mother’s media exposure** |  |  |  | <0.001*** |
| No | 3729 | 1652 (44.3) | 2077 (55.7) |  |
| Yes (radio/tv/newspaper at least once a week) | 2330 | 470 (20.2) | 1860 (79.8) |  |
| **Mother’s access to mobile phone/internet** |  |  |  | <0.001*** |
| No | 3062 | 1562 (51.0) | 1500 (49.0) |  |
| Yes | 2997 | 560 (18.7) | 2437 (81.3) |  |
| **Mother’s land ownership** |  |  |  | <0.001*** |
| Does not own land | 5236 | 1927 (36.8) | 3309 (63.2) |  |
| Owns land alone and/or jointly | 823 | 195 (23.7) | 628 (76.3) |  |
| **Mother’s knowledge of malaria** |  |  |  | 0.100 |
| Has no knowledge | 386 | 150 (38.9) | 236 (61.1) |  |
| Has knowledge | 5673 | 1972 (34.8) | 3701 (65.2) |  |
| **Mother had health insurance** |  |  |  | <0.001*** |
| No | 5925 | 2102 (35.5) | 3823 (64.5) |  |
| Yes | 134 | 20 (14.9) | 114 (85.1) |  |
| **Household’s bed net ownership** |  |  |  | <0.001*** |
| No | 1780 | 516 (29.0) | 1264 (71.0) |  |
| Yes | 4279 | 1606 (37.5) | 2673 (62.5) |  |
| **Mother’s ethnicity** |  |  |  | <0.001*** |
| Hausa/Fulani | 2364 | 1354 (57.3) | 1010 (42.7) |  |
| Yoruba | 622 | 86 (13.8) | 536 (86.2) |  |
| Igbo | 847 | 60 (7.1) | 787 (92.9) |  |
| Others (ekoi, ibibio, etc.) | 2226 | 622 (27.9) | 1604 (72.1) |  |
| **Household wealth** |  |  |  | <0.001*** |
| Poorer/poorest | 2784 | 1486 (53.4) | 1298 (46.6) |  |
| Middle | 1253 | 366 (29.2) | 887 (70.8) |  |
| Richer/richest | 2022 | 270 (13.4) | 1752 (86.6) |  |
| **Access to bank account** |  |  |  | <0.001*** |
| No | 5035 | 2063 (41.0) | 2972 (59.0) |  |
| Yes | 1024 | 59 (5.8) | 965 (94.2) |  |
| **Household size** |  |  |  | <0.001*** |
| Large (>=9) | 1464 | 705 (48.2) | 759 (51.8) |  |
| Medium (5 to 8) | 2760 | 906 (32.8) | 1854 (67.2) |  |
| Small (<=4) | 1835 | 511 (27.8) | 1324 (72.2) |  |
| **Length of stay in household** |  |  |  | <0.001*** |
| <1year/visitor | 174 | 27 (15.5) | 147 (84.5) |  |
| 1-3years | 711 | 177 (24.9) | 534 (75.1) |  |
| 4-5years | 438 | 121 (27.6) | 317 (72.4) |  |
| >5years/always | 4736 | 1797 (37.9) | 2939 (62.1) |  |
| **Rural/urban** |  |  |  | <0.001*** |
| Rural | 3959 | 1717 (43.4) | 2242 (56.6) |  |
| Urban | 2100 | 405 (19.3) | 1695 (80.7) |  |
| **Livestock density index** |  |  |  | <0.001*** |
| Lower (0-21.4) | 1637 | 434 (26.5) | 1203 (73.5) |  |
| Medium (21.5-73.2) | 2034 | 702 (34.5) | 1332 (65.5) |  |
| Higher (73.3-5196.8) | 2365 | 981 (41.5) | 1384 (58.5) |  |
| **Travel time to the nearest health facility (providing RI services)** |  |  |  | <0.001*** |
| Lower (0-3.9) | 1683 | 288 (17.1) | 1395 (82.9) |  |
| Medium (4.0-12.5) | 2000 | 661 (33.1) | 1339 (67.0) |  |
| Higher (12.6-532.8) | 2353 | 1168 (49.6) | 1185 (50.4) |  |
| **Average enhanced vegetation index (2013-2018)** |  |  |  | <0.001*** |
| Lower (0.07-0.23) | 2395 | 1184 (49.4) | 1211 (50.6) |  |
| Medium (0.24-0.36) | 2202 | 688 (31.2) | 1514 (68.8) |  |
| Higher (0.364-0.57) | 1439 | 245 (17.0) | 1194 (83.0) |  |

*p<0.05; **p<0.01; ***p<0.001

**Table D: Percentage of children aged 12 to 23 months with any evidence of PENTA1 vaccination, according to potential determinants of vaccination, univariate and multivariable analyses of Nigeria DHS 2018**

| **Characteristics and categories** | **Number (%) in category** | **Percentage with any evidence of PENTA1 [95% CI]** | **Frequentist single-**  **level model:**  **cOR [95% CI]** | **Bayesian multilevel model:**  **aOR [95% Cr. I]** |
| --- | --- | --- | --- | --- |
| **N** | 6059(100.0) |  |  |  |
| **Sex of child** |  |  |  |  |
| Male | 3148 (52.0) | 64.7 [63.1, 66.4] | 1.00 [reference] | 1.00 [reference] |
| Female | 2911 (48.0) | 65.2 [63.5, 66.9] | 1.02 [0.92, 1.14] | 0.97 [0.81, 1.15] |
| **Birth order** |  |  |  |  |
| 1-2 | 2279 (37.6) | 70.4 [68.5, 72.3] | 1.00 [reference] | 1.00 [reference] |
| >2 | 3780 (62.4) | 61.7 [60.1, 63.2] | 0.68 [0.61, 0.76] *** | 1.01 [0.77, 1.30] |
| **Birth quarter** |  |  |  |  |
| Jan-Mar | 1909 (31.5) | 67.0 [64.9, 69.1] | 1.05 [0.89, 1.25] | 1.28 [0.96, 1.68] |
| Apr-Jun | 1668 (27.5) | 64.5 [62.2, 66.8] | 0.94 [0.79, 1.12] | 1.25 [0.93, 1.65] |
| Jul-Sep | 1628 (26.9) | 62.7 [60.3, 65.0] | 0.87 [0.73, 1.04] | 1.21 [0.90, 1.60] |
| Oct-Dec | 854 (14.1) | 65.8 [62.6, 68.9] | 1.00 [reference] | 1.00 [reference] |
| **Skilled birth attendance** |  |  |  |  |
| No skilled attendant at birth | 3328 (54.9) | 46.8 [45.1, 48.5] | 1.00 [reference] | 1.00 [reference] |
| Skilled attendant at birth | 2731 (45.1) | 87.2 [85.9, 88.4] | 7.75 [6.79, 8.83] *** | 2.00 [1.56, 2.53] ^+^ |
| **Health card/document** |  |  |  |  |
| Does not have health card/document | 3113 (51.4) | 37.9 [36.2, 39.6] | 1.00 [reference] | 1.00 [reference] |
| Yes, none seen | 519 (8.6) | 91.9 [89.2, 94.0] | 18.63 [13.48, 25.75] *** | 14.34 [9.25, 21.66] ^+^ |
| Yes, seen | 2427 (40.1) | 94.0 [93.0, 94.9] | 25.63 [21.36, 30.75] *** | 24.36 [19.02, 31.25] ^+^ |
| **Received vitamin A** |  |  |  |  |
| No/don’t know | 2852 (47.1) | 45.0 [43.2, 46.8] | 1.00 [reference] | 1.00 [reference] |
| Yes | 3207 (52.9) | 82.8 [81.4, 84.0] | 5.87 [5.22, 6.60] *** | 4.08 [3.29, 5.00] ^+^ |
| **Sex of household head** |  |  |  |  |
| Male | 5434 (89.7) | 63.3 [62.0, 64.5] | 1.00 [reference] | 1.00 [reference] |
| Female | 625 (10.3) | 79.8 [76.5, 82.8] | 2.30 [1.88, 2.82] *** | 1.18 [0.84, 1.62] |
| **Mother's age group** |  |  |  |  |
| 15-19 | 366 (6.0) | 50.0 [44.9, 55.1] | 1.00 [reference] | 1.00 [reference] |
| 20-29 | 3019 (49.8) | 65.1 [63.4, 66.8] | 1.86 [1.50, 2.32] *** | 1.30 [0.86, 1.84] |
| 30-39 | 2237 (36.9) | 69.4 [67.5, 71.3] | 2.27 [1.82, 2.84] *** | 1.24 [0.78, 1.86] |
| 40-49 | 437 (7.2) | 54.0 [49.3, 58.6] | 1.17 [0.89, 1.55] | 0.84 [0.48, 1.37] |
| **Marital status of mother** |  |  |  |  |
| Never in union | 171 (2.8) | 77.8 [70.9, 83.4] | 1.92 [1.33, 2.76] *** | 0.78 [0.41, 1.35] |
| Married | 5730 (94.6) | 64.6 [63.3, 65.8] | 1.00 [reference] | 1.00 [reference] |
| Divorced | 158 (2.6) | 65.2 [57.4, 72.2] | 1.03 [0.74, 1.43] | 0.76 [0.43, 1.29] |
| **Mother employed in the past 12 months** |  |  |  |  |
| No | 1777 (29.3) | 51.9 [49.6, 54.3] | 1.00 [reference] | 1.00 [reference] |
| Yes (currently/in the past 1 year) | 4282 (70.7) | 70.4 [69.0, 71.7] | 2.20 [1.96, 2.46] *** | 1.30 [1.06, 1.59] ^+^ |
| **Mother had problem seeking medical advice or treatment** |  |  |  |  |
| Did not have problem seeking medical advice or treatment | 3397 (56.1) | 59.2 [57.5, 60.8] | 1.00 [reference] | 1.00 [reference] |
| Had problem seeking medical advice or treatment | 2662 (43.9) | 72.4 [70.7, 74.1] | 1.81 [1.62, 2.02] *** | 1.29 [1.06, 1.57] ^+^ |
| **Mother’s religion** |  |  |  |  |
| Islam | 3538 (58.4) | 50.4 [48.7, 52.0] | 1.00 [reference] | 1.00 [reference] |
| Christian | 2469 (40.7) | 86.1 [84.7, 87.4] | 6.11 [5.35, 6.97] *** | 1.53 [1.08, 2.10] ^+^ |
| Traditionalist/others | 52 (0.9) | 55.8 [42.2, 68.5] | 1.24 [0.72, 2.16] | 0.92 [0.32, 2.20] |
| **Mother’s media exposure** |  |  |  |  |
| No | 3729 (61.5) | 55.7 [54.1, 57.3] | 1.00 [reference] | 1.00 [reference] |
| Yes (radio/tv/newspaper at least once a week) | 2330 (38.5) | 79.8 [78.1, 81.4] | 3.15 [2.79, 3.55] *** | 0.99 [0.79, 1.21] |
| **Mother’s access to mobile phone/internet** |  |  |  |  |
| No | 3062 (50.5) | 49.0 [47.2, 50.8] | 1.00 [reference] | 1.00 [reference] |
| Yes | 2997 (49.5) | 81.3 [79.9, 82.7] | 4.53 [4.04, 5.09] *** | 1.25 [1.01, 1.56] ^+^ |
| **Mother’s land ownership** |  |  |  |  |
| Does not own land | 5236 (86.4) | 63.2 [61.9, 64.5] | 1.00 [reference] | 1.00 [reference] |
| Owns land alone and/or jointly | 823 (13.6) | 76.3 [73.3, 79.1] | 1.88 [1.58, 2.22] *** | 1.18 [0.87, 1.56] |
| **Mother’s knowledge of malaria** |  |  |  |  |
| Has no knowledge | 386 (6.4) | 61.1 [56.2, 65.9] | 1.00 [reference] | 1.00 [reference] |
| Has knowledge | 5673 (93.6) | 65.2 [64.0, 66.5] | 1.19 [0.97, 1.47] | 1.23 [0.84, 1.75] |
| **Mother had health insurance** |  |  |  |  |
| No | 5925 (97.8) | 64.5 [63.3, 65.7] | 1.00 [reference] | 1.00 [reference] |
| Yes | 134 (2.2) | 85.1 [78.0, 90.2] | 3.13 [1.94, 5.06] *** | 2.08 [0.89, 4.26] |
| **Household’s bed net ownership** |  |  |  |  |
| No | 1780 (29.4) | 71.0 [68.9, 73.1] | 1.00 [reference] | 1.00 [reference] |
| Yes | 4279 (70.6) | 62.5 [61.0, 63.9] | 0.68 [0.60, 0.77] *** | 0.97 [0.78, 1.19] |
| **Mother’s ethnicity** |  |  |  |  |
| Hausa/Fulani | 2364 (39.0) | 42.7 [40.7, 44.7] | 1.00 [reference] | 1.00 [reference] |
| Yoruba | 622 (10.3) | 86.2 [83.2, 88.7] | 8.36 [6.56, 10.64] *** | 1.24 [0.69, 2.05] |
| Igbo | 847 (14.0) | 92.9 [91.0, 94.5] | 17.58 [13.36, 23.15] *** | 1.98 [1.01, 3.54] ^+^ |
| Others (ekoi, ibibio, etc.) | 2226 (36.7) | 72.1 [70.2, 73.9] | 3.46 [3.06, 3.91] *** | 1.30 [0.95, 1.75] |
| **Household wealth** |  |  |  |  |
| Poorer/poorest | 2784 (45.9) | 46.6 [44.8, 48.5] | 1.00 [reference] | 1.00 [reference] |
| Middle | 1253 (20.7) | 70.8 [68.2, 73.2] | 2.77 [2.41, 3.20] *** | 1.07 [0.82, 1.37] |
| Richer/richest | 2022 (33.4) | 86.6 [85.1, 88.1] | 7.43 [6.41, 8.62] *** | 1.32 [0.95, 1.80] |
| **Access to bank account** |  |  |  |  |
| No | 5035 (83.1) | 59.0 [57.7, 60.4] | 1.00 [reference] | 1.00 [reference] |
| Yes | 1024 (16.9) | 94.2 [92.6, 95.5] | 11.35 [8.68, 14.85] *** | 2.69 [1.78, 3.94] ^+^ |
| **Household size** |  |  |  |  |
| Large (>=9) | 1464 (24.2) | 51.8 [49.3, 54.4] | 0.53 [0.46, 0.60] *** | 1.10 [0.87, 1.36] |
| Medium (5 to 8) | 2760 (45.6) | 67.2 [65.4, 68.9] | 1.00 [reference] | 1.00 [reference] |
| Small (<=4) | 1835 (30.3) | 72.2 [70.1, 74.2] | 1.27 [1.11, 1.44] *** | 1.13 [0.87, 1.45] |
| **Length of stay in household** |  |  |  |  |
| <1year/visitor | 174 (2.9) | 84.5 [78.3, 89.1] | 2.08 [1.31, 3.29] ** | 2.32 [1.10, 4.29] ^+^ |
| 1-3years | 711 (11.7) | 75.1 [71.8, 78.1] | 1.15 [0.88, 1.51] | 1.08 [0.66, 1.67] |
| 4-5years | 438 (7.2) | 72.4 [68.0, 76.4] | 1.00 [reference] | 1.00 [reference] |
| >5years/always | 4736 (78.2) | 62.1 [60.7, 63.4] | 0.62 [0.50, 0.78] *** | 1.53 [1.05, 2.17] ^+^ |
| **Rural/urban** |  |  |  |  |
| Rural | 3959 (65.3) | 56.6 [55.1, 58.2] | 1.00 [reference] | 1.00 [reference] |
| Urban | 2100 (34.7) | 80.7 [79.0, 82.3] | 3.21 [2.83, 3.63] *** | 0.94 [0.57, 1.46] |
| **Livestock density index** |  |  |  |  |
| Lower (0-21.4) | 1637 (27.0) | 73.5 [71.3, 75.6] | 1.96 [1.71, 2.25] *** | 1.66 [1.13, 2.37] ^+^ |
| Medium (21.5-73.2) | 2034 (33.6) | 65.5 [63.4, 67.5] | 1.34 [1.19, 1.52] *** | 1.25 [0.90, 1.72] |
| Higher (73.3-5196.8) | 2365 (39.0) | 58.5 [56.5, 60.5] | 1.00 [reference] | 1.00 [reference] |
| **Average enhanced vegetation index (2013-2018)** |  |  |  |  |
| Lower (0.07-0.23) | 2395 (39.5) | 50.6 [48.6, 52.6] | 1.00 [reference] | 1.00 [reference] |
| Medium (0.24-0.36) | 2202 (36.3) | 68.8 [66.8, 70.7] | 2.15 [1.91, 2.43] *** | 1.45 [1.07, 1.93] ^+^ |
| Higher (0.364-0.57) | 1439 (23.7) | 83.0 [80.9, 84.8] | 4.76 [4.06, 5.59] *** | 1.26 [0.80, 1.89] |
|  | | | | |
| **Variance parameters** |  |  |  | **Estimate [95% Cr. I]** |
| Stratum-level variance |  |  |  | 0.39 [0.19, 0.67] |
| Cluster-level variance |  |  |  | 0.74 [0.45, 1.05] |

CI: Confidence Interval; Cr. I: Bayesian Credible Interval; cOR: Crude Odds Ratio; aOR: adjusted Odds Ratio; ^+^Significant covariate for the Bayesian model; *p<0.05; **p<0.01; ***p<0.001.

**Additional information on the results of the analyses**

**Derivation of the odds ratios for the interaction between maternal education and travel time to the nearest health facility in the binomial analysis for PENTA1**

We found a significant interaction between maternal education and travel time to the nearest health facility in the study for PENTA1. To appropriately interpret this significant interaction, the correct odds ratios need to be computed as demonstrated by Chen [19]. These are given as follows using parameter estimates reported in Table E.

For children born to mothers with primary education (compared to no education), the odds ratio (OR) for those residing in communities with lower travel time to the nearest health facility is given as:

$\hat{\mathrm{OR}}$ for primary education versus no education $= exp(\hat{\beta}_{primary}+\hat{\beta}_{(primary and lower travel time})$

$=\hat{OR}_{primary} \times\hat{OR}_{(primary and lower travel time})$

$=1.89 \times0.53 = 1.00$for lower travel time.

Also, for children born to mothers with primary education (compared to no education), the odds ratio (OR) for those residing in communities with medium travel time to the nearest health facility is given as:

$\hat{\mathrm{OR}}$ for primary education versus no education $=exp(\hat{\beta}_{primary}+ \hat{\beta}_{(primary and medium travel time})$

$=\hat{OR}_{primary} \times\hat{OR}_{(primary and medium travel time})$

$=1.89 \times0.63 = 1.19$for medium travel time.

Lastly, for children born to mothers with primary education (compared to no education), the odds ratio (OR) for those residing in communities with higher travel time to the nearest health facility is given as:

$\hat{\mathrm{OR}}$ for primary education versus no education $= exp(\hat{\beta}_{primary})$

$=\hat{OR}_{primary}$

$=1.89$for higher travel time.

Next, for children born to mothers with secondary/higher education (compared to no education), the odds ratios can be calculated similarly as follows:

$\hat{\mathrm{OR}}$ for secondary/higher education vs no education = 2.59 × 0.43 = 1.11 for lower travel time.

= 2.59 × 0.82 = 2.12 for medium travel time.

= 2.59 for higher travel time.

The corresponding 95% credible intervals for these calculations were estimated using the posterior samples of the relevant parameters in each case, using the *lincom* utility in Stata. These results are also presented in Table E.

Our interpretations are based on the above calculations. However, travel time could also serve as the base variable in these calculations as follows:

$\hat{\mathrm{OR}}$ for lower travel time versus higher travel time = 2.27 for no education.

= 2.27 × 0.53 = 1.20 for primary education.

= 2.27 × 0.43 = 0.98 for secondary/higher education.

$\hat{\mathrm{OR}}$ for medium travel time vs higher travel time = 1.64 for no education.

= 1.64 × 0.63 = 1.03 for primary education.

= 2.27 × 0.82 = 1.86 for secondary/higher education.

The corresponding 95% credible intervals for these estimates were also estimated as discussed above (see Table E).

**Table E: Maternal education and travel time and the interaction between both variables.**

| **Characteristics and categories** | **Number (%) in category** | **Percentage with any evidence of PENTA1 (95% CI)** | **Frequentist single-**  **level model:**  **cOR [95% CI** | **Bayesian multilevel model:**  **aOR [95% Cr. I]** |
| --- | --- | --- | --- | --- |
| **Mother’s education** |  |  |  |  |
| No education | 2614 (43.1) | 40.1 [38.2, 41.9] | 1.00 [reference] | 1.00 [reference] |
| Primary | 881 (14.5) | 72.5 [69.5, 75.4] | 3.95 [3.34, 4.67] *** | 1.89 [1.24, 2.75] ^+^ |
| Secondary/higher | 2564 (42.3) | 87.8 [86.5, 89.0] | 10.76 [9.34, 12.40] *** | 2.59 [1.71, 3.87] ^+^ |
| **Travel time to the nearest health facility (providing RI services)** |  |  |  |  |
| Lower (0-3.9) | 1683 (27.8) | 82.9 [81.0, 84.6] | 4.83 [4.16, 5.62] *** | 2.27 [1.17, 3.97] ^+^ |
| Medium (4.0-12.5) | 2000 (33.0) | 67.0 [64.9, 69.0] | 2.02 [1.78, 2.28] *** | 1.64 [1.15, 2.30] ^+^ |
| Higher (12.6-532.8) | 2353 (38.8) | 50.4 [48.3, 52.4] | 1.00 [reference] | 1.00 [reference] |
| **Interaction between education and travel time** | Number in category | **Number vaccinated** (%) |  |  |
| Primary education and lower travel time | 215 | 170 (79.1) | - | 0.53 [0.24, 1.04] |
| Secondary/higher education and lower travel time | 1192 | 1077 (90.4) | - | 0.43 [0.21, 0.76] ^+^ |
| Primary education and medium travel time | 326 | 232 (71.2) | - | 0.63 [0.35, 1.07] |
| Secondary/higher education and medium travel time | 799 | 713 (89.2) | - | 0.82 [0.46, 1.34] |
| Primary education and higher travel time | 338 | 232 (68.6) | - | - |
| Secondary/higher education and higher travel time | 573 | 458 (79.9) | - | - |
| **Calculated odds ratios (ORs) for the interaction effects** | | | | |
| **Education** | **Travel time to the nearest health facility** | | | |
|  | Higher (OR) | Medium (OR) | Lower (OR) | |
| Primary versus no education | 1.89 [1.25, 2.76] | 1.19 [0.73, 1.74] | 1.00 [0.49, 1.73] | |
| Secondary/higher versus no education | 2.59 [1.68, 3.82] | 2.12 [1.30, 3.10] | 1.11 [0.61, 1.73] | |
| **Travel time to the nearest health facility** | **Education** | | | |
|  | Secondary/  Higher (OR) | Primary (OR) | No education | |
| Lower versus higher travel time | 0.98 [0.50, 1.54] | 1.20 [0.54, 2.16] | 2.27 [1.18, 3.98] | |
| Medium versus higher travel time | 1.86 [0.81, 2.04] | 1.03 [0.58, 1.65] | 1.64 [1.15, 2.27] | |

CI: Confidence Interval; Cr. I: Bayesian Credible Interval; cOR: Crude Odds Ratio; aOR: adjusted Odds Ratio; ^+^Significant covariate for the Bayesian _model;_ *p<0.05; **p<0.01; ***p<0.001.

**Additional results of the binomial analysis for PENTA3/1**

**Table F: Factors associated with receipt of PENTA3/1 vaccination, cross-tabulation analyses of Nigeria DHS 2018**

|  |  | **PENTA3/1** | |  |
| --- | --- | --- | --- | --- |
|  |  | **Received PENTA1 but not PENTA3** | **Received both** | **P-value** |
|  | **N** | **n (%)** | **n (%)** |  |
| **N** |  | 896 (23) | 3041 (77) |  |
| **Sex of child** |  |  |  | 0.990 |
| Male | 2038 | 464 (22.8) | 1574 (77.2) |  |
| Female | 1899 | 432 (22.7) | 1467 (77.3) |  |
| **Birth order** |  |  |  | 0.008 |
| 1-2 | 1605 | 331 (20.6) | 1274 (79.4) |  |
| >2 | 2332 | 565 (24.2) | 1767 (75.8) |  |
| **Birth quarter** |  |  |  | 0.210 |
| Jan-Mar | 1279 | 302 (23.6) | 977 (76.4) |  |
| Apr-Jun | 1076 | 241 (22.4) | 835 (77.6) |  |
| Jul-Sep | 1020 | 243 (23.8) | 777 (76.2) |  |
| Oct-Dec | 562 | 110 (19.6) | 452 (80.4) |  |
| **Skilled birth attendance** |  |  |  | <0.001*** |
| No skilled attendant at birth | 1556 | 506 (32.5) | 1050 (67.5) |  |
| Skilled attendant at birth | 2381 | 390 (16.4) | 1991 (83.6) |  |
| **Health card/document** |  |  |  | <0.001*** |
| Does not have health card/document | 1179 | 476 (40.4) | 703 (59.6) |  |
| Yes, none seen | 477 | 119 (24.9) | 358 (75.1) |  |
| Yes, seen | 2281 | 301 (13.2) | 1980 (86.8) |  |
| **Received vitamin A** |  |  |  | <0.001*** |
| No/don’t know | 1283 | 445 (34.7) | 838 (65.3) |  |
| Yes | 2654 | 451 (17.0) | 2203 (83.0) |  |
| **Sex of household head** |  |  |  | 0.190 |
| Male | 3438 | 794 (23.1) | 2644 (76.9) |  |
| Female | 499 | 102 (20.4) | 397 (79.6) |  |
| **Mother's age group** |  |  |  | <0.001*** |
| 15-19 | 183 | 65 (35.5) | 118 (64.5) |  |
| 20-29 | 1965 | 479 (24.4) | 1486 (75.6) |  |
| 30-39 | 1553 | 298 (19.2) | 1255 (80.8) |  |
| 40-49 | 236 | 54 (22.9) | 182 (77.1) |  |
| **Marital status of mother** |  |  |  | 0.330 |
| Never in union | 133 | 35 (26.3) | 98 (73.7) |  |
| Married | 3701 | 833 (22.5) | 2868 (77.5) |  |
| Divorced | 103 | 28 (27.2) | 75 (72.8) |  |
| **Mother employed in the past 12 months** |  |  |  | 0.001 |
| No | 923 | 246 (26.7) | 677 (73.3) |  |
| Yes (currently/in the past 1 year) | 3014 | 650 (21.6) | 2364 (78.4) |  |
| **Mother had problem seeking medical advice or treatment** |  |  |  | <0.001*** |
| Had problem seeking medical advice or treatment | 2010 | 516 (25.7) | 1494 (74.3) |  |
| Did not have problem seeking medical advice or treatment | 1927 | 380 (19.7) | 1547 (80.3) |  |
| **Mother’s education** |  |  |  | <0.001*** |
| No education | 1047 | 388 (37.1) | 659 (62.9) |  |
| Primary | 639 | 161 (25.2) | 478 (74.8) |  |
| Secondary/higher | 2251 | 347 (15.4) | 1904 (84.6) |  |
| **Mother’s religion** |  |  |  | <0.001*** |
| Islam | 1782 | 531 (29.8) | 1251 (70.2) |  |
| Christian | 2126 | 361 (17.0) | 1765 (83.0) |  |
| Traditionalist/others | 29 | 4 (13.8) | 25 (86.2) |  |
| **Mother’s media exposure** |  |  |  | <0.001*** |
| No | 2077 | 564 (27.2) | 1513 (72.8) |  |
| Yes (radio/tv/newspaper at least once a week) | 1860 | 332 (17.8) | 1528 (82.2) |  |
| **Mother’s access to mobile phone/internet** |  |  |  | <0.001*** |
| No | 1500 | 473 (31.5) | 1027 (68.5) |  |
| Yes | 2437 | 423 (17.4) | 2014 (82.6) |  |
| **Mother’s land ownership** |  |  |  | 0.005 |
| Does not own land | 3309 | 780 (23.6) | 2529 (76.4) |  |
| Owns land alone and/or jointly | 628 | 116 (18.5) | 512 (81.5) |  |
| **Mother’s knowledge of malaria** |  |  |  | 0.003 |
| Has no knowledge | 236 | 72 (30.5) | 164 (69.5) |  |
| Has knowledge | 3701 | 824 (22.3) | 2877 (77.7) |  |
| **Mother had health insurance** |  |  |  | 0.024 |
| No | 3823 | 880 (23.0) | 2943 (77.0) |  |
| Yes | 114 | 16 (14.0) | 98 (86.0) |  |
| **Household’s bed net ownership** |  |  |  | 0.005 |
| No | 1264 | 253 (20.0) | 1011 (80.0) |  |
| Yes | 2673 | 643 (24.1) | 2030 (75.9) |  |
| **Mother’s ethnicity** |  |  |  | <0.001*** |
| Hausa/Fulani | 1010 | 345 (34.2) | 665 (65.8) |  |
| Yoruba | 536 | 84 (15.7) | 452 (84.3) |  |
| Igbo | 787 | 96 (12.2) | 691 (87.8) |  |
| Others (ekoi, ibibio, etc.) | 1604 | 371 (23.1) | 1233 (76.9) |  |
| **Household wealth** |  |  |  | <0.001*** |
| Poorer/poorest | 1298 | 429 (33.1) | 869 (66.9) |  |
| Middle | 887 | 205 (23.1) | 682 (76.9) |  |
| Richer/richest | 1752 | 262 (15.0) | 1490 (85.0) |  |
| **Access to bank account** |  |  |  | <0.001*** |
| No | 2972 | 771 (25.9) | 2201 (74.1) |  |
| Yes | 965 | 125 (13.0) | 840 (87.0) |  |
| **Household size** |  |  |  | 0.001 |
| Large (>=9) | 759 | 207 (27.3) | 552 (72.7) |  |
| Medium (5 to 8) | 1854 | 420 (22.7) | 1434 (77.3) |  |
| Small (<=4) | 1324 | 269 (20.3) | 1055 (79.7) |  |
| **Length of stay in household** |  |  |  | <0.001*** |
| <1year/visitor | 147 | 35 (23.8) | 112 (76.2) |  |
| 1-3years | 534 | 80 (15.0) | 454 (85.0) |  |
| 4-5years | 317 | 50 (15.8) | 267 (84.2) |  |
| >5years/always | 2939 | 731 (24.9) | 2208 (75.1) |  |
| **Rural/urban** |  |  |  | <0.001*** |
| Rural | 2242 | 631 (28.1) | 1611 (71.9) |  |
| Urban | 1695 | 265 (15.6) | 1430 (84.4) |  |
| **Livestock density index** |  |  |  | <0.001*** |
| Lower (0-21.4) | 1203 | 227 (18.9) | 976 (81.1) |  |
| Medium (21.5-73.2) | 1332 | 347 (26.1) | 985 (73.9) |  |
| Higher (73.3-5196.8) | 1384 | 320 (23.1) | 1064 (76.9) |  |
| **Travel time to the nearest health facility (providing RI services)** |  |  |  | <0.001*** |
| Lower (0-3.9) | 1395 | 213 (15.3) | 1182 (84.7) |  |
| Medium (4.0-12.5) | 1339 | 310 (23.2) | 1029 (76.8) |  |
| Higher (12.6-532.8) | 1185 | 371 (31.3) | 814 (68.7) |  |
| **Average enhanced vegetation index (2013-2018)** |  |  |  | <0.001*** |
| Lower (0.07-0.23) | 1211 | 345 (28.5) | 866 (71.5) |  |
| Medium (0.24-0.36) | 1514 | 321 (21.2) | 1193 (78.8) |  |
| Higher (0.364-0.57) | 1194 | 228 (19.1) | 966 (80.9) |  |

*p<0.05; **p<0.01; ***p<0.001

**Table G: Percentage of children aged 12 to 23 months with receipt of PENTA3 among those who received PENTA1 vaccinations, according to potential determinants of vaccination, univariate and multivariable analyses of Nigeria DHS 2018**

| **Characteristics and categories** | **Number (%) in category** | **Percentage of receiving PENTA3/1 (95% CI)** | **Frequentist single-**  **level model:**  **cOR [95% CI** | **Bayesian multilevel model:**  **aOR [95% Cr. I]** |
| --- | --- | --- | --- | --- |
| **N** | 3937(100.0) |  |  |  |
| **Sex of child** |  |  |  |  |
| Male | 2038 (33.6) | 77.2 [75.4, 79.0] | 1.00 [reference] | 1.00 [reference] |
| Female | 1899 (31.3) | 77.3 [75.3, 79.1] | 1.00 [0.86, 1.16] | 1.02 [0.86, 1.21] |
| **Birth order** |  |  |  |  |
| 1-2 | 1605 (26.5) | 79.4 [77.3, 81.3] | 1.00 [reference] | 1.00 [reference] |
| >2 | 2332 (38.5) | 75.8 [74.0, 77.5] | 0.81 [0.70, 0.95] ** | 0.83 [0.64, 1.06] |
| **Birth quarter** |  |  |  |  |
| Jan-Mar | 1279 (21.1) | 76.4 [74.0, 78.6] | 0.79 [0.62, 1.01] | 0.77 [0.56, 1.01] |
| Apr-Jun | 1076 (17.8) | 77.6 [75.0, 80.0] | 0.84 [0.65, 1.09] | 0.82 [0.60, 1.09] |
| Jul-Sep | 1020 (16.8) | 76.2 [73.5, 78.7] | 0.78 [0.60, 1.00] | 0.85 [0.62, 1.13] |
| Oct-Dec | 562 (9.3) | 80.4 [76.9, 83.5] | 1.00 [reference] | 1.00 [reference] |
| **Skilled birth attendance** |  |  |  |  |
| No skilled attendant at birth | 1556 (25.7) | 67.5 [65.1, 69.8] | 1.00 [reference] | 1.00 [reference] |
| Skilled attendant at birth | 2381 (39.3) | 83.6 [82.1, 85.1] | 2.46 [2.11, 2.86] *** | 1.25 [0.99, 1.55] |
| **Health card/document** |  |  |  |  |
| Does not have health card/document | 1179 (19.5) | 59.6 [56.8, 62.4] | 1.00 [reference] | 1.00 [reference] |
| Yes, none seen | 477 (7.9) | 75.1 [71.0, 78.7] | 2.04 [1.61, 2.58] *** | 1.74 [1.30, 2.32] ^+^ |
| Yes, seen | 2281 (37.6) | 86.8 [85.4, 88.1] | 4.45 [3.77, 5.27] *** | 5.14 [4.16, 6.32] ^+^ |
| **Received vitamin A** |  |  |  |  |
| No/don’t know | 1283 (21.2) | 65.3 [62.7, 67.9] | 1.00 [reference] | 1.00 [reference] |
| Yes | 2654 (43.8) | 83.0 [81.5, 84.4] | 2.59 [2.23, 3.02] *** | 2.20 [1.79, 2.68] ^+^ |
| **Sex of household head** |  |  |  |  |
| Male | 3438 (56.7) | 76.9 [75.5, 78.3] | 1.00 [reference] | 1.00 [reference] |
| Female | 499 (8.2) | 79.6 [75.8, 82.9] | 1.17 [0.93, 1.47] | 1.03 [0.76, 1.36] |
| **Mother's age group** |  |  |  |  |
| 15-19 | 183 (3.0) | 64.5 [57.3, 71.1] | 1.00 [reference] | 1.00 [reference] |
| 20-29 | 1965 (32.4) | 75.6 [73.7, 77.5] | 1.71 [1.24, 2.35] ** | 1.74 [1.14, 2.52] ^+^ |
| 30-39 | 1553 (25.6) | 80.8 [78.8, 82.7] | 2.32 [1.67, 3.22] *** | 2.42 [1.46, 3.66] ^+^ |
| 40-49 | 236 (3.9) | 77.1 [71.3, 82.0] | 1.86 [1.21, 2.85] ** | 2.39 [1.29, 4.08] ^+^ |
| **Marital status of mother** |  |  |  |  |
| Never in union | 133 (2.2) | 73.7 [65.6, 80.5] | 0.81 [0.55, 1.21] | 0.77 [0.44, 1.28] |
| Married | 3701 (61.1) | 77.5 [76.1, 78.8] | 1.00 [reference] | 1.00 [reference] |
| Divorced | 103 (1.7) | 72.8 [63.4, 80.5] | 0.78 [0.50, 1.21] | 0.74 [0.41, 1.25] |
| **Mother employed in the past 12 months** |  |  |  |  |
| No | 923 (15.2) | 73.3 [70.4, 76.1] | 1.00 [reference] | 1.00 [reference] |
| Yes (currently/in the past 1 year) | 3014 (49.7) | 78.4 [76.9, 79.9] | 1.32 [1.12, 1.57] ** | 1.11 [0.88, 1.36] |
| **Mother had problem seeking medical advice or treatment** |  |  |  |  |
| Did not have problem seeking medical advice or treatment | 2010 (33.2) | 74.3 [72.4, 76.2] | 1.00 [reference] | 1.00 [reference] |
| Had problem seeking medical advice or treatment | 1927 (31.8) | 80.3 [78.4, 82.0] | 1.41 [1.21, 1.63] *** | 0.98 [0.80, 1.18] |
| **Mother’s education** |  |  |  |  |
| No education | 1047 (17.3) | 62.9 [60.0, 65.8] | 1.00 [reference] | 1.00 [reference] |
| Primary | 639 (10.5) | 74.8 [71.3, 78.0] | 1.75 [1.41, 2.17] *** | 1.25 [0.93, 1.65] |
| Secondary/higher | 2251 (37.2) | 84.6 [83.0, 86.0] | 3.23 [2.73, 3.83] *** | 1.70 [1.27, 2.25] ^+^ |
| **Mother’s religion** |  |  |  |  |
| Islam | 1782 (29.4) | 70.2 [68.0, 72.3] | 1.00 [reference] | 1.00 [reference] |
| Christian | 2126 (35.1) | 83.0 [81.4, 84.6] | 2.08 [1.78, 2.42] *** | 1.11 [0.82, 1.48] |
| Traditionalist/others | 29 (0.5) | 86.2 [68.5, 94.7] | 2.65 [0.92, 7.66] | 5.50 [1.20, 17.45] ^+^ |
| **Mother’s media exposure** |  |  |  |  |
| No | 2077 (34.3) | 72.8 [70.9, 74.7] | 1.00 [reference] | 1.00 [reference] |
| Yes (radio/tv/newspaper at least once a week) | 1860 (30.7) | 82.2 [80.3, 83.8] | 1.72 [1.47, 2.00] *** | 0.95 [0.77, 1.17] |
| **Mother’s access to mobile phone/internet** |  |  |  |  |
| No | 1500 (24.8) | 68.5 [66.1, 70.8] | 1.00 [reference] | 1.00 [reference] |
| Yes | 2437 (40.2) | 82.6 [81.1, 84.1] | 2.19 [1.89, 2.55] *** | 1.23 [0.99, 1.53] |
| **Mother’s land ownership** |  |  |  |  |
| Does not own land | 3309 (54.6) | 76.4 [75.0, 77.8] | .. [0.00, 0.00] *** | 1.00 [reference] |
| Owns land alone and/or jointly | 628 (10.4) | 81.5 [78.3, 84.4] | 1.36 [1.10, 1.69] ** | 0.98 [0.74, 1.29] |
| **Mother’s knowledge of malaria** |  |  |  |  |
| Has no knowledge | 236 (3.9) | 69.5 [63.3, 75.0] | 1.00 [reference] | 1.00 [reference] |
| Has knowledge | 3701 (61.1) | 77.7 [76.4, 79.0] | 1.53 [1.15, 2.04] ** | 1.58 [1.11, 2.21] ^+^ |
| **Mother had health insurance** |  |  |  |  |
| No | 3823 (63.1) | 77.0 [75.6, 78.3] | 1.00 [reference] | 1.00 [reference] |
| Yes | 114 (1.9) | 86.0 [78.3, 91.2] | 1.83 [1.07, 3.12] * | 1.23 [0.63, 2.27] |
| **Household’s bed net ownership** |  |  |  |  |
| No | 1264 (20.9) | 80.0 [77.7, 82.1] | 1.00 [reference] | 1.00 [reference] |
| Yes | 2673 (44.1) | 75.9 [74.3, 77.5] | 0.79 [0.67, 0.93] ** | 0.99 [0.80, 1.22] |
| **Mother’s ethnicity** |  |  |  |  |
| Hausa/Fulani | 1010 (16.7) | 65.8 [62.9, 68.7] | 1.00 [reference] | 1.00 [reference] |
| Yoruba | 536 (8.8) | 84.3 [81.0, 87.2] | 2.79 [2.14, 3.64] *** | 1.20 [0.69, 1.88] |
| Igbo | 787 (13.0) | 87.8 [85.3, 89.9] | 3.73 [2.91, 4.79] *** | 1.36 [0.76, 2.26] |
| Others (ekoi, ibibio, etc.) | 1604 (26.5) | 76.9 [74.7, 78.9] | 1.72 [1.45, 2.05] *** | 1.31 [0.94, 1.78] |
| **Household wealth** |  |  |  |  |
| Poorer/poorest | 1298 (21.4) | 66.9 [64.3, 69.5] | 1.00 [reference] | 1.00 [reference] |
| Middle | 887 (14.6) | 76.9 [74.0, 79.5] | 1.64 [1.35, 1.99] *** | 1.05 [0.80, 1.36] |
| Richer/richest | 1752 (28.9) | 85.0 [83.3, 86.6] | 2.81 [2.36, 3.34] *** | 1.24 [0.90, 1.68] |
| **Access to bank account** |  |  |  |  |
| No | 2972 (49.1) | 74.1 [72.5, 75.6] | 1.00 [reference] | 1.00 [reference] |
| Yes | 965 (15.9) | 87.0 [84.8, 89.0] | 2.35 [1.92, 2.89] *** | 1.06 [0.79, 1.38] |
| **Household size** |  |  |  |  |
| Large (>=9) | 759 (12.5) | 72.7 [69.4, 75.8] | 0.78 [0.64, 0.95] * | 1.19 [0.93, 1.50] |
| Medium (5 to 8) | 1854 (30.6) | 77.3 [75.4, 79.2] | 1.00 [reference] | 1.00 [reference] |
| Small (<=4) | 1324 (21.9) | 79.7 [77.4, 81.8] | 1.15 [0.97, 1.36] | 1.01 [0.78, 1.27] |
| **Length of stay in household** |  |  |  |  |
| <1year/visitor | 147 (2.4) | 76.2 [68.6, 82.4] | 0.60 [0.37, 0.97] * | 0.71 [0.38, 1.23] |
| 1-3years | 534 (8.8) | 85.0 [81.7, 87.8] | 1.06 [0.72, 1.56] | 0.98 [0.61, 1.48] |
| 4-5years | 317 (5.2) | 84.2 [79.8, 87.8] | 1.00 [reference] | 1.00 [reference] |
| >5years/always | 2939 (48.5) | 75.1 [73.5, 76.7] | 0.57 [0.41, 0.77] *** | 0.78 [0.53, 1.10] |
| **Rural/urban** |  |  |  |  |
| Rural | 2242 (37.0) | 71.9 [70.0, 73.7] | 1.00 [reference] | 1.00 [reference] |
| Urban | 1695 (28.0) | 84.4 [82.6, 86.0] | 2.11 [1.80, 2.48] *** | 1.18 [0.74, 1.77] |
| **Livestock density index** |  |  |  |  |
| Lower (0-21.4) | 1203 (19.9) | 81.1 [78.8, 83.2] | 1.29 [1.07, 1.57] ** | 1.29 [0.90, 1.80] |
| Medium (21.5-73.2) | 1332 (22.0) | 73.9 [71.5, 76.2] | 0.85 [0.72, 1.02] | 0.98 [0.73, 1.27] |
| Higher (73.3-5196.8) | 1384 (22.8) | 76.9 [74.6, 79.0] | 1.00 [reference] | 1.00 [reference] |
| **Travel time to the nearest health facility (providing RI services)** |  |  |  |  |
| Lower (0-3.9) | 1395 (23.0) | 84.7 [82.7, 86.5] | 2.67 [2.20, 3.23] *** | 1.21 [0.79, 1.78] |
| Medium (4.0-12.5) | 1339 (22.1) | 76.8 [74.5, 79.0] | 1.54 [1.29, 1.84] *** | 1.16 [0.90, 1.48] |
| Higher (12.6-532.8) | 1185 (19.6) | 68.7 [66.0, 71.3] | 1.00 [reference] | 1.00 [reference] |
| **Average enhanced vegetation index (2013-2018)** |  |  |  |  |
| Lower (0.07-0.23) | 1211 (20.0) | 71.5 [68.9, 74.0] | 1.00 [reference] | 1.00 [reference] |
| Medium (0.24-0.36) | 1514 (25.0) | 78.8 [76.7, 80.8] | 1.48 [1.24, 1.76] *** | 1.33 [0.99, 1.77] |
| Higher (0.364-0.57) | 1194 (19.7) | 80.9 [78.6, 83.0] | 1.69 [1.39, 2.04] *** | 0.94 [0.65, 1.33] |
|  |  |  |  |  |
| **Variance parameters** |  |  |  | **Estimate [95% Cr. I]** |
| Stratum-level variance |  |  |  | 0.44 [0.24, 0.74] |
| Cluster-level variance |  |  |  | 0.14 [0.00, 0.39] |

CI: Confidence Interval; Cr. I: Bayesian Credible Interval; cOR: Crude Odds Ratio; aOR: adjusted Odds Ratio; ^+^Significant covariate for the Bayesian model; *p<0.05; **p<0.01; ***p<0.001.

**Interpretation of factors associated with PENTA3/1 vaccination in the binomial analysis**

In multivariable analysis, the effect of having a HBR was smaller than for PENTA1 and presence of a SBA at birth just failed to reach statistical significance (aOR=1.25, 95% Cr.I: 0.99, 1.55). Factors significantly associated with higher odds of PENTA3/1 receipt are health card/document (yes, none seen: aOR=1.74, 95% Cr.I: 1.30, 2.32; yes, seen: aOR=5.14, 95% Cr.I: 4.16, 6.32), receipt of vitamin A (aOR=2.20, 95% Cr.I: 1.79, 2.68), maternal age (age 20-29: aOR=1.74, 95% Cr.I: 1.14, 2.52; age 30-39: aOR=2.42, 95% Cr.I: 1.46, 3.66; age 40-49: aOR=2.39, 95% Cr.I: 1.29, 4.08), secondary/higher maternal education (aOR=1.70, 95% Cr.I: 1.27, 2.25) and mothers who had knowledge about malaria (aOR=1.58, 95% Cr.I: 1.11, 2.21).

Christian religion was no longer significant but PENTA3/1 receipt was much higher in children of the small number of mothers with traditional or other religions (aOR=5.50, 95% Cr.I: 1.20, 17.45) compared with those from mothers who practice Islamic religion. Access to a bank account or mobile phone/internet, length of stay in residence and geospatial variables were not significantly associated with completion of the PENTA series. No significant interaction terms were observed between PENTA3/1 and significant predictors (Table G).

**Additional results of the binomial analysis for MV**

**Table H: Factors associated with any evidence of MV vaccination, cross-tabulation analyses of Nigeria DHS 2018**

|  |  | **MV** | |  |
| --- | --- | --- | --- | --- |
|  |  | **No** | **Yes** |  |
| **Variables** | **N** | **n (%)** | **n (%)** | **P-value** |
| N | 11839 | 5206 (44) | 6633 (56) |  |
| **Sex of child** |  |  |  | 0.420 |
| Male | 6050 | 2682 (44.3) | 3368 (55.7) |  |
| Female | 5789 | 2524 (43.6) | 3265 (56.4) |  |
| **Birth order** |  |  |  | <0.001*** |
| 1-2 | 4485 | 1769 (39.4) | 2716 (60.6) |  |
| >2 | 7354 | 3437 (46.7) | 3917 (53.3) |  |
| **Birth quarter** |  |  |  | <0.001*** |
| Jan-Mar | 3801 | 1611 (42.4) | 2190 (57.6) |  |
| Apr-Jun | 3237 | 1471 (45.4) | 1766 (54.6) |  |
| Jul-Sep | 3194 | 1478 (46.3) | 1716 (53.7) |  |
| Oct-Dec | 1607 | 646 (40.2) | 961 (59.8) |  |
| **Skilled birth attendance** |  |  |  | <0.001*** |
| No skilled attendant at birth | 6509 | 3887 (59.7) | 2622 (40.3) |  |
| Skilled attendant at birth | 5330 | 1319 (24.7) | 4011 (75.3) |  |
| **Health card/document** |  |  |  | <0.001*** |
| Does not have health card/document | 6600 | 3884 (58.8) | 2716 (41.2) |  |
| Yes, none seen | 1104 | 231 (20.9) | 873 (79.1) |  |
| Yes, seen | 4135 | 1091 (26.4) | 3044 (73.6) |  |
| **Received vitamin A** |  |  |  | <0.001*** |
| No/don’t know | 5658 | 3595 (63.5) | 2063 (36.5) |  |
| Yes | 6181 | 1611 (26.1) | 4570 (73.9) |  |
| **Sex of household head** |  |  |  | <0.001*** |
| Male | 10662 | 4826 (45.3) | 5836 (54.7) |  |
| Female | 1177 | 380 (32.3) | 797 (67.7) |  |
| **Mother's age group** |  |  |  | <0.001*** |
| 15-19 | 587 | 370 (63.0) | 217 (37.0) |  |
| 20-29 | 5766 | 2617 (45.4) | 3149 (54.6) |  |
| 30-39 | 4508 | 1748 (38.8) | 2760 (61.2) |  |
| 40-49 | 978 | 471 (48.2) | 507 (51.8) |  |
| **Marital status of mother** |  |  |  | <0.001*** |
| Never in union | 287 | 96 (33.4) | 191 (66.6) |  |
| Married | 11222 | 4982 (44.4) | 6240 (55.6) |  |
| Divorced | 330 | 128 (38.8) | 202 (61.2) |  |
| **Mother employed in the past 12 months** |  |  |  | <0.001*** |
| No | 3403 | 1926 (56.6) | 1477 (43.4) |  |
| Yes (currently/in the past 1 year) | 8436 | 3280 (38.9) | 5156 (61.1) |  |
| **Mother had problem seeking medical advice or treatment** |  |  |  | <0.001*** |
| Had problem seeking medical advice or treatment | 6529 | 3256 (49.9) | 3273 (50.1) |  |
| Did not have problem seeking medical advice or treatment | 5310 | 1950 (36.7) | 3360 (63.3) |  |
| **Mother’s education** |  |  |  | <0.001*** |
| No education | 5112 | 3262 (63.8) | 1850 (36.2) |  |
| Primary | 1784 | 770 (43.2) | 1014 (56.8) |  |
| Secondary/higher | 4943 | 1174 (23.8) | 3769 (76.2) |  |
| **Mother’s religion** |  |  |  | <0.001*** |
| Islam | 6901 | 3849 (55.8) | 3052 (44.2) |  |
| Christian | 4835 | 1321 (27.3) | 3514 (72.7) |  |
| Traditionalist/others | 103 | 36 (35.0) | 67 (65.0) |  |
| **Mother’s media exposure** |  |  |  | <0.001*** |
| No | 7247 | 3868 (53.4) | 3379 (46.6) |  |
| Yes (radio/tv/newspaper at least once a week) | 4592 | 1338 (29.1) | 3254 (70.9) |  |
| **Mother’s access to mobile phone/internet** |  |  |  | <0.001*** |
| No | 5867 | 3424 (58.4) | 2443 (41.6) |  |
| Yes | 5972 | 1782 (29.8) | 4190 (70.2) |  |
| **Mother’s land ownership** |  |  |  | <0.001*** |
| Does not own land | 10247 | 4671 (45.6) | 5576 (54.4) |  |
| Owns land alone and/or jointly | 1592 | 535 (33.6) | 1057 (66.4) |  |
| **Mother’s knowledge of malaria** |  |  |  | <0.001*** |
| Has no knowledge | 685 | 347 (50.7) | 338 (49.3) |  |
| Has knowledge | 11154 | 4859 (43.6) | 6295 (56.4) |  |
| **Mother had health insurance** |  |  |  | <0.001*** |
| No | 11578 | 5151 (44.5) | 6427 (55.5) |  |
| Yes | 261 | 55 (21.1) | 206 (78.9) |  |
| **Household’s bed net ownership** |  |  |  | <0.001*** |
| No | 3543 | 1432 (40.4) | 2111 (59.6) |  |
| Yes | 8296 | 3774 (45.5) | 4522 (54.5) |  |
| **Mother’s ethnicity** |  |  |  | <0.001*** |
| Hausa/Fulani | 4728 | 2899 (61.3) | 1829 (38.7) |  |
| Yoruba | 1201 | 294 (24.5) | 907 (75.5) |  |
| Igbo | 1667 | 367 (22.0) | 1300 (78.0) |  |
| Others (ekoi, ibibio, etc.) | 4243 | 1646 (38.8) | 2597 (61.2) |  |
| **Household wealth** |  |  |  | <0.001*** |
| Poorer/poorest | 5353 | 3205 (59.9) | 2148 (40.1) |  |
| Middle | 2478 | 1039 (41.9) | 1439 (58.1) |  |
| Richer/richest | 4008 | 962 (24.0) | 3046 (76.0) |  |
| **Access to bank account** |  |  |  | <0.001*** |
| No | 9844 | 4923 (50.0) | 4921 (50.0) |  |
| Yes | 1995 | 283 (14.2) | 1712 (85.8) |  |
| **Household size** |  |  |  | <0.001*** |
| Large (>=9) | 2946 | 1599 (54.3) | 1347 (45.7) |  |
| Medium (5 to 8) | 5518 | 2287 (41.4) | 3231 (58.6) |  |
| Small (<=4) | 3375 | 1320 (39.1) | 2055 (60.9) |  |
| **Length of stay in household** |  |  |  | <0.001*** |
| <1year/visitor | 345 | 106 (30.7) | 239 (69.3) |  |
| 1-3years | 1205 | 413 (34.3) | 792 (65.7) |  |
| 4-5years | 864 | 315 (36.5) | 549 (63.5) |  |
| >5years/always | 9425 | 4372 (46.4) | 5053 (53.6) |  |
| **Rural/urban** |  |  |  | <0.001*** |
| Rural | 7622 | 3908 (51.3) | 3714 (48.7) |  |
| Urban | 4217 | 1298 (30.8) | 2919 (69.2) |  |
| **Livestock density index** |  |  |  | <0.001*** |
| Lower (0-21.4) | 3189 | 1096 (34.4) | 2093 (65.6) |  |
| Medium (21.5-73.2) | 4029 | 1830 (45.4) | 2199 (54.6) |  |
| Higher (73.3-5196.8) | 4621 | 2280 (49.3) | 2341 (50.7) |  |
| **Travel time to the nearest health facility (providing RI services)** |  |  |  | <0.001*** |
| Lower (0-3.9) | 3406 | 995 (29.2) | 2411 (70.8) |  |
| Medium (4.0-12.5) | 3968 | 1713 (43.2) | 2255 (56.8) |  |
| Higher (12.6-532.8) | 4465 | 2498 (55.9) | 1967 (44.1) |  |
| **Average enhanced vegetation index (2013-2018)** |  |  |  | <0.001*** |
| Lower (0.07-0.23) | 4759 | 2502 (52.6) | 2257 (47.4) |  |
| Medium (0.24-0.36) | 4270 | 1815 (42.5) | 2455 (57.5) |  |
| Higher (0.364-0.57) | 2810 | 889 (31.6) | 1921 (68.4) |  |

*p<0.05; **p<0.01; ***p<0.001

**Table I: Percentage of children aged 12 to 35 months with any evidence of MV vaccination, according to potential determinants of vaccination, univariate and multivariable analyses of Nigeria DHS 2018**

| **Characteristics and categories** | **Number (%) in category** | **Percentage with any evidence of MV (95% CI)** | **Frequentist single-**  **level model:**  **cOR [95% CI** | **Bayesian multilevel model:**  **aOR [95% Cr. I]** |
| --- | --- | --- | --- | --- |
| **N** | **11839** |  |  |  |
| **Sex of child** |  |  |  |  |
| Male | 6050 (51.1) | 55.7 [54.4, 56.9] | 1.00 [reference] | 1.00 [reference] |
| Female | 5789 (48.9) | 56.4 [55.1, 57.7] | 1.03 [0.94, 1.11] | 1.03 [0.94, 1.13] |
| **Birth order** |  |  |  |  |
| 1-2 | 4485 (37.9) | 60.6 [59.1, 62.0] | 1.00 [reference] | 1.00 [reference] |
| >2 | 7354 (62.1) | 53.3 [52.1, 54.4] | 0.74 [0.69, 0.80] *** | 0.83 [0.72, 0.95] ^+^ |
| **Birth quarter** |  |  |  |  |
| Jan-Mar | 3801 (32.1) | 57.6 [56.0, 59.2] | 0.91 [0.81, 1.03] | 0.99 [0.85, 1.15] |
| Apr-Jun | 3237 (27.3) | 54.6 [52.8, 56.3] | 0.81 [0.71, 0.91] ** | 0.90 [0.77, 1.04] |
| Jul-Sep | 3194 (27.0) | 53.7 [52.0, 55.4] | 0.78 [0.69, 0.88] *** | 0.89 [0.75, 1.03] |
| Oct-Dec | 1607 (13.6) | 59.8 [57.4, 62.2] | 1.00 [reference] | 1.00 [reference] |
| **Skilled birth attendance** |  |  |  |  |
| No skilled attendant at birth | 6509 (55.0) | 40.3 [39.1, 41.5] | 1.00 [reference] | 1.00 [reference] |
| Skilled attendant at birth | 5330 (45.0) | 75.3 [74.1, 76.4] | 4.51 [4.16, 4.88] *** | 1.66 [1.46, 1.88] ^+^ |
| **Health card/document** |  |  |  |  |
| Does not have health card/document | 6600 (55.7) | 41.2 [40.0, 42.3] | 1.00 [reference] | 1.00 [reference] |
| Yes, none seen | 1104 (9.3) | 79.1 [76.6, 81.4] | 5.40 [4.64, 6.30] *** | 3.43 [2.81, 4.13] ^+^ |
| Yes, seen | 4135 (34.9) | 73.6 [72.3, 74.9] | 3.99 [3.67, 4.34] *** | 2.17 [1.94, 2.42] ^+^ |
| **Received vitamin A** |  |  |  |  |
| No/don’t know | 5658 (47.8) | 36.5 [35.2, 37.7] | 1.00 [reference] | 1.00 [reference] |
| Yes | 6181 (52.2) | 73.9 [72.8, 75.0] | 4.94 [4.57, 5.35] *** | 3.42 [3.06, 3.82] ^+^ |
| **Sex of household head** |  |  |  |  |
| Male | 10662 (90.1) | 54.7 [53.8, 55.7] | 1.00 [reference] | 1.00 [reference] |
| Female | 1177 (9.9) | 67.7 [65.0, 70.3] | 1.73 [1.53, 1.97] *** | 1.04 [0.87, 1.24] |
| **Mother's age group** |  |  |  |  |
| 15-19 | 587 (5.0) | 37.0 [33.2, 41.0] | 1.00 [reference] | 1.00 [reference] |
| 20-29 | 5766 (48.7) | 54.6 [53.3, 55.9] | 2.05 [1.72, 2.45] *** | 1.57 [1.24, 1.94] ^+^ |
| 30-39 | 4508 (38.1) | 61.2 [59.8, 62.6] | 2.69 [2.25, 3.22] *** | 1.88 [1.45, 2.38] ^+^ |
| 40-49 | 978 (8.3) | 51.8 [48.7, 55.0] | 1.84 [1.49, 2.26] *** | 1.55 [1.12, 2.03] ^+^ |
| **Marital status of mother** |  |  |  |  |
| Never in union | 287 (2.4) | 66.6 [60.9, 71.8] | 1.59 [1.24, 2.04] *** | 1.07 [0.76, 1.46] |
| Married | 11222 (94.8) | 55.6 [54.7, 56.5] | 1.00 [reference] | 1.00 [reference] |
| Divorced | 330 (2.8) | 61.2 [55.8, 66.3] | 1.26 [1.01, 1.58] * | 1.15 [0.85, 1.55] |
| **Mother employed in the past 12 months** |  |  |  |  |
| No | 3403 (28.7) | 43.4 [41.7, 45.1] | 1.00 [reference] | 1.00 [reference] |
| Yes (currently/in the past 1 year) | 8436 (71.3) | 61.1 [60.1, 62.2] | 2.05 [1.89, 2.22] *** | 1.35 [1.20, 1.50] ^+^ |
| **Mother had problem seeking medical advice or treatment** |  |  |  |  |
| Did not have problem seeking medical advice or treatment | 6529 (55.1) | 50.1 [48.9, 51.3] | 1.00 [reference] | 1.00 [reference] |
| Had problem seeking medical advice or treatment | 5310 (44.9) | 63.3 [62.0, 64.6] | 1.71 [1.59, 1.85] *** | 1.19 [1.06, 1.32] ^+^ |
| **Mother’s education** |  |  |  |  |
| No education | 5112 (43.2) | 36.2 [34.9, 37.5] | 1.00 [reference] | 1.00 [reference] |
| Primary | 1784 (15.1) | 56.8 [54.5, 59.1] | 2.32 [2.08, 2.59] *** | 1.18 [1.00, 1.36] ^+^ |
| Secondary/higher | 4943 (41.8) | 76.2 [75.0, 77.4] | 5.66 [5.19, 6.17] *** | 1.50 [1.28, 1.75] ^+^ |
| **Mother’s religion** |  |  |  |  |
| Islam | 6901 (58.3) | 44.2 [43.1, 45.4] | 1.00 [reference] | 1.00 [reference] |
| Christian | 4835 (40.8) | 72.7 [71.4, 73.9] | 3.35 [3.10, 3.63] *** | 1.15 [0.95, 1.38] |
| Traditionalist/others | 103 (0.9) | 65.0 [55.4, 73.6] | 2.35 [1.56, 3.53] *** | 1.60 [0.88, 2.74] |
| **Mother’s media exposure** |  |  |  |  |
| No | 7247 (61.2) | 46.6 [45.5, 47.8] | 1.00 [reference] | 1.00 [reference] |
| Yes (radio/tv/newspaper at least once a week) | 4592 (38.8) | 70.9 [69.5, 72.2] | 2.78 [2.57, 3.01] *** | 1.21 [1.08, 1.36] ^+^ |
| **Mother’s access to mobile phone/internet** |  |  |  |  |
| No | 5867 (49.6) | 41.6 [40.4, 42.9] | 1.00 [reference] | 1.00 [reference] |
| Yes | 5972 (50.4) | 70.2 [69.0, 71.3] | 3.30 [3.05, 3.56] *** | 1.22 [1.08, 1.36] ^+^ |
| **Mother’s land ownership** |  |  |  |  |
| Does not own land | 10247 (86.6) | 54.4 [53.4, 55.4] | 1.00 [reference] | 1.00 [reference] |
| Owns land alone and/or jointly | 1592 (13.4) | 66.4 [64.0, 68.7] | 1.66 [1.48, 1.85] *** | 1.13 [0.96, 1.32] |
| **Mother’s knowledge of malaria** |  |  |  |  |
| Has no knowledge | 685 (5.8) | 49.3 [45.6, 53.1] | 1.00 [reference] | 1.00 [reference] |
| Has knowledge | 11154 (94.2) | 56.4 [55.5, 57.4] | 1.33 [1.14, 1.55] *** | 1.10 [0.89, 1.35] |
| **Mother had health insurance** |  |  |  |  |
| No | 11578 (97.8) | 55.5 [54.6, 56.4] | 1.00 [reference] | 1.00 [reference] |
| Yes | 261 (2.2) | 78.9 [73.6, 83.5] | 3.00 [2.22, 4.05] *** | 1.59 [1.03, 2.40] ^+^ |
| **Household’s bed net ownership** |  |  |  |  |
| No | 3543 (29.9) | 59.6 [58.0, 61.2] | 1.00 [reference] | 1.00 [reference] |
| Yes | 8296 (70.1) | 54.5 [53.4, 55.6] | 0.81 [0.75, 0.88] *** | 1.16 [1.04, 1.30] ^+^ |
| **Mother’s ethnicity** |  |  |  |  |
| Hausa/Fulani | 4728 (39.9) | 38.7 [37.3, 40.1] | 1.00 [reference] | 1.00 [reference] |
| Yoruba | 1201 (10.1) | 75.5 [73.0, 77.9] | 4.89 [4.23, 5.65] *** | 1.22 [0.88, 1.66] |
| Igbo | 1667 (14.1) | 78.0 [75.9, 79.9] | 5.61 [4.93, 6.39] *** | 1.56 [1.08, 2.20] ^+^ |
| Others (ekoi, ibibio, etc.) | 4243 (35.8) | 61.2 [59.7, 62.7] | 2.50 [2.30, 2.72] *** | 1.24 [1.03, 1.48] ^+^ |
| **Household wealth** |  |  |  |  |
| Poorer/poorest | 5353 (45.2) | 40.1 [38.8, 41.4] | 1.00 [reference] | 1.00 [reference] |
| Middle | 2478 (20.9) | 58.1 [56.1, 60.0] | 2.07 [1.88, 2.28] *** | 1.09 [0.94, 1.25] |
| Richer/richest | 4008 (33.9) | 76.0 [74.7, 77.3] | 4.72 [4.31, 5.17] *** | 1.31 [1.09, 1.55] ^+^ |
| **Access to bank account** |  |  |  |  |
| No | 9844 (83.1) | 50.0 [49.0, 51.0] | 1.00 [reference] | 1.00 [reference] |
| Yes | 1995 (16.9) | 85.8 [84.2, 87.3] | 6.05 [5.30, 6.90] *** | 1.79 [1.50, 2.12] ^+^ |
| **Household size** |  |  |  |  |
| Large (>=9) | 2946 (24.9) | 45.7 [43.9, 47.5] | 0.60 [0.54, 0.65] *** | 0.96 [0.85, 1.08] |
| Medium (5 to 8) | 5518 (46.6) | 58.6 [57.2, 59.8] | 1.00 [reference] | 1.00 [reference] |
| Small (<=4) | 3375 (28.5) | 60.9 [59.2, 62.5] | 1.10 [1.01, 1.20] * | 0.96 [0.84, 1.09] |
| **Length of stay in household** |  |  |  |  |
| <1year/visitor | 345 (2.9) | 69.3 [64.2, 73.9] | 1.29 [0.99, 1.69] | 1.20 [0.83, 1.65] |
| 1-3years | 1205 (10.2) | 65.7 [63.0, 68.4] | 1.10 [0.92, 1.32] | 1.10 [0.87, 1.40] |
| 4-5years | 864 (7.3) | 63.5 [60.3, 66.7] | 1.00 [reference] | 1.00 [reference] |
| >5years/always | 9425 (79.6) | 53.6 [52.6, 54.6] | 0.66 [0.57, 0.77] *** | 1.15 [0.95, 1.40] |
| **Rural/urban** |  |  |  |  |
| Rural | 7622 (64.4) | 48.7 [47.6, 49.9] | 1.00 [reference] | 1.00 [reference] |
| Urban | 4217 (35.6) | 69.2 [67.8, 70.6] | 2.37 [2.19, 2.56] *** | 0.98 [0.68, 1.37] |
| **Livestock density index** |  |  |  |  |
| Lower (0-21.4) | 3189 (26.9) | 65.6 [64.0, 67.3] | 1.86 [1.69, 2.04] *** | 1.62 [1.28, 2.06] ^+^ |
| Medium (21.5-73.2) | 4029 (34.0) | 54.6 [53.0, 56.1] | 1.17 [1.08, 1.27] *** | 1.15 [0.94, 1.39] |
| Higher (73.3-5196.8) | 4621 (39.0) | 50.7 [49.2, 52.1] | 1.00 [reference] | 1.00 [reference] |
| **Travel time to the nearest health facility (providing RI services)** |  |  |  |  |
| Lower (0-3.9) | 3406 (28.8) | 70.8 [69.2, 72.3] | 3.16 [2.88, 3.48] *** | 1.14 [0.87, 1.46] |
| Medium (4.0-12.5) | 3968 (33.5) | 56.8 [55.3, 58.4] | 1.69 [1.55, 1.84] *** | 1.23 [1.03, 1.44] ^+^ |
| Higher (12.6-532.8) | 4465 (37.7) | 44.1 [42.6, 45.5] | 1.00 [reference] | 1.00 [reference] |
| **Variance parameters** |  |  |  | **Estimate (95% Cr. I)** |
| Stratum-level variance |  |  |  | 0.39 [0.25, 0.59] |
| Cluster-level variance |  |  |  | 0.40 [0.30, 0.50] |

CI: Confidence Interval; Cr. I: Bayesian Credible Interval; cOR: Crude Odds Ratio; aOR: adjusted Odds Ratio; ^+^Significant covariate for the Bayesian model; *p<0.05; **p<0.01; ***p<0.001.

**Interpretation of factors associated with MV vaccination in the binomial analysis**

Presence of SBA at the child’s delivery (aOR=1.66, 95% Cr.I: 1.46, 1.88), health card (yes, none seen: aOR=3.43, 95% Cr.I: 2.81, 4.13; yes, seen: aOR=2.17, 95% Cr.I: 1.94, 2.42), receipt of Vitamin A (aOR=3.42, 95% Cr.I: 3.06, 3.82) were individual variables associated with increased odds for receipt of MV. Maternal or household factors significantly associated with higher odds of MV receipt include maternal age (age 20-29: aOR=1.57, 95% Cr.I: 1.24, 1.94; age 30-39: aOR=1.88, 95% Cr.I: 1.45, 2.38; age 40-49: aOR=1.55, 95% Cr.I: 1.12, 2.03), mothers who are currently employed (aOR=1.35, 95% Cr.I: 1.20, 1.50), problem seeking medical advice (aOR=1.19, 95% Cr.I: 1.06, 1.32), maternal education (primary: aOR=1.18, 95% Cr.I: 1.00, 1.36; secondary: aOR=1.50, 95% Cr.I: 1.28, 1.75), mother’s exposed to the media (aOR=1.21, 95% Cr.I: 1.08, 1.36), access to mobile phone/internet (aOR=1.22, 95% Cr.I: 1.08, 1.36), health insurance (aOR=1.59, 95% Cr.I: 1.03, 2.40), households bed net ownership (aOR=1.16, 95% Cr.I: 1.04, 1.30), ethnicity (Igbo: aOR=1.56, 95% Cr.I: 1.08, 2.20; Others (Ekoi, Ibibio, etc): aOR=1.24, 95% Cr.I: 1.03, 1.48), richer/richest wealth category (aOR=1.31, 95% Cr.I: 1.09, 1.55) and access to bank account (aOR=1.79, 95% Cr.I: 1.50, 2.12). Higher birth order (aOR=0.83, 95% Cr.I: 0.72, 0.95) was associated with decreased odds of receipt of MV. Of the community/geospatial variables, only residence in communities with lower livestock density index (aOR=1.62, 95% Cr.I: 1.28, 2.06) and medium travel time to nearest health facility (aOR=1.23, 95% Cr.I: 1.03, 1.44) were significantly associated with increased odds of MV receipt. (Table I).

**Predictive accuracy of the Bayesian multiple multilevel binomial models for PENTA1, PENTA3/1 and MV**

Even though this is not the main goal of the study, we attempt to evaluate the discriminatory or predictive power of our Bayesian multilevel binomial multivariable models for correctly predicting PENTA1, PENTA3/1, and MV vaccination coverages in this group of children via the Area Under the Receiver Operating Characteristic (AUROC) curve. The AUROC curve value of 91.3% for PENTA1 in Fig C is suggestive of an outstanding discriminatory/predictive ability[20] for our model to correctly predict PENTA1 vaccination coverage. Also, the AUROC curve value of 77.1% and 80.2% in Fig S3 for PENTA3/1 and MV respectively indicates good predictive ability [20] of the fitted models to correctly predict PENTA3/1, and MV. This is an indication that routine vaccination (PENTA1) is relatively more predictable compared to vaccinations through campaigns (MV).


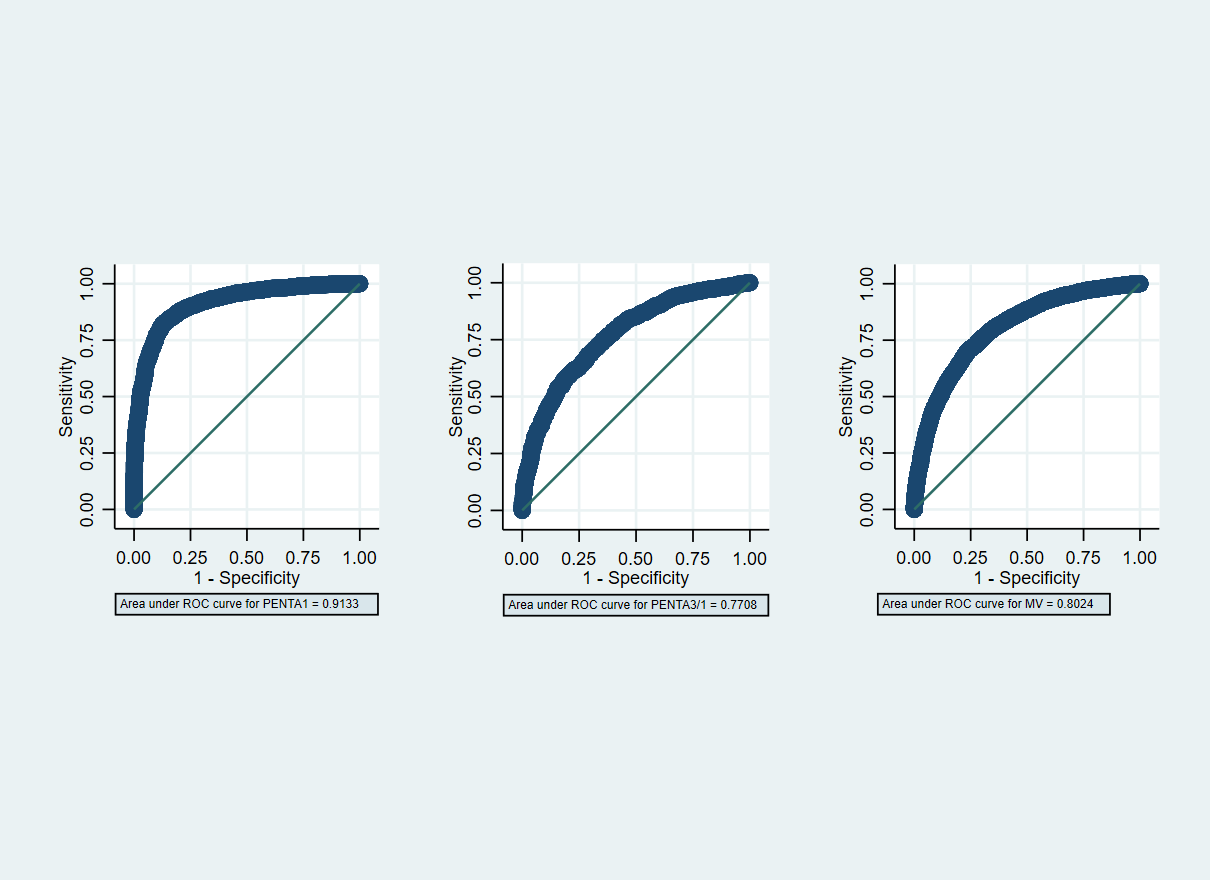


**Fig C. Area Under Receiver Operating Characteristic curve of the Bayesian multilevel binomial multivariable models for predicting PENTA1 (1^st^ column), PENTA3/1 (2^nd^ column) and MV (3^rd^ column) vaccination coverages in Nigeria.**

**Additional results for the multinomial analyses**

Presented in this section are additional results from the Bayesian multiple multilevel multinomial analyses for PENTA1 and MV.

**PENTA1**

**Table J: Factors associated with PENTA1 vaccination in the multinomial cross-tabulation analyses of Nigeria DHS 2018**

|  |  | **PENTA1** | | |  |
| --- | --- | --- | --- | --- | --- |
|  |  | **No evidence of vaccination** | **Card invalid/ history** | **Card valid** |  |
| Factor | **N** | **n (%)** | **n (%)** | **n (%)** | **P-value** |
| N | 6059 | 2122 (35.0) | 1898 (31.3) | 2039 (33.7) |  |
| **Sex of child** |  |  |  |  | 0.820 |
| Male | 3148 | 1110 (35.3) | 990 (31.4) | 1048 (33.3) |  |
| Female | 2911 | 1012 (34.8) | 908 (31.2) | 991 (34.0) |  |
| **Birth order** |  |  |  |  | <0.001*** |
| 1-2 | 2279 | 674 (29.6) | 742 (32.6) | 863 (37.9) |  |
| >2 | 3780 | 1448 (38.3) | 1156 (30.6) | 1176 (31.1) |  |
| **Birth quarter** |  |  |  |  | 0.170 |
| Jan-Mar | 1909 | 630 (33.0) | 616 (32.3) | 663 (34.7) |  |
| Apr-Jun | 1668 | 592 (35.5) | 516 (30.9) | 560 (33.6) |  |
| Jul-Sep | 1628 | 608 (37.3) | 483 (29.7) | 537 (33.0) |  |
| Oct-Dec | 854 | 292 (34.2) | 283 (33.1) | 279 (32.7) |  |
| **Skilled birth attendance** |  |  |  |  | <0.001*** |
| No skilled attendant at birth | 3328 | 1772 (53.2) | 778 (23.4) | 778 (23.4) |  |
| Skilled attendant at birth | 2731 | 350 (12.8) | 1120 (41.0) | 1261 (46.2) |  |
| **Health card/document** |  |  |  |  | <0.001*** |
| Does not have health card/document | 3113 | 1934 (62.1) | 1179 (37.9) | 0 (0.0) |  |
| Yes, none seen | 519 | 42 (8.1) | 477 (91.9) | 0 (0.0) |  |
| Yes, seen | 2427 | 146 (6.0) | 242 (10.0) | 2039 (84.0) |  |
| **Received vitamin A** |  |  |  |  | <0.001*** |
| No/don’t know | 2852 | 1569 (55.0) | 662 (23.2) | 621 (21.8) |  |
| Yes | 3207 | 553 (17.2) | 1236 (38.5) | 1418 (44.2) |  |
| **Sex of household head** |  |  |  |  | <0.001*** |
| Female | 5434 | 1996 (36.7) | 1632 (30.0) | 1806 (33.2) |  |
| Male | 625 | 126 (20.2) | 266 (42.6) | 233 (37.3) |  |
| **Mother's age group** |  |  |  |  | <0.001*** |
| 15-19 | 366 | 183 (50.0) | 80 (21.9) | 103 (28.1) |  |
| 20-29 | 3019 | 1054 (34.9) | 962 (31.9) | 1003 (33.2) |  |
| 30-39 | 2237 | 684 (30.6) | 743 (33.2) | 810 (36.2) |  |
| 40-49 | 437 | 201 (46.0) | 113 (25.9) | 123 (28.1) |  |
| **Marital status of mother** |  |  |  |  | 0.004 |
| Never in union | 171 | 38 (22.2) | 66 (38.6) | 67 (39.2) |  |
| Married | 5730 | 2029 (35.4) | 1774 (31.0) | 1927 (33.6) |  |
| Divorced | 158 | 55 (34.8) | 58 (36.7) | 45 (28.5) |  |
| **Mother employed in the past 12 months** |  |  |  |  | <0.001*** |
| No | 1777 | 854 (48.1) | 418 (23.5) | 505 (28.4) |  |
| Yes (currently/in the past 1 year) | 4282 | 1268 (29.6) | 1480 (34.6) | 1534 (35.8) |  |
| **Mother had problem seeking medical advice or treatment** |  |  |  |  | <0.001*** |
| Had problem seeking medical advice or treatment | 3397 | 1387 (40.8) | 984 (29.0) | 1026 (30.2) |  |
| Did not have problem seeking medical advice or treatment | 2662 | 735 (27.6) | 914 (34.3) | 1013 (38.1) |  |
| **Mother’s education** |  |  |  |  | <0.001*** |
| No education | 2614 | 1567 (59.9) | 552 (21.1) | 495 (18.9) |  |
| Primary | 881 | 242 (27.5) | 309 (35.1) | 330 (37.5) |  |
| Secondary/higher | 2564 | 313 (12.2) | 1037 (40.4) | 1214 (47.3) |  |
| **Religion** |  |  |  |  | <0.001*** |
| Islam | 3538 | 1756 (49.6) | 894 (25.3) | 888 (25.1) |  |
| Christian | 2469 | 343 (13.9) | 982 (39.8) | 1144 (46.3) |  |
| Traditionalist/others | 52 | 23 (44.2) | 22 (42.3) | 7 (13.5) |  |
| **Mother’s media exposure** |  |  |  |  | <0.001*** |
| No | 3729 | 1652 (44.3) | 1012 (27.1) | 1065 (28.6) |  |
| Yes (radio/tv/newspaper at least once a week) | 2330 | 470 (20.2) | 886 (38.0) | 974 (41.8) |  |
| **Mother’s access to mobile phone/internet** |  |  |  |  | <0.001*** |
| No | 3062 | 1562 (51.0) | 740 (24.2) | 760 (24.8) |  |
| Yes | 2997 | 560 (18.7) | 1158 (38.6) | 1279 (42.7) |  |
| **Mother’s land ownership** |  |  |  |  | <0.001*** |
| Does not own land | 5236 | 1927 (36.8) | 1594 (30.4) | 1715 (32.8) |  |
| Owns land alone and/or jointly | 823 | 195 (23.7) | 304 (36.9) | 324 (39.4) |  |
| **Mother’s knowledge of malaria** |  |  |  |  | 0.009 |
| Has no knowledge | 386 | 150 (38.9) | 94 (24.4) | 142 (36.8) |  |
| Has knowledge | 5673 | 1972 (34.8) | 1804 (31.8) | 1897 (33.4) |  |
| **Mother had health insurance** |  |  |  |  | <0.001*** |
| No | 5925 | 2102 (35.5) | 1847 (31.2) | 1976 (33.4) |  |
| Yes | 134 | 20 (14.9) | 51 (38.1) | 63 (47.0) |  |
| **Household’s bed net ownership** |  |  |  |  | <0.001*** |
| No | 1780 | 516 (29.0) | 608 (34.2) | 656 (36.9) |  |
| Yes | 4279 | 1606 (37.5) | 1290 (30.1) | 1383 (32.3) |  |
| **Mother’s ethnicity** |  |  |  |  | <0.001*** |
| Hausa/Fulani | 2364 | 1354 (57.3) | 519 (22.0) | 491 (20.8) |  |
| Yoruba | 622 | 86 (13.8) | 288 (46.3) | 248 (39.9) |  |
| Igbo | 847 | 60 (7.1) | 343 (40.5) | 444 (52.4) |  |
| Others (ekoi, ibibio, etc.) | 2226 | 622 (27.9) | 748 (33.6) | 856 (38.5) |  |
| **Household wealth** |  |  |  |  | <0.001*** |
| Poorer/poorest | 2784 | 1486 (53.4) | 685 (24.6) | 613 (22.0) |  |
| Middle | 1253 | 366 (29.2) | 402 (32.1) | 485 (38.7) |  |
| Richer/richest | 2022 | 270 (13.4) | 811 (40.1) | 941 (46.5) |  |
| **Access to bank account** |  |  |  |  | <0.001*** |
| No | 5035 | 2063 (41.0) | 1450 (28.8) | 1522 (30.2) |  |
| Yes | 1024 | 59 (5.8) | 448 (43.8) | 517 (50.5) |  |
| **Household size** |  |  |  |  | <0.001*** |
| Large (>=9) | 1464 | 705 (48.2) | 389 (26.6) | 370 (25.3) |  |
| Medium (5 to 8) | 2760 | 906 (32.8) | 893 (32.4) | 961 (34.8) |  |
| Small (<=4) | 1835 | 511 (27.8) | 616 (33.6) | 708 (38.6) |  |
| **Length of stay in household** |  |  |  |  | <0.001*** |
| <1year/visitor | 174 | 27 (15.5) | 100 (57.5) | 47 (27.0) |  |
| 1-3years | 711 | 177 (24.9) | 217 (30.5) | 317 (44.6) |  |
| 4-5years | 438 | 121 (27.6) | 142 (32.4) | 175 (40.0) |  |
| >5years/always | 4736 | 1797 (37.9) | 1439 (30.4) | 1500 (31.7) |  |
| **Rural/urban** |  |  |  |  | <0.001*** |
| Rural | 3959 | 1717 (43.4) | 1094 (27.6) | 1148 (29.0) |  |
| Urban | 2100 | 405 (19.3) | 804 (38.3) | 891 (42.4) |  |
| **Livestock density index** |  |  |  |  | <0.001*** |
| Lower (0-21.4) | 1637 | 434 (26.5) | 569 (34.8) | 634 (38.7) |  |
| Medium (21.5-73.2) | 2034 | 702 (34.5) | 691 (34.0) | 641 (31.5) |  |
| Higher (73.3-5196.8) | 2365 | 981 (41.5) | 628 (26.6) | 756 (32.0) |  |
| **Travel time to the nearest health facility (providing RI services)** |  |  |  |  | <0.001*** |
| Lower (0-3.9) | 1683 | 288 (17.1) | 610 (36.2) | 785 (46.6) |  |
| Medium (4.0-12.5) | 2000 | 661 (33.1) | 658 (32.9) | 681 (34.1) |  |
| Higher (12.6-532.8) | 2353 | 1168 (49.6) | 620 (26.3) | 565 (24.0) |  |
| **Average enhanced vegetation index (2013-2018)** |  |  |  |  | <0.001*** |
| Lower (0.07-0.23) | 2395 | 1184 (49.4) | 616 (25.7) | 595 (24.8) |  |
| Medium (0.24-0.36) | 2202 | 688 (31.2) | 685 (31.1) | 829 (37.6) |  |
| Higher (0.364-0.57) | 1439 | 245 (17.0) | 587 (40.8) | 607 (42.2) |  |

*p<0.05; **p<0.01; ***p<0.001.

**Table K: Factors associated with PENTA1 in the Bayesian multilevel multinomial logistic regression model, multivariable analyses of Nigeria DHS 2018**

|  | **PENTA 1** | |
| --- | --- | --- |
|  | **Card Invalid/history** | **Card valid** |
| **Variables** | **aRR (95% CI)** | **aRR (95% CI)** |
| **Sex of child** |  |  |
| Male | 1.00 [reference] | 1.00 [reference] |
| Female | 1.02 [0.86, 1.19] | 1.08 [0.91, 1.27] |
| **Birth order** |  |  |
| 1-2 | 1.00 [reference] | 1.00 [reference] |
| >2 | 1.04 [0.82, 1.31] | 0.94 [0.72, 1.21] |
| **Birth quarter** |  |  |
| Jan-Mar | 1.28 [0.98, 1.62] | 1.49 [1.13, 1.91] ^+^ |
| Apr-Jun | 1.17 [0.90, 1.49] | 1.38 [1.03, 1.78] ^+^ |
| Jul-Sep | 1.08 [0.82, 1.39] | 1.37 [1.03, 1.78] ^+^ |
| Oct-Dec | 1.00 [reference] | 1.00 [reference] |
| **Skilled birth attendance** |  |  |
| No skilled attendant at birth | 1.00 [reference] | 1.00 [reference] |
| Skilled attendant at birth | 2.36 [1.89, 2.92] ^+^ | 2.37 [1.88, 2.97] ^+^ |
| **Received vitamin A** |  |  |
| No/don’t know | 1.00 [reference] | 1.00 [reference] |
| Yes | 3.90 [3.21, 4.68] ^+^ | 5.12 [4.17, 6.23] ^+^ |
| **Sex of household head** |  |  |
| Female | 1.00 [reference] | 1.00 [reference] |
| Male | 1.22 [0.88, 1.67] | 0.97 [0.68, 1.33] |
| **Mother's age group** |  |  |
| 15-19 | 1.00 [reference] | 1.00 [reference] |
| 20-29 | 1.27 [0.87, 1.81] | 1.02 [0.69, 1.44] |
| 30-39 | 1.18 [0.76, 1.73] | 1.08 [0.69, 1.61] |
| 40-49 | 0.79 [0.46, 1.26] | 0.80 [0.47, 1.27] |
| **Marital status of mother** |  |  |
| Never in union | 0.77 [0.43, 1.32] | 0.76 [0.41, 1.31] |
| Married | 1.00 [reference] | 1.00 [reference] |
| Divorced | 0.77 [0.43, 1.25] | 0.57 [0.31, 0.98] ^+^ |
| **Mother employed in the past 12 months** |  |  |
| No | 1.00 [reference] | 1.00 [reference] |
| Yes (currently/in the past 1 year) | 1.39 [1.15, 1.68] ^+^ | 1.23 [0.98, 1.50] |
| **Mother had problem seeking medical advice or treatment** |  |  |
| Had problem seeking medical advice or treatment | 1.00 [reference] | 1.00 [reference] |
| Did not have problem seeking medical advice or treatment | 1.37 [1.12, 1.65] ^+^ | 1.44 [1.19, 1.76] ^+^ |
| **Mother’s education** |  |  |
| No education | 1.00 [reference] | 1.00 [reference] |
| Primary | 1.47 [1.12, 1.92] ^+^ | 1.73 [1.30, 2.27] ^+^ |
| Secondary/higher | 2.02 [1.52, 2.65] ^+^ | 2.49 [1.81, 3.28] ^+^ |
| **Religion** |  |  |
| Islam | 1.00 [reference] | 1.00 [reference] |
| Christian | 1.56 [1.11, 2.11] ^+^ | 1.58 [1.08, 2.22] ^+^ |
| Traditionalist/others | 1.15 [0.40, 2.65] | 0.50 [0.12, 1.33] |
| **Mother’s media exposure** |  |  |
| No | 1.00 [reference] | 1.00 [reference] |
| Yes (radio/tv/newspaper at least once a week) | 1.11 [0.89, 1.36] | 1.09 [0.87, 1.35] |
| **Mother’s access to mobile phone/internet** |  |  |
| No | 1.00 [reference] | 1.00 [reference] |
| Yes | 1.33 [1.07, 1.62] ^+^ | 1.23 [1.00, 1.51] ^+^ |
| **Mother’s land ownership** |  |  |
| Does not own land | 1.00 [reference] | 1.00 [reference] |
| Owns land alone and/or jointly | 1.19 [0.88, 1.55] | 1.08 [0.79, 1.43] |
| **Mother’s knowledge of malaria** |  |  |
| Has no knowledge | 1.00 [reference] | 1.00 [reference] |
| Has knowledge | 1.34 [0.92, 1.87] | 0.93 [0.62, 1.32] |
| **Mother had health insurance** |  |  |
| No | 1.00 [reference] | 1.00 [reference] |
| Yes | 1.53 [0.68, 3.04] | 1.54 [0.66, 3.20] |
| **Household’s bed net ownership** |  |  |
| No | 1.00 [reference] | 1.00 [reference] |
| Yes | 1.03 [0.83, 1.26] | 1.15 [0.92, 1.42] |
| **Mother’s ethnicity** |  |  |
| Hausa/Fulani | 1.00 [reference] | 1.00 [reference] |
| Yoruba | 1.34 [0.77, 2.15] | 1.01 [0.54, 1.68] |
| Igbo | 2.30 [1.17, 4.05] ^+^ | 2.38 [1.14, 4.35] ^+^ |
| Others (ekoi, ibibio, etc.) | 1.37 [1.01, 1.80] ^+^ | 1.42 [1.02, 1.91] ^+^ |
| **Household wealth** |  |  |
| Poorer/poorest | 1.00 [reference] | 1.00 [reference] |
| Middle | 1.08 [0.83, 1.37] | 1.52 [1.15, 1.95] ^+^ |
| Richer/richest | 1.35 [0.98, 1.78] | 1.75 [1.26, 2.36] ^+^ |
| **Access to bank account** |  |  |
| No | 1.00 [reference] | 1.00 [reference] |
| Yes | 2.41 [1.65, 3.45] ^+^ | 2.15 [1.47, 3.08] ^+^ |
| **Household size** |  |  |
| Large (>=9) | 1.17 [0.93, 1.43] | 1.01 [0.80, 1.26] |
| Medium (5 to 8) | 1.00 [reference] | 1.00 [reference] |
| Small (<=4) | 1.12 [0.89, 1.41] | 1.18 [0.92, 1.47] |
| **Length of stay in household** |  |  |
| <1year/visitor | 2.78 [1.37, 5.25] ^+^ | 1.03 [0.47, 1.99] |
| 1-3years | 1.19 [0.76, 1.78] | 1.36 [0.85, 2.09] |
| 4-5years | 1.00 [reference] | 1.00 [reference] |
| >5years/always | 1.51 [1.03, 2.17] ^+^ | 1.43 [0.95, 2.06] ^+^ |
| **Rural/urban** |  |  |
| Rural | 1.00 [reference] | 1.00 [reference] |
| Urban | 0.99 [0.62, 1.49] | 0.79 [0.46, 1.25] |
| **Livestock density index** |  |  |
| Lower (0-21.4) | 1.52 [1.05, 2.15] ^+^ | 1.39 [0.92, 2.02] |
| Medium (21.5-73.2) | 1.39 [1.01, 1.85] ^+^ | 1.16 [0.81, 1.60] |
| Higher (73.3-5196.8) | 1.00 [reference] | 1.00 [reference] |
| **Travel time to the nearest health facility (providing RI services)** |  |  |
| Lower (0-3.9) | 1.36 [0.85, 2.08] | 2.38 [1.40, 3.76] ^+^ |
| Medium (4.0-12.5) | 1.51 [1.14, 1.97] ^+^ | 1.97 [1.42, 2.69] ^+^ |
| Higher (12.6-532.8) | 1.00 [reference] | 1.00 [reference] |
| **Average enhanced vegetation index (2013-2018)** |  |  |
| Lower (0.07-0.23) | 1.00 [reference] | 1.00 [reference] |
| Medium (0.24-0.36) | 1.36 [1.00, 1.81] ^+^ | 1.60 [1.13, 2.21] ^+^ |
| Higher (0.364-0.57) | 1.26 [0.80, 1.84] | 1.36 [0.83, 2.05] |
|  | | |
| **Variance parameter estimates** | **Posterior mean [95% Cr.I]** | |
| Stratum level |  | |
| $\hat{\sigma}_{s(1)}^{2}$ (variance for card invalid/history) | 0.39 [0.21, 0.66] | |
| $\hat{\sigma}_{s(2)}^{2}$ (variance for card valid) | 0.57 [0.32, 0.93] | |
| $\hat{\sigma}_{s(1,2)}^{2}$ (covariance between card invalid/history and card valid) | 0.35 [0.16, 0.62] | |
| Cluster level |  | |
| $\hat{\sigma}_{c(1)}^{2}$ (variance for card invalid/history) | 0.73 [0.47, 1.03] | |
| $\hat{\sigma}_{c(2)}^{2}$ (variance for card valid) | 1.24 [0.90, 1.63] | |
| $\hat{\sigma}_{c(1,2)}^{2}$ (covariance between card invalid/history and card valid) | 0.68 [0.42, 0.95] | |

aRR: adjusted relative risk; Cr.I: Bayesian Credible Interval; ^+^Significant covariate for the Bayesian model


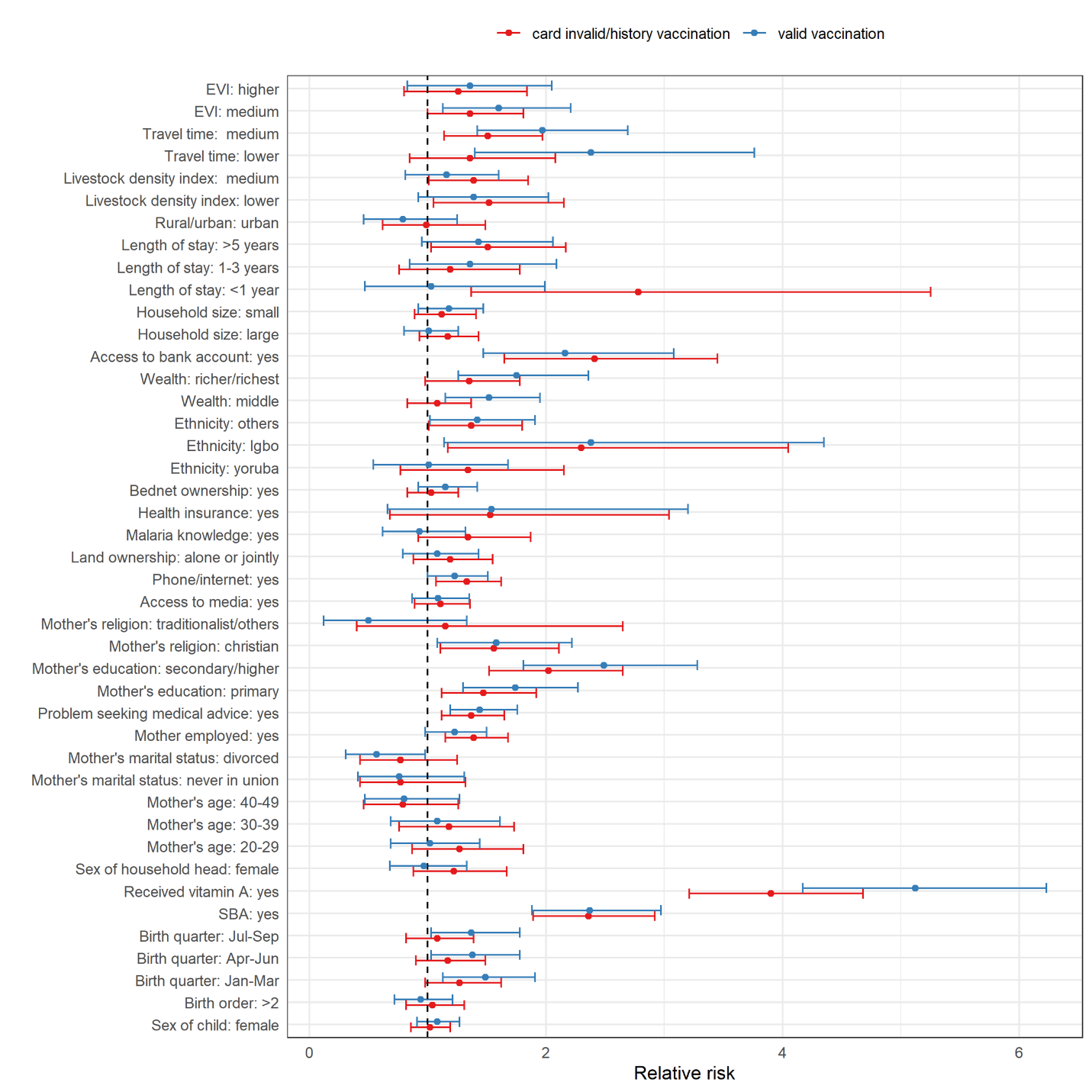
**Fig D. Plots of relative risks and corresponding 95% credible interval based on Bayesian multilevel multinomial analysis for PENTA1 vaccination coverage. The vertical dotted line marks the relative risk of 1.**

**Interpretation of relative risk estimates for PENTA1** **in the multinomial analysis**

Presented in Table K and Fig D are the Bayesian multiple multilevel multinomial results for PENTA1. Birth quarter (Jan-Mar: aRR=1.49, 95% Cr.I: 1.13, 1.91; Apr-Jun: aRR=1.38, 95% Cr.I: 1.03, 1.78; Jul-Sep: aRR=1.37, 95% Cr.I: 1.03, 1.78) was significantly associated with higher likelihood for receipt of card valid vaccination relative to no evidence of vaccination for PENTA1, but not for card invalid/history, while skilled attendance at birth was associated with increased likelihood for receipt of both card invalid/history (aRR=2.36, 95% Cr.I: 1.89, 2.92) and card valid (aRR=2.37, 95% Cr.I: 1.88, 2.97) vaccinations relative to no evidence of vaccination. Similarly, receipt of vitamin A was associated with higher likelihood for both card invalid/history (aRR=3.90, 95% Cr.I: 3.21, 4.68) and card valid (aRR=5.12, 95% Cr.I: 4.17, 6.23) vaccinations relative to no evidence of vaccination. Belonging to a divorced mother (aRR=0.57, 95% Cr.I: 0.31, 0.98) was predictive of card valid vaccination only, and having a mother who is employed (aRR=1.39, 95% Cr.I: 1.15, 1.68) was predictive of card invalid/history vaccination only. Having a mother who had problem seeking medical advice or treatment is associated with both card invalid/history (aRR=1.37, 95% Cr.I: 1.12, 1.65) and card valid (aRR=1.44, 95% Cr.I: 1.19, 1.76) vaccination. Maternal education was associated with both card invalid/history (primary: aRR=1.47, 95% Cr.I: 1.12, 1.92; secondary/higher: aRR=2.02, 95% Cr.I: 1.52, 2.65) and card valid (primary: aRR=1.73, 95% Cr.I: 1.30, 2.27; secondary/higher: aRR=2.49, 95% Cr.I: 1.81, 3.28) vaccination.

Having a Christian mother was associated with higher likelihood for receipt of both card invalid/history (aRR=1.56, 95% Cr.I: 1.11, 2.11) and card valid (aRR=1.58, 95% Cr.I: 1.08, 2.22) vaccinations. Similarly, mother’s access to mobile phone/internet was associated with increased likelihood of both card invalid/history (aRR=1.33, 95% Cr.I: 1.07, 1.62) and card valid (aRR=1.23, 95% Cr.I: 1.00, 1.51) vaccinations. Igbo ethnic group was predictive of both card invalid/history (aRR=2.30, 95% Cr.I: 1.17, 4.05) and valid card (aRR=2.38, 95% Cr.I: 1.14, 4.35) vaccination while other ethnic groups (e.g., Ekoi, Ibibio) was predictive of both card invalid/history (aRR=1.37, 95% Cr.I: 1.01, 1.80]) and card valid (aRR=1.42, 95% Cr.I: 1.02, 1.91) vaccinations. Household wealth (middle: aRR=1.52, 95% Cr.I: 1.15, 1.95; and richest: aRR=1.75, 95% Cr.I: 1.26, 2.36) was only predictive of card valid vaccination. Having a mother who had access to bank account was predictive of both card invalid/history (aRR=2.41, 95% Cr.I: 1.65, 3.45) and card valid (aRR=2.15, 95% Cr.I: 1.47, 3.08) vaccination.

Furthermore, residing in household for less than one year (aRR=2.78, 95% Cr.I: 1.37, 5.25) was predictive of card invalid/history vaccination only but residing in household for more than 5 years or always was predictive of both card invalid/history (aRR=1.51, 95% Cr.I: 1.03, 2.17) and card valid (aRR=1.43, 95% Cr.I: 0.95, 2.06) vaccinations. Livestock index (lower: aRR=1.52, 95% Cr.I: 1.05, 2.15, and medium: aRR=1.39, 95% Cr.I: 1.01, 1.85) was associated with increased likelihood of receipt of card invalid/history vaccination only. Residing within lower travel times (aRR=2.38, 95% Cr.I: 1.40, 3.76) to the nearest health facility was predictive of card valid vaccination only while residing in medium travel times to health facility was predictive of both card invalid/history (aRR=1.51, 95% Cr.I: 1.14, 1.97) and card valid (aRR=1.97, 95% Cr.I: 1.42, 2.69) vaccination. Residing in communities with medium enhanced vegetation index was predictive of both card invalid/history (aRR=1.36, 95% Cr.I: 1.00, 1.81) and card valid (aRR=1.60, 95% Cr.I: 1.13, 2.21) vaccination (Table K).

**MV**

**Table L: Factors associated with MV vaccination in the multinomial cross-tabulation analyses of Nigeria DHS 2018**

|  |  | **MV** | | |  |
| --- | --- | --- | --- | --- | --- |
| **Variables** |  | **No evidence of vaccination** | **Card invalid/ history** | **Card valid** | **P-value** |
|  | **N** | **n (%)** | **n (%)** | **n (%)** |  |
| N | 11839 | 5206(44.0) | 4522(38.2) | 2111(17.8) |  |
| **Sex of child** |  |  |  |  | 0.720 |
| Male | 6050 | 2682 (44.3) | 2299 (38.0) | 1069 (17.7) |  |
| Female | 5789 | 2524 (43.6) | 2223 (38.4) | 1042 (18.0) |  |
| **Birth order** |  |  |  |  | <0.001*** |
| 1-2 | 4485 | 1769 (39.4) | 1760 (39.2) | 956 (21.3) |  |
| >2 | 7354 | 3437 (46.7) | 2762 (37.6) | 1155 (15.7) |  |
| **Birth quarter** |  |  |  |  | <0.001*** |
| Jan-Mar | 3801 | 1611 (42.4) | 1490 (39.2) | 700 (18.4) |  |
| Apr-Jun | 3237 | 1471 (45.4) | 1203 (37.2) | 563 (17.4) |  |
| Jul-Sep | 3194 | 1478 (46.3) | 1162 (36.4) | 554 (17.3) |  |
| Oct-Dec | 1607 | 646 (40.2) | 667 (41.5) | 294 (18.3) |  |
| **Skilled birth attendance** |  |  |  |  | <0.001*** |
| No skilled attendant at birth | 6509 | 3887 (59.7) | 1979 (30.4) | 643 (9.9) |  |
| Skilled attendant at birth | 5330 | 1319 (24.7) | 2543 (47.7) | 1468 (27.5) |  |
| **Health card/document** |  |  |  |  | <0.001*** |
| Does not have health card/document | 6600 | 3884 (58.8) | 2716 (41.2) | 0 (0.0) |  |
| Yes, none seen | 1104 | 231 (20.9) | 873 (79.1) | 0 (0.0) |  |
| Yes, seen | 4135 | 1091 (26.4) | 933 (22.6) | 2111 (51.1) |  |
| **Received vitamin A** |  |  |  |  | <0.001*** |
| No/don’t know | 5658 | 3595 (63.5) | 1626 (28.7) | 437 (7.7) |  |
| Yes | 6181 | 1611 (26.1) | 2896 (46.9) | 1674 (27.1) |  |
| **Sex of household head** |  |  |  |  | <0.001*** |
| Female | 10662 | 4826 (45.3) | 3947 (37.0) | 1889 (17.7) |  |
| Male | 1177 | 380 (32.3) | 575 (48.9) | 222 (18.9) |  |
| **Mother's age group** |  |  |  |  | <0.001*** |
| 15-19 | 587 | 370 (63.0) | 151 (25.7) | 66 (11.2) |  |
| 20-29 | 5766 | 2617 (45.4) | 2157 (37.4) | 992 (17.2) |  |
| 30-39 | 4508 | 1748 (38.8) | 1861 (41.3) | 899 (19.9) |  |
| 40-49 | 978 | 471 (48.2) | 353 (36.1) | 154 (15.7) |  |
| **Marital status of mother** |  |  |  |  | <0.001*** |
| Never in union | 287 | 96 (33.4) | 129 (44.9) | 62 (21.6) |  |
| Married | 11222 | 4982 (44.4) | 4242 (37.8) | 1998 (17.8) |  |
| Divorced | 330 | 128 (38.8) | 151 (45.8) | 51 (15.5) |  |
| **Mother employed in the past 12 months** |  |  |  |  | <0.001*** |
| No | 3403 | 1926 (56.6) | 1040 (30.6) | 437 (12.8) |  |
| Yes (currently/in the past 1 year) | 8436 | 3280 (38.9) | 3482 (41.3) | 1674 (19.8) |  |
| **Mother had problem seeking medical advice or treatment** |  |  |  |  | <0.001*** |
| Had problem seeking medical advice or treatment | 6529 | 3256 (49.9) | 2287 (35.0) | 986 (15.1) |  |
| Did not have problem seeking medical advice or treatment | 5310 | 1950 (36.7) | 2235 (42.1) | 1125 (21.2) |  |
| **Mother’s education** |  |  |  |  | <0.001*** |
| No education | 5112 | 3262 (63.8) | 1479 (28.9) | 371 (7.3) |  |
| Primary | 1784 | 770 (43.2) | 688 (38.6) | 326 (18.3) |  |
| Secondary/higher | 4943 | 1174 (23.8) | 2355 (47.6) | 1414 (28.6) |  |
| **Religion** |  |  |  |  | <0.001*** |
| Islam | 6901 | 3849 (55.8) | 2289 (33.2) | 763 (11.1) |  |
| Christian | 4835 | 1321 (27.3) | 2172 (44.9) | 1342 (27.8) |  |
| Traditionalist/others | 103 | 36 (35.0) | 61 (59.2) | 6 (5.8) |  |
| **Mother’s media exposure** |  |  |  |  | <0.001*** |
| No | 7247 | 3868 (53.4) | 2414 (33.3) | 965 (13.3) |  |
| Yes (radio/tv/newspaper at least once a week) | 4592 | 1338 (29.1) | 2108 (45.9) | 1146 (25.0) |  |
| **Mother’s access to mobile phone/internet** |  |  |  |  | <0.001*** |
| No | 5867 | 3424 (58.4) | 1805 (30.8) | 638 (10.9) |  |
| Yes | 5972 | 1782 (29.8) | 2717 (45.5) | 1473 (24.7) |  |
| **Mother’s land ownership** |  |  |  |  | <0.001*** |
| Does not own land | 10247 | 4671 (45.6) | 3806 (37.1) | 1770 (17.3) |  |
| Owns land alone and/or jointly | 1592 | 535 (33.6) | 716 (45.0) | 341 (21.4) |  |
| **Mother’s knowledge of malaria** |  |  |  |  | <0.001*** |
| Has no knowledge | 685 | 347 (50.7) | 208 (30.4) | 130 (19.0) |  |
| Has knowledge | 11154 | 4859 (43.6) | 4314 (38.7) | 1981 (17.8) |  |
| **Mother had health insurance** |  |  |  |  | <0.001*** |
| No | 11578 | 5151 (44.5) | 4392 (37.9) | 2035 (17.6) |  |
| Yes | 261 | 55 (21.1) | 130 (49.8) | 76 (29.1) |  |
| **Household’s bed net ownership** |  |  |  |  | <0.001*** |
| No | 3543 | 1432 (40.4) | 1392 (39.3) | 719 (20.3) |  |
| Yes | 8296 | 3774 (45.5) | 3130 (37.7) | 1392 (16.8) |  |
| **Mother’s ethnicity** |  |  |  |  | <0.001*** |
| Hausa/Fulani | 4728 | 2899 (61.3) | 1464 (31.0) | 365 (7.7) |  |
| Yoruba | 1201 | 294 (24.5) | 600 (50.0) | 307 (25.6) |  |
| Igbo | 1667 | 367 (22.0) | 755 (45.3) | 545 (32.7) |  |
| Others (ekoi, ibibio, etc.) | 4243 | 1646 (38.8) | 1703 (40.1) | 894 (21.1) |  |
| **Household wealth** |  |  |  |  | <0.001*** |
| Poorer/poorest | 5353 | 3205 (59.9) | 1666 (31.1) | 482 (9.0) |  |
| Middle | 2478 | 1039 (41.9) | 981 (39.6) | 458 (18.5) |  |
| Richer/richest | 4008 | 962 (24.0) | 1875 (46.8) | 1171 (29.2) |  |
| **Access to bank account** |  |  |  |  | <0.001*** |
| No | 9844 | 4923 (50.0) | 3516 (35.7) | 1405 (14.3) |  |
| Yes | 1995 | 283 (14.2) | 1006 (50.4) | 706 (35.4) |  |
| **Household size** |  |  |  |  | <0.001*** |
| Large (>=9) | 2946 | 1599 (54.3) | 1012 (34.4) | 335 (11.4) |  |
| Medium (5 to 8) | 5518 | 2287 (41.4) | 2200 (39.9) | 1031 (18.7) |  |
| Small (<=4) | 3375 | 1320 (39.1) | 1310 (38.8) | 745 (22.1) |  |
| **Length of stay in household** |  |  |  |  | <0.001*** |
| <1year/visitor | 345 | 106 (30.7) | 190 (55.1) | 49 (14.2) |  |
| 1-3years | 1205 | 413 (34.3) | 469 (38.9) | 323 (26.8) |  |
| 4-5years | 864 | 315 (36.5) | 340 (39.4) | 209 (24.2) |  |
| >5years/always | 9425 | 4372 (46.4) | 3523 (37.4) | 1530 (16.2) |  |
| **Rural/urban** |  |  |  |  | <0.001*** |
| Rural | 7622 | 3908 (51.3) | 2666 (35.0) | 1048 (13.7) |  |
| Urban | 4217 | 1298 (30.8) | 1856 (44.0) | 1063 (25.2) |  |
| **Livestock density index** |  |  |  |  | <0.001*** |
| Lower (0-21.4) | 3189 | 1096 (34.4) | 1356 (42.5) | 737 (23.1) |  |
| Medium (21.5-73.2) | 4029 | 1830 (45.4) | 1534 (38.1) | 665 (16.5) |  |
| Higher (73.3-5196.8) | 4621 | 2280 (49.3) | 1632 (35.3) | 709 (15.3) |  |
| **Travel time to the nearest health facility (providing RI services)** |  |  |  |  | <0.001*** |
| Lower (0-3.9) | 3406 | 995 (29.2) | 1477 (43.4) | 934 (27.4) |  |
| Medium (4.0-12.5) | 3968 | 1713 (43.2) | 1576 (39.7) | 679 (17.1) |  |
| Higher (12.6-532.8) | 4465 | 2498 (55.9) | 1469 (32.9) | 498 (11.2) |  |

*p<0.05; **p<0.01; ***p<0.001.

**Table M: Factors associated with MV in the Bayesian multilevel multinomial logistic regression model, multivariable analyses of Nigeria DHS 2018**

|  | **MV** | |
| --- | --- | --- |
|  | **Card Invalid/history** | **Card valid** |
| **Variables** | **aRR (95% Cr.I)** | **aRR (95% Cr.I)** |
| **Sex of child** |  |  |
| Male | 1.00 [reference] | 1.00 [reference] |
| Female | 1.02 [0.93, 1.13] | 1.04 [0.91, 1.18] |
| **Birth order** |  |  |
| 1-2 | 1.00 [reference] | 1.00 [reference] |
| >2 | 0.83 [0.72, 0.97] ^+^ | 0.82 [0.69, 0.98] ^+^ |
| **Birth quarter** |  |  |
| Jan-Mar | 0.96 [0.82, 1.13] | 1.09 [0.88, 1.33] |
| Apr-Jun | 0.87 [0.74, 1.01] | 0.98 [0.79, 1.20] |
| Jul-Sep | 0.87 [0.73, 1.02] | 0.98 [0.79, 1.21] |
| Oct-Dec | 1.00 [reference] | 1.00 [reference] |
| **Skilled birth attendance** |  |  |
| No skilled attendant at birth | 1.00 [reference] | 1.00 [reference] |
| Skilled attendant at birth | 1.76 [1.54, 2.00] ^+^ | 1.86 [1.57, 2.20] ^+^ |
| **Received vitamin A** |  |  |
| No/don’t know | 1.00 [reference] | 1.00 [reference] |
| Yes | 3.18 [2.83, 3.55] ^+^ | 5.66 [4.85, 6.58] ^+^ |
| **Sex of household head** |  |  |
| Female | 1.00 [reference] | 1.00 [reference] |
| Male | 1.15 [0.96, 1.38] | 0.81 [0.64, 1.01] |
| **Mother's age group** |  |  |
| 15-19 | 1.00 [reference] | 1.00 [reference] |
| 20-29 | 1.58 [1.22, 2.00] ^+^ | 1.46 [1.02, 2.06] ^+^ |
| 30-39 | 1.87 [1.40, 2.42] ^+^ | 1.77 [1.19, 2.54] ^+^ |
| 40-49 | 1.52 [1.08, 2.05] ^+^ | 1.58 [0.98, 2.38] |
| **Marital status of mother** |  |  |
| Never in union | 1.07 [0.75, 1.47] | 1.01 [0.64, 1.49] |
| Married | 1.00 [reference] | 1.00 [reference] |
| Divorced | 1.17 [0.85, 1.56] | 0.92 [0.59, 1.34] |
| **Mother employed in the past 12 months** |  |  |
| No | 1.00 [reference] | 1.00 [reference] |
| Yes (currently/in the past 1 year) | 1.38 [1.21, 1.55] ^+^ | 1.36 [1.16, 1.58] ^+^ |
| **Mother had problem seeking medical advice or treatment** |  |  |
| Had problem seeking medical advice or treatment | 1.00 [reference] | 1.00 [reference] |
| Did not have problem seeking medical advice or treatment | 1.20 [1.07, 1.34] ^+^ | 1.24 [1.07, 1.42] ^+^ |
| **Mother’s education** |  |  |
| No education | 1.00 [reference] | 1.00 [reference] |
| Primary | 1.19 [1.01, 1.39] ^+^ | 1.52 [1.21, 1.88] ^+^ |
| Secondary/higher | 1.52 [1.29, 1.78] ^+^ | 1.95 [1.57, 2.39] ^+^ |
| **Religion** |  |  |
| Islam | 1.00 [reference] | 1.00 [reference] |
| Christian | 1.17 [0.96, 1.43] | 1.24 [0.97, 1.55] |
| Traditionalist/others | 1.73 [0.94, 2.99] | 0.58 [0.18, 1.39] |
| **Mother’s media exposure** |  |  |
| No | 1.00 [reference] | 1.00 [reference] |
| Yes (radio/tv/newspaper at least once a week) | 1.22 [1.08, 1.37] ^+^ | 1.26 [1.08, 1.46] ^+^ |
| **Mother’s access to mobile phone/internet** |  |  |
| No | 1.00 [reference] | 1.00 [reference] |
| Yes | 1.26 [1.11, 1.42] ^+^ | 1.13 [0.96, 1.32] |
| **Mother’s land ownership** |  |  |
| Does not own land | 1.00 [reference] | 1.00 [reference] |
| Owns land alone and/or jointly | 1.24 [1.05, 1.45] ^+^ | 0.94 [0.76, 1.14] |
| **Mother’s knowledge of malaria** |  |  |
| Has no knowledge | 1.00 [reference] | 1.00 [reference] |
| Has knowledge | 1.18 [0.92, 1.48] | 0.95 [0.70, 1.23] |
| **Mother had health insurance** |  |  |
| No | 1.00 [reference] | 1.00 [reference] |
| Yes | 1.57 [1.00, 2.37] ^+^ | 1.33 [0.78, 2.12] |
| **Household’s bed net ownership** |  |  |
| No | 1.00 [reference] | 1.00 [reference] |
| Yes | 1.13 [1.00, 1.27] ^+^ | 1.26 [1.08, 1.45] ^+^ |
| **Mother’s ethnicity** |  |  |
| Hausa/Fulani | 1.00 [reference] | 1.00 [reference] |
| Yoruba | 1.30 [0.91, 1.79] | 1.24 [0.83, 1.82] |
| Igbo | 1.53 [1.04, 2.21] ^+^ | 2.05 [1.35, 3.10] ^+^ |
| Others (ekoi, ibibio, etc.) | 1.20 [0.99, 1.45] | 1.56 [1.21, 1.98] ^+^ |
| **Household wealth** |  |  |
| Poorer/poorest | 1.00 [reference] | 1.00 [reference] |
| Middle | 1.07 [0.92, 1.23] | 1.39 [1.12, 1.70] ^+^ |
| Richer/richest | 1.30 [1.09, 1.55] ^+^ | 1.79 [1.40, 2.24] ^+^ |
| **Access to bank account** |  |  |
| No | 1.00 [reference] | 1.00 [reference] |
| Yes | 1.74 [1.45, 2.09] ^+^ | 2.08 [1.69, 2.55] ^+^ |
| **Household size** |  |  |
| Large (>=9) | 0.98 [0.86, 1.11] | 0.89 [0.73, 1.07] |
| Medium (5 to 8) | 1.00 [reference] | 1.00 [reference] |
| Small (<=4) | 0.92 [0.80, 1.05] | 1.11 [0.93, 1.31] |
| **Length of stay in household** |  |  |
| <1year/visitor | 1.57 [1.09, 2.21] ^+^ | 0.60 [0.36, 0.92] ^+^ |
| 1-3years | 1.10 [0.84, 1.40] | 1.13 [0.83, 1.52] |
| 4-5years | 1.00 [reference] | 1.00 [reference] |
| >5years/always | 1.16 [0.93, 1.42] | 1.09 [0.84, 1.38] |
| **Rural/urban** |  |  |
| Rural | 1.00 [reference] | 1.00 [reference] |
| Urban | 1.01 [0.70, 1.44] | 0.86 [0.57, 1.20] |
| **Livestock density index** |  |  |
| Lower (0-21.4) | 1.62 [1.26, 2.05] ^+^ | 1.57 [1.16, 2.11] ^+^ |
| Medium (21.5-73.2) | 1.16 [0.94, 1.40] | 1.11 [0.85, 1.42] |
| Higher (73.3-5196.8) | 1.00 [reference] | 1.00 [reference] |
| **Travel time to the nearest health facility (providing RI services)** |  |  |
| Lower (0-3.9) | 1.05 [0.79, 1.37] | 1.50 [1.05, 2.08] ^+^ |
| Medium (4.0-12.5) | 1.21 [1.00, 1.44] ^+^ | 1.48 [1.13, 1.86] ^+^ |
| Higher (12.6-532.8) | 1.00 [reference] | 1.00 [reference] |
|  | | |
| **Variance parameter estimates** | **Posterior mean [95% Cr.I]** | |
| **Stratum level** |  | |
| $\hat{\sigma}_{s(1)}^{2}$ (variance for card invalid/history) | 0.45 [0.29, 0.67] | |
| $\hat{\sigma}_{s(2)}^{2}$ (variance for card valid) | 0.35 [0.20, 0.56] | |
| $\hat{\sigma}_{s(1,2)}^{2}$ (covariance between card invalid/history and card valid) | 0.25 [0.12, 0.43] | |
| **Cluster level** |  | |
| $\hat{\sigma}_{c(1)}^{2}$ (variance for card invalid/history) | 0.43 [0.33, 0.54] | |
| $\hat{\sigma}_{c(2)}^{2}$ (variance for card valid) | 0.76 [0.59, 0.96] | |
| $\hat{\sigma}_{c(1,2)}^{2}$ (covariance between card invalid/history and card valid) | 0.36 [0.26, 0.48] | |

aRR: adjusted relative risk; Cr.I: Bayesian Credible Interval; ^+^Significant covariate for the Bayesian model

**Interpretation of relative risk estimates for MV in the multinomial analysis**

Table M presents the results from the Bayesian multiple multilevel multinomial analysis for MV. Receipt of assistance from a SBA was associated with higher likelihood for receipt of both card invalid/history (aRR=1.76, 95% Cr.I: 1.54, 2.00) and card valid (aRR=1.86, 95% Cr.I: 1.57, 2.20) vaccinations for MV, while receipt of vitamin A was predictive of higher likelihood of receipt of both card invalid/history (aRR=3.18, 95% Cr.I: 2.83, 3.55) and card valid (aRR=5.66, 95% Cr.I: 4.85, 6.58) vaccinations for MV. Similarly, maternal age was associated with higher likelihood of receipt of both card invalid/history (20-29: aRR=1.58, 95% Cr.I: 1.22, 2.00, 30-39: aRR=1.87, 95% Cr.I: 1.40, 2.42, and 40-49: aRR=1.52, 95% Cr.I: 1.08, 2.05) and card valid (20-29: aRR=1.46, 95% Cr.I: 1.02, 2.06, 30-39: aRR=1.77, 95% Cr.I: 1.19, 2.54, but not for age 40-49) vaccinations for MV. Maternal employment was associated with increased likelihood of receipt of both card invalid/history (aRR=1.38, 95% Cr.I: 1.21, 1.55) and card valid (aRR=1.36, 95% Cr.I: 1.16, 1.58) for MV vaccinations, while belonging to mothers who had problem seeking medical advice or treatment was associated with significantly higher likelihood for receipt of card invalid/history (aRR=1.20, 95% Cr.I: 1.07, 1.34) and card valid (aRR=1.24, 95% Cr.I: 1.07, 1.42) vaccinations for MV. Maternal education was predictive of higher likelihood of receipt of both card invalid/history (primary: aRR=1.19, 95% Cr.I: 1.01, 1.39, secondary: aRR=1.52, 95% Cr.I: 1.29, 1.78) and card valid history (primary: aRR=1.52, 95% Cr.I: 1.21, 1.88, secondary: aRR=1.95, 95% Cr.I: 1.57, 2.39) vaccinations for MV. Having a mother who was exposed to the media was predictive of increased likelihood of receipt of both card invalid/history (aRR=1.22, 95% Cr.I: 1.08, 1.37) and card valid (aRR=1.26, 95% Cr.I: 1.08, 1.46) MV receipt, while access to phone/internet (aRR=1.26, 95% Cr.I: 1.11, 1.42), mothers who owned land (aRR=1.24, 95% Cr.I: 1.05, 1.45), health insurance (aRR=1.57, 95% Cr.I: 1.00, 2.37) were significant predictors of higher likelihood of receipt of card invalid/history of MV but not for card valid. Children from households that owned bed net was associated with higher odds of receipt of both card invalid/history (aRR=1.13, 95% Cr.I: 1.00, 1.27) and card valid (aRR=1.26, 95% Cr.I: 1.08, 1.45) MV receipt. Ethnicity was associated with increased likelihood of receipt of both card invalid/history (Igbo: aRR=1.53, 95% Cr.I: 1.04, 2.21, but not others) and card valid (Igbo: aRR=2.05, 95% Cr.I: 1.35, 3.10, Others (Ekoi, Ibibio, etc): aRR=1.56, 95% Cr.I: 1.21, 1.98) MV. Wealth was associated with increased likelihood of receipt of both card invalid/history (richer/richest: aRR=1.30, 95% Cr.I: 1.09, 1.55, but not for middle category) and card valid (middle: aRR=1.39, 95% Cr.I: 1.12, 1.70, richer/richest: aRR=1.79, 95% Cr.I: 1.40, 2.24) MV. Having mothers who had access to bank accounts was predictive of increased likelihood of receipt of both card invalid/history (aRR=1.74, 95% Cr.I: 1.45, 2.09) and card valid (aRR=2.08, 95% Cr.I: 1.69, 2.55) MV. Staying in a household for less than one (1) year was predictive of higher likelihood of receipt of card invalid/history (aRR=1.57, 95% Cr.I: 1.09, 2.21), but was predictive of reduced likelihood of receipt of card valid (aRR=0.60, 95% Cr.I: 0.36, 0.92) MV. Higher birth order was associated with reduced likelihood for receipt of both invalid/history (aRR=0.83, 95% Cr.I: .72, 0.97) and card valid (aRR=0.82, 95% Cr.I: 0.69, 0.98) MV. Finally, residing in communities with lower livestock density index was predictive of increased likelihood of receipt of both card invalid/history (aRR=1.62, 95% Cr.I: 1.26, 2.05) and card valid (aRR=1.57, 95% Cr.I: 1.16, 2.11) MV, while lower travel time was predictive of higher odds of receipt of card valid (aRR=1.50, 95% Cr.I: 1.05, 2.08) MV only. Medium travel time was associated with higher likelihood of receipt of both card invalid/history (aRR=1.21, 95% Cr.I: 1.00, 1.44) and card valid (aRR=1.48, 95% Cr.I: 1.13, 1.86) MV (Table M).

**References**

1. Utazi CE, Thorley J, Alegana VA, Ferrari MJ, Takahashi S, Metcalf CJE, et al. High resolution age-structured mapping of childhood vaccination coverage in low and middle income countries. Vaccine. 2018;36(12):1583-91. Epub 2018/02/14. doi: 10.1016/j.vaccine.2018.02.020. PubMed PMID: 29454519; PubMed Central PMCID: PMCPMC6344781.

2. de la Fuente J, Caballero FF, Verdes E, Rodríguez-Artalejo F, Cabello M, de la Torre-Luque A, et al. Are younger cohorts in the USA and England ageing better? International Journal of Epidemiology. 2019;48(6):1906-13. doi: 10.1093/ije/dyz126.

3. Rozi DS, Mahmud DS, Lancaster DG. Multilevel Analysis to Identify the Risk Factors for Smoking on Teenage Children Attending School. International Journal of Epidemiology. 2015;44(suppl_1):i50-i. doi: 10.1093/ije/dyv097.191.

4. Hedeker D, Gibbons RD. MIXOR: a computer program for mixed-effects ordinal regression analysis. Comput Methods Programs Biomed. 1996;49(2):157-76. PubMed PMID: 8735023.

5. Aheto JMK, Udofia EA, Kallson E, Mensah G, Nadia M, Nirmala N, et al. Prevalence, socio-demographic and environmental determinants of asthma in 4621 Ghanaian adults: Evidence from Wave 2 of the World Health Organization’s study on global AGEing and adult health. PLOS ONE. 2020;15(12):e0243642. doi: 10.1371/journal.pone.0243642.

6. Kibret KT, Chojenta C, D’Arcy E, Loxton D. Spatial distribution and determinant factors of anaemia among women of reproductive age in Ethiopia: a multilevel and spatial analysis. BMJ Open. 2019;9(4):e027276. doi: 10.1136/bmjopen-2018-027276.

7. Merlo J, Chaix B, Ohlsson H, Beckman A, Johnell K, Hjerpe P, et al. A brief conceptual tutorial of multilevel analysis in social epidemiology: using measures of clustering in multilevel logistic regression to investigate contextual phenomena. J Epidemiol Community Health. 2006;60(4):290-7. doi: 10.1136/jech.2004.029454. PubMed PMID: 16537344; PubMed Central PMCID: PMCPMC2566165.

8. Leyland AH, Groenewegen PP. Multilevel Modelling for Public Health and Health Services Research: Health in Context. 2020.

9. Goldstein H. Multilevel Statistical Models. 3rd ed. London: Arnold; 2003.

10. Goldstein H, Browne W, Rasbash J. Partitioning Variation in Multilevel Models. Understanding Statistics. 2002;1(4):223-31. doi: 10.1207/S15328031US0104_02.

11. Larsen K, Petersen JH, Budtz-Jørgensen E, Endahl L. Interpreting parameters in the logistic regression model with random effects. Biometrics. 2000;56(3):909-14. Epub 2000/09/14. doi: 10.1111/j.0006-341x.2000.00909.x. PubMed PMID: 10985236.

12. Larsen K, Merlo J. Appropriate assessment of neighborhood effects on individual health: integrating random and fixed effects in multilevel logistic regression. Am J Epidemiol. 2005;161(1):81-8. Epub 2004/12/24. doi: 10.1093/aje/kwi017. PubMed PMID: 15615918.

13. Leckie G, Charlton C. runmlwin - A Program to Run the MLwiN Multilevel Modelling Software from within Stata. Journal of Statistical Software. 2013;52(11):1-40.

14. Cameron A, Trivedi P. Multinomial Models. In: Cameron AC, Trivedi PK, editors. Microeconometrics: Methods and Applications. Cambridge: Cambridge University Press; 2005. p. 490-528.

15. Aheto JMK, Taylor BM, Keegan TJ, Diggle PJ. Modelling and forecasting spatio-temporal variation in the risk of chronic malnutrition among under-five children in Ghana. Spat Spatiotemporal Epidemiol. 2017;21:37-46. Epub 2017/03/02. doi: 10.1016/j.sste.2017.02.003. PubMed PMID: 28552186.

16. Fox J, Monette G. Generalized collinearity diagnostics. JASA. 1992;**87**  178–83.

17. Hair JFJ, Anderson RE, Tatham RL, Black WC. Multivariate Data Analysis  3ed. New York: Macmillan; 1995.

18. Clark PC. The Effects of Multicollinearity in Multilevel Models: Wright State University; 2013.

19. Chen JJ. Communicating complex information: the interpretation of statistical interaction in multiple logistic regression analysis. American journal of public health. 2003;93(9):1376-7. doi: 10.2105/ajph.93.9.1376-a. PubMed PMID: 12948938.

20. Hosmer DW, Lemeshow S, Sturdivant RX. Applied logistic regression2013.
