# Supplementary material for: Multilevel analysis of predictors of multiple indicators of childhood vaccination in Nigeria
Source: PLoS One. 2022 May 25;17(5):e0269066. doi: 10.1371/journal.pone.0269066 (PMC9132327; doi:10.1371/journal.pone.0269066)
Supplement: Attachment — Submitted filename: PONE-D-21-28231 comment.pdf [file pone.0269066.s002.pdf]

Review of PLUS ONE
PONE-D-21-28231: " Multilevel analysis of predictors of multiple indicators of childhood vaccination in Nigeria "

The manuscript presents a reasonably well-articulated discussion for the developed framework to address childhood vaccination inequalities in Nigeria. Based on my reading, the applications of multilevel logistic models to produce new insights to data analysis makes the current manuscript contributing to the literature of public health. The work will be of also interest to a wide readership in the journal. Although the manuscript is worth published, its present form needs some revisions and I have the following suggestions.

1. In the Introduction, the authors omitted the brief description about multilevel models. Some details can be found at supplementary file but I think the associated discussions could be summarized into the Introduction. In so doing, the article would be more complete and clear.

2. In the Data section, the authors may provide a figure for the hieratical structure of the data, which can furnish the rationale why to use multilevel models.

   On the other hand, for readability and the ease of understanding, the authors should at least include some model equations for the multilevel models in the section of Data analysis, rather than just assemble all the materials in the supplementary.

3. Is there any specific reason to use Bayesian multilevel models? As the 'frequentist' multilevel logistic regression models exist as alternative ways to analyze the data, I suggest the authors explain a bit what to motivate the Bayesian multilevel analyses and why to use them both in the Introduction and Data analysis sections.

4. Regarding the model specifications, do the multilevel models only include random intercepts? Is there any random coefficients for the predictors? From the model equation 2 and equation 3 in the supplementary file, it is not clear to me how the multilevel representation is given.

5. The authors indicated in the supplementary that non-informative priors can be alternative choice of priors for the model parameters. I was actually thinking that would it be possible to generate better analysis results as there is lack of information on reliable priors. Although I don't demand the authors to do so, the discussion needs to be added.

6. Lines 168-174: It is not clear whether the single-level logistic regression analysis was taken with Bayesian approach or not. This needs to be clarified.

7. Lines: 190-194: The authors did not consider interaction terms in the multinomial multilevel regression analysis. I wonder if this is due to the computational difficulty or convergence problems for the considered models? Also, does it need

proportional odds assumption here? The authors may discuss these in the manuscript.
