# Supplementary material for: Multilevel analysis of predictors of multiple indicators of childhood vaccination in Nigeria
Source: PLoS One. 2022 May 25;17(5):e0269066. doi: 10.1371/journal.pone.0269066 (PMC9132327; doi:10.1371/journal.pone.0269066)
Supplement: Attachment — Submitted filename: Response to Reviewers _R1.docx [file pone.0269066.s003.docx]

Dear Editor,

Thank you for the opportunity to once again revise our manuscript titled “Multilevel analysis of predictors of multiple indicators of childhood vaccination in Nigeria”. We are extremely grateful to the Editors and the Reviewers for the constructive criticism of the manuscript and the positive feedback which certainly helped to improve the message and the quality of our manuscript. We are indeed grateful.

We have duly addressed all the concerns raised by the Editors and the Reviewers to the best of our ability and wish to re-submit the revised version for your consideration and subsequent publication in your cherished journal, and together we can all help in addressing this serious public health challenge facing millions of children globally, especially in developing countries like Nigeria.

**Response to Editorial comments**

When submitting your revision, we need you to address these additional requirements.

1. Please ensure that your manuscript meets PLOS ONE's style requirements, including those for file naming. The PLOS ONE style templates can be found at 
[https://journals.plos.org/plosone/s/file?id=wjVg/PLOSOne_formatting_sample_main_body.pdf](https://eur03.safelinks.protection.outlook.com/?url=https%3A%2F%2Fjournals.plos.org%2Fplosone%2Fs%2Ffile%3Fid%3DwjVg%2FPLOSOne_formatting_sample_main_body.pdf&data=04%7C01%7CJ.M.K.Aheto%40soton.ac.uk%7C2eeb756a838d454a7f6b08d9ae564e02%7C4a5378f929f44d3ebe89669d03ada9d8%7C0%7C0%7C637732507988953352%7CUnknown%7CTWFpbGZsb3d8eyJWIjoiMC4wLjAwMDAiLCJQIjoiV2luMzIiLCJBTiI6Ik1haWwiLCJXVCI6Mn0%3D%7C3000&sdata=zZAXBIwTpAByED3fIXltUVy3aCtLO5bnjZugFPZInkk%3D&reserved=0) and 
[https://journals.plos.org/plosone/s/file?id=ba62/PLOSOne_formatting_sample_title_authors_affiliations.pdf](https://eur03.safelinks.protection.outlook.com/?url=https%3A%2F%2Fjournals.plos.org%2Fplosone%2Fs%2Ffile%3Fid%3Dba62%2FPLOSOne_formatting_sample_title_authors_affiliations.pdf&data=04%7C01%7CJ.M.K.Aheto%40soton.ac.uk%7C2eeb756a838d454a7f6b08d9ae564e02%7C4a5378f929f44d3ebe89669d03ada9d8%7C0%7C0%7C637732507988963329%7CUnknown%7CTWFpbGZsb3d8eyJWIjoiMC4wLjAwMDAiLCJQIjoiV2luMzIiLCJBTiI6Ik1haWwiLCJXVCI6Mn0%3D%7C3000&sdata=7kp1fcPSeZRPWuUSaNgjFw6Q5vJmHFRCYOVro3yj1Ic%3D&reserved=0)

**Response:** We have revised the manuscript to address the issues about the style requirements of the journal throughout the manuscript text.

2. Thank you for stating the following in the Acknowledgments/ Funding Section of your manuscript:

This work was supported by funding from the Bill and Melinda Gates Foundation (Investment ID INV-003287). CEU received the grant. The funder did not play any role in the study design, data collection, analysis and interpretation of data, the report writing, and the decision to submit the manuscript for publication.

Please note that funding information should not appear in the Acknowledgments section or other areas of your manuscript. We will only publish funding information present in the Funding Statement section of the online submission form. 
Please remove any funding-related text from the manuscript and let us know how you would like to update your Funding Statement. Currently, your Funding Statement reads as follows:

This work was supported by funding from the Bill and Melinda Gates Foundation (Investment ID INV-003287). CEU received the grant. The funder did not play any role in the study design, data collection, analysis and interpretation of data, the report writing, and the decision to submit the manuscript for publication.

Please include your amended statements within your cover letter; we will change the online submission form on your behalf.

**Response:** We removed the funding information under the Funding section of the manuscript and stated this as “This work was supported by funding from the Bill and Melinda Gates Foundation (Investment ID INV-003287). CEU and AJT received the grant. The funder did not play any role in the study design, data collection, analysis and interpretation of data, the report writing, and the decision to submit the manuscript for publication” in the cover letter as directed. The Editors should use this for the publication.

3. Please note that in order to use the direct billing option the corresponding author must be affiliated with the chosen institute. Please either amend your manuscript to change the affiliation or corresponding author, or email us at [plosone@plos.org](mailto:plosone@plos.org) with a request to remove this option.

**Response:** Thank you for this information. We revised the corresponding author’s affiliation to reflect this. The corresponding author’s address and affiliations are as follows in order as they appear:

1) WorldPop, School of Geography and Environmental Science, University of Southampton, Southampton, SO17 1BJ, UK.

2) Department of Biostatistics, School of Public Health, College of Health Sciences, University of Ghana, Accra - Ghana”.

We have revised the affiliations of the other authors accordingly to reflect the changes made. (see the title page).

4. We note that you have indicated that data from this study are available upon request. PLOS only allows data to be available upon request if there are legal or ethical restrictions on sharing data publicly. For more information on unacceptable data access restrictions, please see [http://journals.plos.org/plosone/s/data-availability#loc-unacceptable-data-access-restrictions](https://eur03.safelinks.protection.outlook.com/?url=http%3A%2F%2Fjournals.plos.org%2Fplosone%2Fs%2Fdata-availability%23loc-unacceptable-data-access-restrictions&data=04%7C01%7CJ.M.K.Aheto%40soton.ac.uk%7C2eeb756a838d454a7f6b08d9ae564e02%7C4a5378f929f44d3ebe89669d03ada9d8%7C0%7C0%7C637732507988963329%7CUnknown%7CTWFpbGZsb3d8eyJWIjoiMC4wLjAwMDAiLCJQIjoiV2luMzIiLCJBTiI6Ik1haWwiLCJXVCI6Mn0%3D%7C3000&sdata=BRzN98f5eSD60tDIU%2B4vnNppahAyK63P8JFfD87i3gE%3D&reserved=0).

In your revised cover letter, please address the following prompts:

a) If there are ethical or legal restrictions on sharing a de-identified data set, please explain them in detail (e.g., data contain potentially sensitive information, data are owned by a third-party organization, etc.) and who has imposed them (e.g., an ethics committee). Please also provide contact information for a data access committee, ethics committee, or other institutional body to which data requests may be sent.

**Response:** All the data sets supporting the analyses in this manuscript are publicly available and can be obtained by sending data request to the owners or by accessing them from the owner’s website. We provided full details on the data sources, and references to enable readers to access these data. We are unable to directly share this data with third parties because we did not have permission from the data owners to do so. We have provided additional text as “The authors did not have any special access privileges that others would not have” to the data availability section.

b) If there are no restrictions, please upload the minimal anonymized data set necessary to replicate your study findings as either Supporting Information files or to a stable, public repository and provide us with the relevant URLs, DOIs, or accession numbers. For a list of acceptable repositories, please see [http://journals.plos.org/plosone/s/data-availability#loc-recommended-repositories](https://eur03.safelinks.protection.outlook.com/?url=http%3A%2F%2Fjournals.plos.org%2Fplosone%2Fs%2Fdata-availability%23loc-recommended-repositories&data=04%7C01%7CJ.M.K.Aheto%40soton.ac.uk%7C2eeb756a838d454a7f6b08d9ae564e02%7C4a5378f929f44d3ebe89669d03ada9d8%7C0%7C0%7C637732507988963329%7CUnknown%7CTWFpbGZsb3d8eyJWIjoiMC4wLjAwMDAiLCJQIjoiV2luMzIiLCJBTiI6Ik1haWwiLCJXVCI6Mn0%3D%7C3000&sdata=QtQX8J9NuyOFeijnfQ8rEiNA%2FofrlnhC2OZN3t6M8Z0%3D&reserved=0).

We will update your Data Availability statement on your behalf to reflect the information you provide.

**Response:** All the data (both DHS and Geospatial variables) supporting the analyses presented in this manuscript are publicly available and sources provided in the methods section. In addition, we presented the description, the sources, and the references to the geospatial covariates used in our work in Table S2, and also under “Geospatial covariate description and processing steps” in the Supplementary Information.

5. We note that Figure S1 in your submission contain [map/satellite] images which may be copyrighted. All PLOS content is published under the Creative Commons Attribution License (CC BY 4.0), which means that the manuscript, images, and Supporting Information files will be freely available online, and any third party is permitted to access, download, copy, distribute, and use these materials in any way, even commercially, with proper attribution. For these reasons, we cannot publish previously copyrighted maps or satellite images created using proprietary data, such as Google software (Google Maps, Street View, and Earth). For more information, see our copyright guidelines: [http://journals.plos.org/plosone/s/licenses-and-copyright](https://eur03.safelinks.protection.outlook.com/?url=http%3A%2F%2Fjournals.plos.org%2Fplosone%2Fs%2Flicenses-and-copyright&data=04%7C01%7CJ.M.K.Aheto%40soton.ac.uk%7C2eeb756a838d454a7f6b08d9ae564e02%7C4a5378f929f44d3ebe89669d03ada9d8%7C0%7C0%7C637732507988973288%7CUnknown%7CTWFpbGZsb3d8eyJWIjoiMC4wLjAwMDAiLCJQIjoiV2luMzIiLCJBTiI6Ik1haWwiLCJXVCI6Mn0%3D%7C3000&sdata=yK%2BvanPIVKek5Lc7y4wN4OEh7FUdWomqlseECzyFt0w%3D&reserved=0).
 
We require you to either (1) present written permission from the copyright holder to publish these figures specifically under the CC BY 4.0 license, or (2) remove the figures from your submission:
 
a. You may seek permission from the original copyright holder of Figure S1 to publish the content specifically under the CC BY 4.0 license.  
We recommend that you contact the original copyright holder with the Content Permission Form ([http://journals.plos.org/plosone/s/file?id=7c09/content-permission-form.pdf](https://eur03.safelinks.protection.outlook.com/?url=http%3A%2F%2Fjournals.plos.org%2Fplosone%2Fs%2Ffile%3Fid%3D7c09%2Fcontent-permission-form.pdf&data=04%7C01%7CJ.M.K.Aheto%40soton.ac.uk%7C2eeb756a838d454a7f6b08d9ae564e02%7C4a5378f929f44d3ebe89669d03ada9d8%7C0%7C0%7C637732507988973288%7CUnknown%7CTWFpbGZsb3d8eyJWIjoiMC4wLjAwMDAiLCJQIjoiV2luMzIiLCJBTiI6Ik1haWwiLCJXVCI6Mn0%3D%7C3000&sdata=egcV6tRh9Q5eeukb1nXS%2B2ciAa%2F9kjnkhKPOC1KvGXE%3D&reserved=0)) and the following text:
“I request permission for the open-access journal PLOS ONE to publish XXX under the Creative Commons Attribution License (CCAL) CC BY 4.0 ([http://creativecommons.org/licenses/by/4.0/](https://eur03.safelinks.protection.outlook.com/?url=http%3A%2F%2Fcreativecommons.org%2Flicenses%2Fby%2F4.0%2F&data=04%7C01%7CJ.M.K.Aheto%40soton.ac.uk%7C2eeb756a838d454a7f6b08d9ae564e02%7C4a5378f929f44d3ebe89669d03ada9d8%7C0%7C0%7C637732507988983211%7CUnknown%7CTWFpbGZsb3d8eyJWIjoiMC4wLjAwMDAiLCJQIjoiV2luMzIiLCJBTiI6Ik1haWwiLCJXVCI6Mn0%3D%7C3000&sdata=KN5yEWm91FHPmRGn9mGKd1%2FClCHk1ALGWqZBFjcxZcs%3D&reserved=0)). Please be aware that this license allows unrestricted use and distribution, even commercially, by third parties. Please reply and provide explicit written permission to publish XXX under a CC BY license and complete the attached form.”
 
Please upload the completed Content Permission Form or other proof of granted permissions as an "Other" file with your submission.
 
In the figure caption of the copyrighted figure, please include the following text: “Reprinted from [ref] under a CC BY license, with permission from [name of publisher], original copyright [original copyright year].”
**Response:** The Figure S1 was produced by the authors for this manuscript. Thus, we did not need any permission to use it, and so did not make any changes to the manuscript.

b. If you are unable to obtain permission from the original copyright holder to publish these figures under the CC BY 4.0 license or if the copyright holder’s requirements are incompatible with the CC BY 4.0 license, please either i) remove the figure or ii) supply a replacement figure that complies with the CC BY 4.0 license. Please check copyright information on all replacement figures and update the figure caption with source information. If applicable, please specify in the figure caption text when a figure is similar but not identical to the original image and is therefore for illustrative purposes only.
The following resources for replacing copyrighted map figures may be helpful:
 
USGS National Map Viewer (public domain): [http://viewer.nationalmap.gov/viewer/](https://eur03.safelinks.protection.outlook.com/?url=http%3A%2F%2Fviewer.nationalmap.gov%2Fviewer%2F&data=04%7C01%7CJ.M.K.Aheto%40soton.ac.uk%7C2eeb756a838d454a7f6b08d9ae564e02%7C4a5378f929f44d3ebe89669d03ada9d8%7C0%7C0%7C637732507988983211%7CUnknown%7CTWFpbGZsb3d8eyJWIjoiMC4wLjAwMDAiLCJQIjoiV2luMzIiLCJBTiI6Ik1haWwiLCJXVCI6Mn0%3D%7C3000&sdata=Zy5xSTXzZRZDYB31t%2B8H4hRa6UZ3mlgKeRYUI0LfmcA%3D&reserved=0)
The Gateway to Astronaut Photography of Earth (public domain): [http://eol.jsc.nasa.gov/sseop/clickmap/](https://eur03.safelinks.protection.outlook.com/?url=http%3A%2F%2Feol.jsc.nasa.gov%2Fsseop%2Fclickmap%2F&data=04%7C01%7CJ.M.K.Aheto%40soton.ac.uk%7C2eeb756a838d454a7f6b08d9ae564e02%7C4a5378f929f44d3ebe89669d03ada9d8%7C0%7C0%7C637732507988983211%7CUnknown%7CTWFpbGZsb3d8eyJWIjoiMC4wLjAwMDAiLCJQIjoiV2luMzIiLCJBTiI6Ik1haWwiLCJXVCI6Mn0%3D%7C3000&sdata=gpsgHlvIHd2%2F3sv9aY2kKpttaUXKUFQDni6pDlYfvc4%3D&reserved=0)
Maps at the CIA (public domain): [https://www.cia.gov/library/publications/the-world-factbook/index.html](https://eur03.safelinks.protection.outlook.com/?url=https%3A%2F%2Fwww.cia.gov%2Flibrary%2Fpublications%2Fthe-world-factbook%2Findex.html&data=04%7C01%7CJ.M.K.Aheto%40soton.ac.uk%7C2eeb756a838d454a7f6b08d9ae564e02%7C4a5378f929f44d3ebe89669d03ada9d8%7C0%7C0%7C637732507988993179%7CUnknown%7CTWFpbGZsb3d8eyJWIjoiMC4wLjAwMDAiLCJQIjoiV2luMzIiLCJBTiI6Ik1haWwiLCJXVCI6Mn0%3D%7C3000&sdata=ubq3ZikTiLRFaIyUaZsQ558v5uZG5qcHoxlLblNR1Pw%3D&reserved=0) and [https://www.cia.gov/library/publications/cia-maps-publications/index.html](https://eur03.safelinks.protection.outlook.com/?url=https%3A%2F%2Fwww.cia.gov%2Flibrary%2Fpublications%2Fcia-maps-publications%2Findex.html&data=04%7C01%7CJ.M.K.Aheto%40soton.ac.uk%7C2eeb756a838d454a7f6b08d9ae564e02%7C4a5378f929f44d3ebe89669d03ada9d8%7C0%7C0%7C637732507988993179%7CUnknown%7CTWFpbGZsb3d8eyJWIjoiMC4wLjAwMDAiLCJQIjoiV2luMzIiLCJBTiI6Ik1haWwiLCJXVCI6Mn0%3D%7C3000&sdata=qH%2B49Bd5%2FmoXgc0EX8P3w%2BlrnZU6EivPlc0RRdyBzmk%3D&reserved=0)
NASA Earth Observatory (public domain): [http://earthobservatory.nasa.gov/](https://eur03.safelinks.protection.outlook.com/?url=http%3A%2F%2Fearthobservatory.nasa.gov%2F&data=04%7C01%7CJ.M.K.Aheto%40soton.ac.uk%7C2eeb756a838d454a7f6b08d9ae564e02%7C4a5378f929f44d3ebe89669d03ada9d8%7C0%7C0%7C637732507989003155%7CUnknown%7CTWFpbGZsb3d8eyJWIjoiMC4wLjAwMDAiLCJQIjoiV2luMzIiLCJBTiI6Ik1haWwiLCJXVCI6Mn0%3D%7C3000&sdata=2%2FMWXMFmF%2FbTW2g8V%2BKHc4UfcKT%2FdR9v7JU7Up0K%2FdA%3D&reserved=0)
Landsat: [http://landsat.visibleearth.nasa.gov/](https://eur03.safelinks.protection.outlook.com/?url=http%3A%2F%2Flandsat.visibleearth.nasa.gov%2F&data=04%7C01%7CJ.M.K.Aheto%40soton.ac.uk%7C2eeb756a838d454a7f6b08d9ae564e02%7C4a5378f929f44d3ebe89669d03ada9d8%7C0%7C0%7C637732507989003155%7CUnknown%7CTWFpbGZsb3d8eyJWIjoiMC4wLjAwMDAiLCJQIjoiV2luMzIiLCJBTiI6Ik1haWwiLCJXVCI6Mn0%3D%7C3000&sdata=SmhhZCODjqlJmTV%2FAQem871TEF7xOe68Cfk1mEWpYig%3D&reserved=0)
USGS EROS (Earth Resources Observatory and Science (EROS) Center) (public domain): [http://eros.usgs.gov/#](https://eur03.safelinks.protection.outlook.com/?url=http%3A%2F%2Feros.usgs.gov%2F%23&data=04%7C01%7CJ.M.K.Aheto%40soton.ac.uk%7C2eeb756a838d454a7f6b08d9ae564e02%7C4a5378f929f44d3ebe89669d03ada9d8%7C0%7C0%7C637732507989013092%7CUnknown%7CTWFpbGZsb3d8eyJWIjoiMC4wLjAwMDAiLCJQIjoiV2luMzIiLCJBTiI6Ik1haWwiLCJXVCI6Mn0%3D%7C3000&sdata=NKMNv1YuW98JMFCooT7ARv%2Fr7YgKMJtnSc3qlJpCA6g%3D&reserved=0)
Natural Earth (public domain): [http://www.naturalearthdata.com/](https://eur03.safelinks.protection.outlook.com/?url=http%3A%2F%2Fwww.naturalearthdata.com%2F&data=04%7C01%7CJ.M.K.Aheto%40soton.ac.uk%7C2eeb756a838d454a7f6b08d9ae564e02%7C4a5378f929f44d3ebe89669d03ada9d8%7C0%7C0%7C637732507989013092%7CUnknown%7CTWFpbGZsb3d8eyJWIjoiMC4wLjAwMDAiLCJQIjoiV2luMzIiLCJBTiI6Ik1haWwiLCJXVCI6Mn0%3D%7C3000&sdata=XhkQ%2BszuoTqPf0Pwkd3myzdQZmV0RyjrETrf2%2FCvHy4%3D&reserved=0)

**Response:** The maps and its contents presented in Figure S1 in the Supplementary Information was produced by the authors using publicly available data in ArcGIS software for this paper, and the data source were cited in Table S2 in the Supporting Information. We did not need any permission to use this figure as it was produced by us. We have published similar maps in previous studies[1-3]. Hence, we did not make any changes to the manuscript.

**Reference**

1.	Utazi CE, Thorley J, Alegana VA, Ferrari MJ, Takahashi S, Metcalf CJE, et al. Mapping vaccination coverage to explore the effects of delivery mechanisms and inform vaccination strategies. Nature Communications. 2019;10(1):1633. doi: 10.1038/s41467-019-09611-1.

2.	Utazi CE, Wagai J, Pannell O, Cutts FT, Rhoda DA, Ferrari MJ, et al. Geospatial variation in measles vaccine coverage through routine and campaign strategies in Nigeria: Analysis of recent household surveys. Vaccine. 2020;38(14):3062-71. doi: <https://doi.org/10.1016/j.vaccine.2020.02.070>.

3.	Utazi CE, Thorley J, Alegana VA, Ferrari MJ, Takahashi S, Metcalf CJE, et al. High resolution age-structured mapping of childhood vaccination coverage in low and middle income countries. Vaccine. 2018;36(12):1583-91. Epub 2018/02/14. doi: 10.1016/j.vaccine.2018.02.020. PubMed PMID: 29454519; PubMed Central PMCID: PMCPMC6344781.

6. Please include captions for your Supporting Information files at the end of your manuscript, and update any in-text citations to match accordingly. Please see our Supporting Information guidelines for more information: [http://journals.plos.org/plosone/s/supporting-information](https://eur03.safelinks.protection.outlook.com/?url=http%3A%2F%2Fjournals.plos.org%2Fplosone%2Fs%2Fsupporting-information&data=04%7C01%7CJ.M.K.Aheto%40soton.ac.uk%7C2eeb756a838d454a7f6b08d9ae564e02%7C4a5378f929f44d3ebe89669d03ada9d8%7C0%7C0%7C637732507989013092%7CUnknown%7CTWFpbGZsb3d8eyJWIjoiMC4wLjAwMDAiLCJQIjoiV2luMzIiLCJBTiI6Ik1haWwiLCJXVCI6Mn0%3D%7C3000&sdata=IB61wSdwv0mnmX1XW7US%2BqsPYdDCASQN2dagc%2BZYTfE%3D&reserved=0).

**Response:** We revised the manuscript to reflect these changes. The marked changes can be found after the reference list under the heading Supporting Information captions.

[Note: HTML markup is below. Please do not edit.]

Reviewers' comments:

Reviewer's Responses to Questions

**Comments to the Author**

1. Is the manuscript technically sound, and do the data support the conclusions?

The manuscript must describe a technically sound piece of scientific research with data that supports the conclusions. Experiments must have been conducted rigorously, with appropriate controls, replication, and sample sizes. The conclusions must be drawn appropriately based on the data presented.

Reviewer #1: Yes

Reviewer #2: Yes

2. Has the statistical analysis been performed appropriately and rigorously?

Reviewer #1: Yes

Reviewer #2: Yes

3. Have the authors made all data underlying the findings in their manuscript fully available?

The [PLOS Data policy](https://eur03.safelinks.protection.outlook.com/?url=http%3A%2F%2Fwww.plosone.org%2Fstatic%2Fpolicies.action%23sharing&data=04%7C01%7CJ.M.K.Aheto%40soton.ac.uk%7C2eeb756a838d454a7f6b08d9ae564e02%7C4a5378f929f44d3ebe89669d03ada9d8%7C0%7C0%7C637732507989023068%7CUnknown%7CTWFpbGZsb3d8eyJWIjoiMC4wLjAwMDAiLCJQIjoiV2luMzIiLCJBTiI6Ik1haWwiLCJXVCI6Mn0%3D%7C3000&sdata=2PL4vsxuR3dphRzG6s6ES4VdHVPLkeA5lWEMFS%2BlO9M%3D&reserved=0) requires authors to make all data underlying the findings described in their manuscript fully available without restriction, with rare exception (please refer to the Data Availability Statement in the manuscript PDF file). The data should be provided as part of the manuscript or its supporting information, or deposited to a public repository. For example, in addition to summary statistics, the data points behind means, medians and variance measures should be available. If there are restrictions on publicly sharing data—e.g. participant privacy or use of data from a third party—those must be specified.

Reviewer #1: Yes

Reviewer #2: Yes

4. Is the manuscript presented in an intelligible fashion and written in standard English?

PLOS ONE does not copyedit accepted manuscripts, so the language in submitted articles must be clear, correct, and unambiguous. Any typographical or grammatical errors should be corrected at revision, so please note any specific errors here.

Reviewer #1: Yes

Reviewer #2: Yes

5. Review Comments to the Author

Please use the space provided to explain your answers to the questions above. You may also include additional comments for the author, including concerns about dual publication, research ethics, or publication ethics. (Please upload your review as an attachment if it exceeds 20,000 characters)

**Reviewer #1: Overall comment:**

This study investigates multiple indicators of childhood vaccination in Nigeria. Using data from the 2018 Nigeria Demographic and Health Survey and geospatial data sets, authors built Bayesian multilevel binomial and multinomial logistic regression models to analyze independent predictors of three vaccination outcomes. Although authors claimed several innovations, including they examined separately factors associated with beginning (PENTA1) and completing (PENTA3/1 and MV) the vaccination series; included community-level geospatial factors as well as individual attributes, and used multinomial analyses to examine potential differences in findings when analysis, I think this study has normal quality. A major revision can be reached. The model design and the description of results are good, but there are still some issues that should be improved before publish.
**Response:** Thank you for the positive and helpful comments.

1. Line 66-68. The major finding is not so attractive for me. Because it is basically a piece of general knowledge, we can easily to imagine the result.
**Response:** Thank you. To our knowledge, our study is the first of its kind to examine the relationships between vaccination and a range of predictor variables based on strength of evidence of vaccination, i.e., documented versus recall evidence of vaccination. We consider our finding that “factors associated with vaccination were broadly similar for documented versus recall evidence of vaccination” to be highly significant as this will help guide policymakers when making decisions using different evidence of vaccination available to them. Moreover, our findings are important because we were also interested in establishing which predictor might be associated with all the three outcomes (Penta1, Penta3/1, and MV) under investigation. Hence, policies addressing the common predictors of all three outcomes is likely to generate greater improvements in coverage levels.

2. Line 92-114: Although the motivation and objective are clear, the quality of the introduction is improvable. There must be several relative studies that use a similar approach to investigate the problem, but the authors didn’t mention about it.
**Response:** Thank you. We revised the manuscript to reflect this. See line 122, and lines 125-138 at page 4.

3. Line 104-106: More than ten cited references are included in only one sentence. Authors are suggested to address more detail about them.
**Response:** Thank you. We revised the manuscript to expatiate on this. See lines 115-121 at pages 3 and 4.

4. Line 155: I think authors include the geospatial covariates in models is a very good approach. But I suggest authors can present the geospatial covariates by maps.
**Response:** Thank you for the positive feedback. We have already provided the maps for the geospatial covariates used in our models in Figure S1 in the Supplementary Information at page 6.

5. Line 175-211: The model design is praiseworthy.
**Response:** Thank you. We appreciate this feedback.

6. Line 308-313: Similarly, I suggest authors can display the results of the effect of geospatial factors by maps.
**Response:** Thank you. The effects of the geospatial covariates are estimated for the entire country in our models. It will make sense to display maps of these if they were estimated for each region, for example. We will explore this detailed analysis in future work as we have now highlighted in the discussion (see lines 440-443 at page 17). However, we mapped the values of all the geospatial covariates retained in our models across the entire Nigeria to help the readers appreciate and understand the spatial variation in these covariates. See Figure S1 in the Supplementary Information at page 6.

7. Figures: The quality and resolution of figures are very bad. It’s hard to know the results.
**Response:** Thank you. The figures as appeared on our system as .tiff file are all clear when you open them. Perhaps, what you observed might be due to the online system combining all the files as PDF. Note that we used the ‘Preflight Analysis and Conversion Engine (PACE) digital diagnostic tool recommended by PLOS ONE and available at [https://pacev2.apexcovantage.com/](https://eur03.safelinks.protection.outlook.com/?url=https%3A%2F%2Fpacev2.apexcovantage.com%2F&data=04%7C01%7CJ.M.K.Aheto%40soton.ac.uk%7C2eeb756a838d454a7f6b08d9ae564e02%7C4a5378f929f44d3ebe89669d03ada9d8%7C0%7C0%7C637732507989033024%7CUnknown%7CTWFpbGZsb3d8eyJWIjoiMC4wLjAwMDAiLCJQIjoiV2luMzIiLCJBTiI6Ik1haWwiLCJXVCI6Mn0%3D%7C3000&sdata=1exDiRIcC4EmgOTeh0e2EKTbWXuFxpQMfyO%2FOC18gMU%3D&reserved=0) to prepare our figures to the acceptable format and the standard required by PLOS ONE. Also, we saved these figures in R software directly as .tiff files and then used the PACE tool to prepare them as required by the journal, and all the figures were approved as meeting the PLOS ONE standard by the PACE system.

8. Tables: I think some numerical statistical findings can be presented as tables.

**Response:** Thank you. Given the numerous numbers of parameters involved in our models, it is preferable to summarise the results in figures instead of tables for the main manuscript text to sustain readers interests while the tables for the same results are sent to the Supplementary Information. It is also easier to understand the results as presented in the figures than in tables while maintaining the scientific meaning of these results. However, note that we have already provided same results in the supplementary tables in the Supplementary Information for those who might also be interested in reading these results in tables (see Tables S3-S13). Thus, we did not make any changes to the manuscript.

**Reviewer #2: Review of PLUS ONE**
PONE-D-21-28231: " Multilevel analysis of predictors of multiple indicators of childhood vaccination in Nigeria "

The manuscript presents a reasonably well-articulated discussion for the developed framework to address childhood vaccination inequalities in Nigeria. Based on my reading, the applications of multilevel logistic models to produce new insights to data analysis makes the current manuscript contributing to the literature of public health. The work will be of also interest to a wide readership in the journal. Although the manuscript is worth published, its present form needs some revisions and I have the following suggestions.
**Response:** We appreciate your positive feedback.

1. In the Introduction, the authors omitted the brief description about multilevel models. Some details can be found at supplementary file but I think the associated discussions could be summarized into the Introduction. In so doing, the article would be more complete and clear.
**Response:** Thank you. We appreciate your feedback. We wish to state that the Introduction section is not where models are described and that the details of the models used in the work are already given in the Data analysis section from pages 7-9 and in the Supplementary Information section from page 7-12. Thus, we did not make any changes to the manuscript.

2. In the Data section, the authors may provide a figure for the hieratical structure of the data, which can furnish the rationale why to use multilevel models.

**Response:** Thank you for this useful feedback. We now produced the suggested figure, but we prefer to put this figure in the Supplementary Information (see Fig S2 at page 7). We revised the manuscript to reflect this. See line 212-213 at page 8.

On the other hand, for readability and the ease of understanding, the authors should at least include some model equations for the multilevel models in the section of Data analysis, rather than just assemble all the materials in the supplementary.
**Response:** Thank you. The authors were very careful about the need for the general audience to understand the paper. Thus, this paper hinges on both public health and statistical methodology. Our approach of sending our multilevel model equations to the Supplementary Information while describing them in the main manuscript text is strategic – (1) not to make the paper an overkill of statistical methodology which will make it very difficult for the general public health experts to understand and (2) to sustain readers interests. Fortunately, the statistical experts or modellers who are interested in these multilevel model equations can easily access them in detail in the Supplementary Information. In addition, we provided sufficient information about the multilevel models used in this paper in the main manuscript text to allow readers to follow the paper easily. As a result, we are of firm believe that is best to leave the multilevel model equations in the Supplementary Information. Thus, we did not make any changes to the manuscript.

3. Is there any specific reason to use Bayesian multilevel models? As the ‘frequentist’ multilevel logistic regression models exist as alternative ways to analyze the data, I suggest the authors explain a bit what to motivate the Bayesian multilevel analyses and why to use them both in the Introduction and Data analysis sections.
**Response:** Thank you. Firstly, one of our interests is to employ Bayesian modelling approach to investigate the problem. Undoubtedly, the Bayesian modelling is one of the novel approaches in model fitting and model diagnostics. For example, in the Bayesian approach, two different sources of uncertainties (i.e., uncertainty in the parameter values and sampling uncertainty) in our estimates can be quantified and the 95% credible intervals fixed while the estimated parameters are allowed to vary unlike the frequentist (i.e., classical) approach. Also, the type of the modelling implemented in this study, especially the multiple multilevel multinomial and binomial regression models can effectively and efficiently be implemented in the Bayesian framework compared to the frequentist due to the model complexity and computational cost. These are the key reasons we opted for the Bayesian approach. However, we explore the data using cross-tabulations, and single-level logistic regression model based on frequentist approach to determine how each of the covariates relates with the outcomes. We revised the manuscript to reflect this in the Supplementary Information under the heading ‘Bayesian models’ in lines 208-219.

Also, as presented already in the original manuscript in the ‘Supplementary Information’ under the heading ‘Prior distributions for fixed and random effects’, note that the results from the frequentist models were used to provide initial values for the Bayesian multilevel models (i.e., both the binomial and the multinomial). Thus, the results from the frequentist models were used as an input for the final Bayesian models as already captured in the Supplementary Information (see lines 193-203).

4. Regarding the model specifications, do the multilevel models only include random intercepts? Is there any random coefficients for the predictors? From the model equation 2 and equation 3 in the supplementary file, it is not clear to me how the multilevel representation is given.

**Response:** Thank you. We used only random intercept multilevel models as already stated in the Supplementary Information, and we did not include random coefficient for the predictors because our goal is to examine whether the outcomes vary by the levels of hierarchy. Thus, our models did not include random slopes. This can be seen in the texts and our multilevel model equations presented in the Supplementary Information (see lines 109-111 and Equation (1) at page 8, line 120 Equation (2) at page 9, and lines 172-173, 180-181 at page 11).

5. The authors indicated in the supplementary that non-informative priors can be alternative choice of priors for the model parameters. I was actually thinking that would it be possible to generate better analysis results as there is lack of information on reliable priors. Although I don‘t demand the authors to do so, the discussion needs to be added.
**Response:** Thank you. We have already discussed this in the ‘Supplementary Information’ under the heading ‘Prior distributions for fixed and random effects’ (see lines 193-203). We stated that due to lack of information on reliable priors, we fitted multilevel models via the frequentist approach and used these results as the initial values (priors) for the Bayesian models. Even though this is a reasonable approach and better than the non-informative priors approach, we were informing the readers that there is another approach where one can also use the non-informative priors, and we stated this in the same section already (see lines 193-203). Definitely, our approach of fitting the frequentist model to the data and using the parameters from these models as the priors for the Bayesian models is better than using the non-informative priors. Our approach was successfully used in a previous study [1]. We now provided the reference to support our approach in the Supplementary Information (see line 202 at page 12).

**Reference**

1. Aheto JMK, Taylor BM, Keegan TJ, Diggle PJ. Modelling and forecasting spatio-temporal variation in the risk of chronic malnutrition among under-five children in Ghana. Spat Spatiotemporal Epidemiol. 2017;21:37-46. Epub 2017/03/02. doi: 10.1016/j.sste.2017.02.003. PubMed PMID: 28552186.

6. Lines 168-174: It is not clear whether the single-level logistic regression analysis was taken with Bayesian approach or not. This needs to be clarified.
**Response:** Thank you for the feedback. For the single level models presented, we used the frequentist approach as already stated in the original manuscript in lines 170-171 (i.e., “…fitted frequentist single level simple logistic regression models to obtain the corresponding …” but now in lines 202-205at page 7 of the revised main manuscript. Also, we already stated this in the supplementary tables. We already reported this under ‘Cross-tabulations and single-level logistic regression analysis’ in the main manuscript under ‘Data analysis’ (see lines 202-205 at page 7), and in supplementary Tables S4, S5, S7, and S9 in the Supplementary Information.

7. Lines: 190-194: The authors did not consider interaction terms in the multinomial multilevel regression analysis. I wonder if this is due to the computational difficulty or convergence problems for the considered models? Also, does it need proportional odds assumption here? The authors may discuss these in the manuscript.

**Response:** Thank you. We did not consider the interaction terms for the multiple multilevel multinomial models due to model complexities and non-convergence challenges. We revised the manuscript to reflect this (see line 229 at page 8). Also, our multinomial outcomes PENTA1 and MV are nominal outcomes and not ordinal outcomes and so does not require the proportional odds assumptions.
