# Supplementary material for: Multilevel analysis of predictors of multiple indicators of childhood vaccination in Nigeria
Source: PLoS One. 2022 May 25;17(5):e0269066. doi: 10.1371/journal.pone.0269066 (PMC9132327; doi:10.1371/journal.pone.0269066)
Supplement: Attachment — Submitted filename: Response to Reviewers.docx [file pone.0269066.s004.docx]

Dear Editor,

Thank you for the opportunity to once again revise our manuscript titled “Multilevel analysis of predictors of multiple indicators of childhood vaccination in Nigeria”. We are extremely grateful to the Editors and the Reviewers for the constructive criticism of the manuscript and the positive feedback which certainly helped to improve the quality of our manuscript.

We have duly addressed all the concerns raised by the Editors and the Reviewers to the best of our ability and wish to submit the revised version for your consideration and subsequent publication in your cherished journal, and together we can all help in addressing this serious public health challenge facing millions of children globally, especially in developing countries like Nigeria.

**Response to Editorial comments**

**Journal Requirements:**

Please review your reference list to ensure that it is complete and correct. If you have cited papers that have been retracted, please include the rationale for doing so in the manuscript text, or remove these references and replace them with relevant current references. Any changes to the reference list should be mentioned in the rebuttal letter that accompanies your revised manuscript. If you need to cite a retracted article, indicate the article’s retracted status in the References list and also include a citation and full reference for the retraction notice.

**Response:** We reviewed our reference list and can confirm that they are correct and complete. Also, we did not find any retracted paper among our references. Thus, we did not make any changes to the manuscript and the reference list.

**Additional Editor Comments:**

The reviews think all comments have been addressed. However, the reviewer concerns the quality and resolution of the figures. We are happy to publish the paper if the quality of the figure can be further improved.

**Response:** Thank you for handling our manuscript smoothly and your decision for us to further improve our figures before our manuscript is accepted for publication. We suspect that the reviewer might not have opened the figure from the link provided at the top of each figure in the reviewer’s PDF document and appeared to have relied on what he/she saw directly on the reviewers PDF document to arrive at this concern. Please, we prepared our figures using the correct figure formats, including correct resolutions based on PLOS ONE recommended free tool (<https://pacev2.apexcovantage.com/>) and the quality and resolution was great on our systems. Please, may I ask the Editor to download the original figures from the link at the top of each figure in the reviewer’s PDF document for your own assessment. Always, the system that generates the reviewers document for review is responsible for the relatively low figure quality observed by the reviewer so reviewers must always download the actual figures from the link at the top of each figure which always have the quality and the required resolution from the PACE tool as recommended by PLOS ONE.

However, we prepared new figures again using much higher resolution and used the PACE software to format the figures as recommended by PLOS ONE to improve the quality and resolution as requested.

Per my own experience with publishing figures in PLOS ONE, and reviewing manuscripts containing figures for PLOS ONE, the quality and resolution of such figures is mostly very poor in the reviewers document even if one submitted a very high quality and resolution images/figures just as in our case now. As a result, reviewers who are not aware of this will surely raise concerns about the figures as in our case, but same figures look great when reviewers download them from the link in the reviewer’s PDF document at the top of each figure and they also look good when they are finally published in the journal once they were prepared from the PACE tool like in our case.

**Reviewer #1:** I have no more comments. However, the quality and resolutions of figures listed in this manuscript are not good enough. I suggest authors should correct them.

**Response:** Thank you. However, the reviewer might not have opened the figure from the link provided at the top of each figure in the reviewer’s PDF document and appeared to have relied on what he/she saw directly on the reviewers PDF document to arrive at this concern. Please, we prepared our figures using the correct figure formats, including correct resolutions based on PLOS ONE recommended free tool (<https://pacev2.apexcovantage.com/>) and the quality and resolution was great on our systems. Always, the system that generates the reviewers document for review is responsible for the relatively low figure quality observed by the reviewer so reviewers must always download the actual figures from the link at the top of each figure in the reviewers PDF document which always have the required quality and resolution from the PACE tool as recommended by PLOS ONE.

However, we prepared new figures again using much higher resolution and used the PACE tool to format the figures as recommended by PLOS ONE to improve the quality and resolution as suggested.

Also, note that we now combined Figures 1 and 2 to form Figure 1 because they are both coming from the same model, and we thought is better to present them as one figure. Accordingly, we revised the manuscript to correct the figure numbers and added few text (see lines 270, 273, and 274 at page 10, line 285-286, and 294 at page 11, and lines 302-309 at page 12, lines 332 and 344 at page 13).

**Reviewer #2:** The revised manuscript has addressed all my concerns. I am happy to see it published in the journal.

**Response:** Thank you.
