# Supplementary material for: Multilevel analysis of predictors of multiple indicators of childhood vaccination in Nigeria
Source: PLoS One. 2022 May 25;17(5):e0269066. doi: 10.1371/journal.pone.0269066 (PMC9132327; doi:10.1371/journal.pone.0269066)
Supplement: Attachment — Submitted filename: Response to Reviewers_R3.docx [file pone.0269066.s005.docx]

Dear Editor,

Thank you for the opportunity to once again revise our manuscript titled “Multilevel analysis of predictors of multiple indicators of childhood vaccination in Nigeria”. We are extremely grateful to the Editors and the Reviewers for the constructive criticism of the manuscript and the positive feedback which certainly helped to improve the quality of our manuscript.

We have duly addressed all the concerns raised by the Editors and the Reviewers to the best of our ability and wish to submit the revised version for your consideration and subsequent publication in your cherished journal, and together we can all help in addressing this serious public health challenge facing millions of children globally, especially in developing countries like Nigeria.

**Response to Editorial comments**

**Journal Requirements:**

Please review your reference list to ensure that it is complete and correct. If you have cited papers that have been retracted, please include the rationale for doing so in the manuscript text, or remove these references and replace them with relevant current references. Any changes to the reference list should be mentioned in the rebuttal letter that accompanies your revised manuscript. If you need to cite a retracted article, indicate the article’s retracted status in the References list and also include a citation and full reference for the retraction notice.
**Response:** We reviewed our reference list and can confirm that they are correct and complete. Also, we did not find any retracted paper among our references. Thus, we did not make any changes to the manuscript and the reference list.

**Additional Editor Comments:**

The quality and resolutions of figures listed in this manuscript are not good enough. I suggest authors should correct them.

**Response:** Thank you for handling our manuscript smoothly and your decision for us to further improve our figures before our manuscript is accepted for publication.

We have prepared our figures following the recommended guidelines from PLOS ONE, including correct resolutions based on the recommended free tool (<https://pacev2.apexcovantage.com/>) and the quality and resolution was great on our systems (see original figures in the PLOS ONE submission system).

We believe our figures were produced with the required quality and resolution. However, we suspect that the Reviewers might not have accessed the original figures provided via the links at the top of each figure in the PDF proof of submission which was reviewed by the reviewers. This might have led them to persistently request that we produce new figures with high quality and resolution.

In our first revision, we responded to item 7 from reviewer #1 and the query was “Figures: The quality and resolution of figures are very bad. It’s hard to know the results.” Clearly, for reviewer #1 to say “… It’s hard to know the results”, the reviewer was only relying on the figures as appeared in the PDF Proof of submission (images in the PDF proof) rather than accessing the original figures embedded in the proof links above each figure. Nonetheless, we improved the quality and resolution of our figures in our revised submission dated on 3^rd^ March 2022, but the comments on the quality of figures came up in subsequent decision letter. Also, we have previously written to the Editor on 2^nd^ March 2022 but we are yet to receive any response except a new request to improve our figures.

As presented below, the reviewers seem to be basing their quality assessment of our figures on Fig 2 (i.e., the direct images shown in the pdf proof) instead of assessing Fig 1 (i.e., the original images embedded in the PDF proof links at the top right of each figure).

Fig 1 Screenshot of Figure 1 from the link on the top right of Figure 1 in the PDF Proof of submission.

Fig 2. Screenshot of the image shown in Figure 1 directly from the PDF Proof of submission.

Per the above evidence, our figures have good quality and resolution, and we believe strongly that our figures met PLOS ONE requirements to be accepted for publication. Thus, we did not make any changes to the figures after the second revision we have done to improve it further.

Note: we have now included acknowledgement section to our revised manuscript (see lines 456-457 at page 17).
